# Chemically Informed Coarse-Graining of Electrostatic Forces in Charge-Rich Biomolecular Condensates

**DOI:** 10.1021/acscentsci.4c01617

**Published:** 2025-02-11

**Authors:** Andrés R. Tejedor, Anne Aguirre Gonzalez, M. Julia Maristany, Pin Yu Chew, Kieran Russell, Jorge Ramirez, Jorge R. Espinosa, Rosana Collepardo-Guevara

**Affiliations:** † Yusuf Hamied Department of Chemistry, University of Cambridge, Lensfield Road, Cambridge CB2 1EW, United Kingdom; ‡ Department of Physical-Chemistry Universidad Complutense de Madrid, Av. Complutense s/n, Madrid 28040, Spain; § Department of Chemical Engineering, Universidad Politécnica de Madrid, José Gutiérrez Abascal 2, 28006 Madrid, Spain; ∥ Maxwell Centre, Cavendish Laboratory, Department of Physics, University of Cambridge, JJ Thomson Avenue, Cambridge CB3 0HE, United Kingdom; ⊥ Department of Genetics University of Cambridge, Cambridge CB2 3EH, United Kingdom

## Abstract

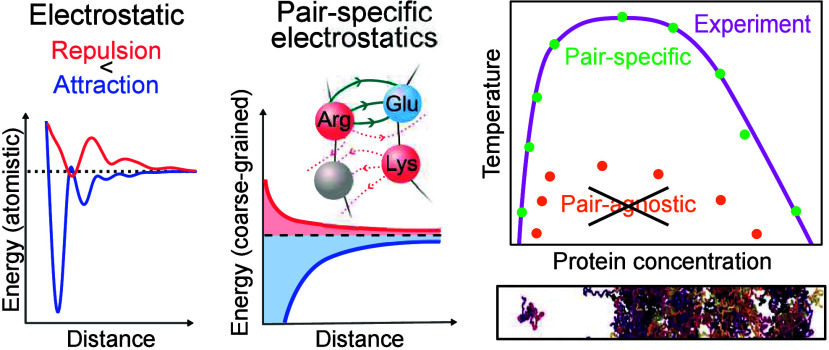

Biomolecular condensates composed of highly charged biomolecules, such as DNA, RNA, chromatin, and nucleic-acid binding proteins, are ubiquitous in the cell nucleus. The biophysical properties of these charge-rich condensates are largely regulated by electrostatic interactions. Residue-resolution coarse-grained models that describe solvent and ions implicitly are widely used to gain mechanistic insights into the biophysical properties of condensates, offering transferability, computational efficiency, and accurate predictions for multiple systems. However, their predictive accuracy diminishes for charge-rich condensates due to the implicit treatment of solvent and ions. Here, we present Mpipi-Recharged, a residue-resolution coarse-grained model that improves the description of charge effects in biomolecular condensates containing disordered proteins, multidomain proteins, and/or disordered single-stranded RNAs. Mpipi-Recharged introduces a pair-specific asymmetric Yukawa electrostatic potential, informed by atomistic simulations. We show that this asymmetric coarse-graining of electrostatic forces captures intricate effects, such as charge blockiness, stoichiometry variations in complex coacervates, and modulation of salt concentration, without requiring explicit solvation. Mpipi-Recharged provides excellent agreement with experiments in predicting the phase behavior of highly charged condensates. Overall, Mpipi-Recharged improves the computational tools available to investigate the physicochemical mechanisms regulating biomolecular condensates, enhancing the scope of computer simulations in this field.

## Abstract 2

Mpipi-Recharged is a residue-resolution coarse-grained model that innovatively treats screened electrostatic interactions, improving predictions for charged biomolecular condensates and ensuring computational efficiency.

## Introduction

Biomolecular condensates are ubiquitous intracellular assemblies formed via phase separation of biomolecular mixtures.^1−4^ Multivalency is a universal feature of phase-separating biomolecules, as it enables them to form dynamic, percolated networks that ensure the thermodynamic stability of the condensate.^5^ The greater the biomolecular valency, the denser the network of intermolecular connections that biomolecules can form, and the greater the enthalpic gain for condensate formation.^5^

While valency is universal to phase-separating biomolecules, the dominant types of intermolecular interactions that different multivalent biomolecules establish to decrease the free energy of their condensates are highly varied. These interactions depend not only on the chemical makeup and structural properties of the biomolecules themselves but also on those of their binding partners and on the parameters of the solution, such as salt concentration,^6−8^ temperature^9,10^ and pH.^11^ Moreover, the presence of molecular crowding agents,^12,13^ changes in post-translational modifications (PTMs),^14−16^ and fluctuations in metabolite concentrations^17−19^ are also potent modulators of the intermolecular interactions inside condensates. This capacity for regulation highlights the ability of condensates to exhibit specificity and selectivity in their composition and biophysical properties.

In some condensates, the essential multivalent biomolecules are rich in aromatic residues and arginines, such as the RNA-binding proteins with prion-like domains found in cytoplasmic stress granules.^2,20−23^ As a result, in such systems, π–π^9,24−28^ and cation−π^6,24,27,29−31^ interactions are the chief drivers of biomolecular phase separation, at least at physiological salt concentrations.^9,24,25,27,32,33^

In contrast, in condensates mostly filled with charge-rich molecules, charge–charge interactions can be the dominant regulators. This is observed, for instance, in complex coacervates^7,34−36^ and in most nuclear condensates.^37−39^ These condensates may contain mixtures of chromatin,^40,41^ nucleosomes,^37,42^ chromatin–binding proteins,^43^ negatively charged polyelectrolytes—e.g., DNA,^37−39,44^ RNA,^45−51^ and intrinsically disordered regions (IDRs) or intrinsically disordered proteins (IDPs) rich in glutamic and/or aspartic acids (e.g., prothymosin α)^52^—and positively charged polyelectrolytes—e.g., IDRs or IDPs rich in arginine and/or lysine (e.g., the histone tails of nucleosomes and the H1 protein).^7,34−36,52−55^ Additionally, they may contain polyampholytes, which are IDRs or IDPs like the N-tail of DDX4, possessing well-balanced distributions of both positively and negatively charged residues, and a low net charge per molecule.^56^

Understanding the molecular and biophysical mechanisms by which charge–charge interactions impact the properties of condensates is highly desirable but significantly challenging. Some charge-rich proteins, such as α-synuclein, undergo phase separation only in the presence of molecular crowders or at high salt concentration (where repulsive electrostatic interactions are sufficiently screened).^57−59^ Other charge-rich biomolecules phase separate only as part of multicomponent systems by establishing associative heterotypic interactions commonly of electrostatic nature (e.g., the mixture of H1 and prothymosin α).^7,45^

Computer simulations represent a powerful tool to probe the molecular mechanisms by which charge-rich biomolecules regulate the physicochemical properties of their biomolecular condensates. Among these tools, transferable residue-resolution coarse-grained models for biomolecular phase separation,^24,29,60−70^ such as the Mpipi family,^24,64^ the CALVADOS family,^61,62^ the HPS family,^29,60,66,71^ and MOFF,^72^ have gained recognition as they can effectively balance computational efficiency with adequate physicochemical accuracy. These models represent amino acids with a single bead, and define residue-pair interactions using a combination of short-range potentials—to model nonelectrostatic interactions and excluded volume (e.g., the Ashbaugh- Hatch version^73^ of the 12–6 Lennard-Jones potential^74^ for HPS and CALVADOS, and the Wang–Frenkel potential^75^ for Mpipi^24^)—and long-range Debye–Hückel potentials—to represent salt-screened electrostatic interactions.

Emergent properties of biomolecular solutions are estimated using residue-resolution coarse-grained models via direct coexistence molecular dynamics (MD) simulations^84^ or constant-pressure simulations of the condensed phase.^85,86^ These properties include temperature-vs-density phase diagrams,^6,29,62,76,77^ saturation concentrations,^76,78,79^ relative condensate stability,^24,61,78,80^ viscoelasticity and surface tension,^50,78,81−83^ and multiphase organization.^84–86^ Notably, simulations using the CALVADOS^61−63^ and the Mpipi models^24,64^ have achieved excellent agreement with experiments in measuring IDR ensemble properties, phase diagrams, and saturation concentrations.^24,27,64,77,87,88^

Some innovative aspects of the Mpipi coarse-grained model^24^ include: (a) the use of the Wang–Frenkel potential^75^ to describe nonelectrostatic associative interactions, (b) a parametrization derived from a combination of atomistic simulations of amino acid pairs^24^ and bioinformatics data,^89^ and (c) the replacement of the commonly used Lorentz–Berthelot combination rules—used to define the strength of nonelectrostatic interactions among amino acid pairs from the hydrophobicity scales of the pure amino acids^29,60−62,71^—for amino-acid-pair-specific parameters. These three features combined enhance the computational efficiency of the model and the parametrization flexibility. Additionally, they enable the model to accurately capture the dominant role of π-based contacts compared to non-π hydrophobic interactions, the markedly stronger associative contacts established by arginines over lysines, and the preference of arginine to bond with aromatic residues (cation-π) as well as with negatively charged species. As a result, the predictions of the Mpipi model for the temperature-vs-density phase diagrams of the variants of the IDR of the hnRNPA1 protein^24^ are in near-quantitative agreement with experiments.^9^ Furthermore, the predictions of Mpipi regarding the relative change in thermodynamic stability as a function of amino-acid mutations for several prion-like domain proteins are consistent with qualitative experimental trends.^27^ More recently, the Mpipi-GG model^64^ refined some of Mpipi’s Wang–Frenkel parameters, including stronger glycine–glycine and glycine–serine interactions, weaker aromatic–charge interactions, and generally stronger interactions for alanine, leucine, and isoleucine. After this, the deep learning algorithm ALBATROSS was developed using Mpipi-GG and used to predict ensemble dimensions of 137 IDRs in excellent agreement with experiments.^64^

Regarding the description of charge–charge interactions, even the most successful residue-resolution coarse-grained models, such as Mpipi^24,64^ and the CALVADOS family,^61,62^ have certain limitations.^6,7,37,66,68−70,88,90,91^ These limitations arise from the challenge of accurately capturing the impact of water and ions in the regulation of electrostatic forces in condensates, while describing these components implicitly in favor of computational efficiency. For example, the release of ions and water upon condensation is expected to play a crucial and complex role in regulating processes such as the salt-dependent modulation of protein condensate stability,^6,7^ the RNA-driven re-entrant phase behavior of protein condensates,^47^ the impact of charged patterning on condensates stability and preferential partitioning,^92−94^ and complex coacervation.^45^

In this work, we present the Mpipi-Recharged model, which improves the description of charge–charge interactions in biomolecular phase behavior, while maintaining the accuracy and performance of its predecessor.^24,27^ The new Mpipi-Recharged model describes charge–charge interactions with an asymmetric pair-specific Yukawa potential informed by potential of mean force (PMF) curves computed from umbrella sampling (US) atomistic MD simulations in explicit solvent and ions. These atomistic simulations reveal that, at the mean field level, the relative strength of electrostatic association and repulsion among charged amino acid pairs is substantially different; i.e., after averaging out over all degrees of freedom except for the intermolecular separation, the associative interactions between oppositely charged pairs are significantly stronger than the repulsive ones among like-charged pairs. We show that our pair-specific Yukawa potential successfully captures such asymmetry and allows Mpipi-Recharged to reproduce the phase behavior of highly charged proteins and complex coacervates.

We thoroughly validate the new Mpipi-Recharged coarse-grained model against a range of experimental measurements, including single-molecule radius of gyration, critical solution temperatures, and saturation concentrations. Additionally, we show that the Mpipi-Recharged model accurately captures experimental trends of various difficult-to-model highly charged systems, including salt-dependent phase diagrams, the impact of charge blockiness on the relative stability of condensates, and the stoichiometry thresholds observed for the various regimes of the RNA-driven re-entrant phase behavior for various RNA-binding proteins. Overall, the Mpipi-Recharged model exemplifies how incorporating a pair-specific asymmetric potential for electrostatic forces significantly improves the description of the phase behavior of highly charged systems while still treating solvent and ions implicitly.

## Results

### Atomistic Simulations Suggest Asymmetric Pair-Specific Coarse-Graining of Electrostatic Forces

**Figure 1:**
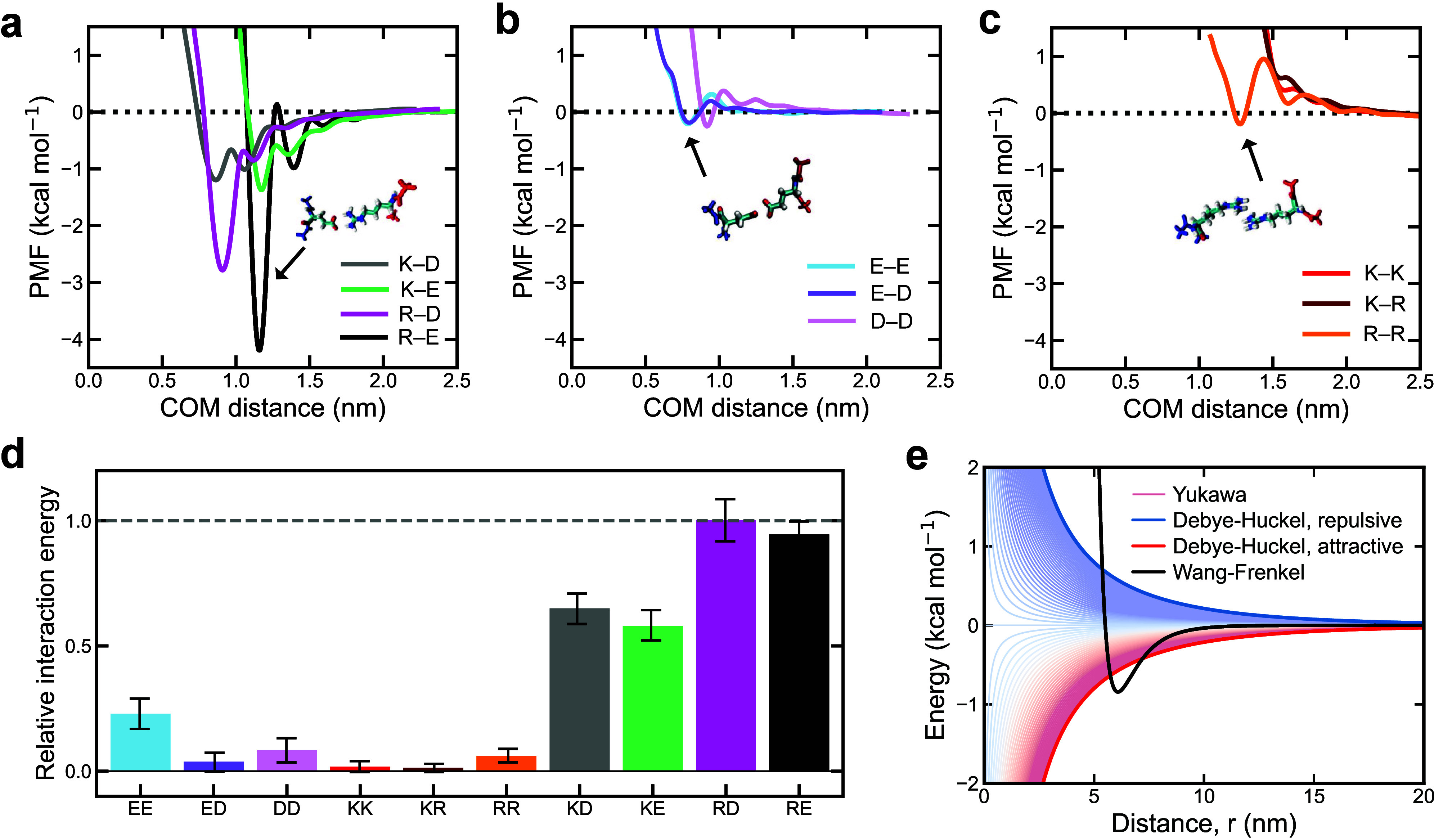
Free energies of binding between pairs of ±1 charged amino acids reveal a stronger role of associative versus repulsive electrostatic forces. (a–c) PMF computed from US atomistic MD simulations of charged amino acid pairs. The COM distance between the amino acids in the pair was used as the reaction coordinate. The simulations were performed with the a99SB-disp all-atom force field^95^ at T = 298 K and 150 mM NaCl salt concentration. The PMF plots shown are for: (a) positive–negative (Black: glutamic acid–arginine, E–R, Green: glutamic acid–lysine, E–K, Gray: lysine–aspartic acid, K–D, and Magenta: arginine–aspartic acid, R–D), (b) negative–negative (pink: aspartic acid–aspartic acid, D–D, Purple: glutamic acid–aspartic acid, E–D, and blue: glutamic acid–glutamic acid, E–E), and (c) positive–positive (Red: lysine–lysine, K–K, Maroon: lysine–arginine, K–R, and Orange: arginine–arginine, R–R) pairs of charged residues. (d) Integral of the attractive part of the PMF deepest attractive well as a function of the COM distance for the different curves presented in panels (a–c) normalized by the largest value of the set (R–D interaction). Error bars indicate the accumulated error of the integral extracted from the standard error of the PMF curves. (e) Schematic depiction of the potentials used in the Mpipi-Recharged model for hydrophobic/dispersive interactions (Wang–Frenkel potential; black thick curve) and electrostatic interactions (Yukawa potential; thin curves ranging from blue to red). Thick blue and red curves represent the standard profile of a Coulombic-like electrostatic screened potential (among species with charges equal to ±1) for repulsive and attractive interactions, respectively.

We first quantify the relative strengths of interactions between different pairs of charged amino acids by conducting atomistic US MD simulations. These simulations were performed with fixed amino acid orientations in explicit solvent and ions at 298 K, employing the a99sb-disp/JC-SPC/E-ion/TIP4*P*/2005 force field combination^95,96^ (see the Methods section for details). From the US simulations, we calculate the PMF of the amino pairs as a function of their pairwise center-of-mass (COM) distance.^6,18,28,77,79,97−101^ To elucidate the differences between attractive and repulsive forces, we computed a PMF for each of the ten unique pairwise combinations of the four amino acids with net charges (*q*) of +1e or −1e at pH = 7. These amino acids are arginine (R, *q* = +1e), lysine (K, *q* = +1e), glutamic acid (E, *q* = −1e), and aspartic acid (D, *q* = −1e). Hence, the combinations analyzed are aspartic acid–aspartic acid (D–D), glutamic acid–aspartic acid (E–D), glutamic acid–glutamic acid (E–E), lysine–lysine (K–K), lysine–arginine (K–R), arginine–arginine (R–R), arginine–glutamic acid (R–E), lysine–glutamic acid (K–E), lysine–aspartic acid (K–D), and arginine–aspartic acid (R–D) (Figure 1a–c). Each of these PMF curves describes the change in free energy experienced by a pair of amino acids, with their side chains oriented toward each other, as they move closer together from an infinite separation. This energy has been averaged out over all degrees of freedom of the system except for the one-dimensional intermolecular pairwise separation used as the reaction coordinate. The minimum value of each PMF can be considered as an approximate “binding free energy” (Figure S2). To define the relative interaction strength of the coarse-grained model parameters (Figure 1d), we compare the integrals of the attractive part of the corresponding PMF curve in Figure 1a–c.

Our simulations reveal that, at small molecular separations, pairs of oppositely charged amino acids (arginine–glutamic acid, arginine–aspartic acid, lysine–glutamic acid, and lysine–aspartic acid) experience a substantial free energy reduction due to the formation of associative electrostatic interactions (Figure 1a). This result is consistent with our previous simulations^6,18,24^ and with the critical role of associative electrostatic interactions in promoting the stability of biomolecular condensates observed experimentally.^6,7,45,46,102,103^ Additionally, our simulations show that, despite arginine and lysine having the same charge, arginine exhibits a greater propensity than lysine to form associative interactions with negatively charged amino acids (Figure 1a). The association of arginine with both glutamic acid and aspartic acid results in a notable free energy decrease—about −4.4 kcal/mol for arginine–glutamic acid and −3 kcal/mol for arginine–aspartic acid. In contrast, lysine shows a markedly smaller free energy decrease (up to −1.5 kcal/mol) upon binding to either glutamic acid or aspartic acid. The significantly more favorable binding free energies of arginine versus lysine with negative residues are consistent with experiments and simulations revealing a decreased phase separation propensity of protein solutions upon arginine to lysine mutation^24,27,104,105^ and marked differences in the properties of arginine-rich versus lysine-rich condensates.^6,9,45,106^ These results also align well with the higher free energy of hydration for arginine compared to lysine.^107^

In stark contrast, the PMF curves for pairs of like-charged amino acids (negative–negative pairs in Figure 1b and positive–positive pairs in Figure 1c) signal significantly weaker interactions compared to those for oppositely charged counterparts (positive–negative pairs in Figure 1a). When like-charged amino acids are brought closer together, the change in free energy exhibits low-height maxima (less than +1 kcal/mol), indicating weak repulsion. Interestingly, for some like-charged pairs, such as arginine–arginine (Figure 1c) or the three negative–negative pairs (Figure 1b), reducing the pairwise distance further (to approximately 0.9–1.2 nm) reveals an additional weakly attractive interaction, with a shallow minimum of around −0.1 kcal/mol. We attribute the minimal change in free energy upon binding of like-charged amino acid—especially when compared to the pronounced attraction observed between oppositely charged species—to several factors: (a) partial reorientation of amino acid side chains as the pairwise distance decreases; (b) changes in ion and amino acid solvation; and (c) alterations in the translational entropy of ions and solvent. As the COM distance decreases, oppositely charged amino acids, along with their surrounding water molecules and ions, reorganize into configurations that maximize Coulomb attraction. In contrast, for like-charged amino acids, this reorganization primarily minimizes repulsion. To emphasize this, we show the integrals of the deepest PMF minima with their associated standard errors in Figure 1d. Additionally, we summarize the relative binding free energies extracted from the deepest minima of each PMF curve in Figure S2. Both Figure 1d and Figure S2 demonstrate that electrostatic repulsion is considerably weaker than the attraction between oppositely charged residues.

### The Mpipi-Recharged Model

Using atomistic models to study biomolecular phase separation in charged biomolecules would naturally account for the asymmetric contribution of electrostatic interactions between like-charged and oppositely charged species we report in our PMFs (Figure 1), but their high computational cost makes this impractical. Residue-resolution coarse-grained models, like our model Mpipi,^24^ offer a more efficient strategy to probe biomolecular condensation computationally. However, residue-resolution coarse-grained models for biomolecular phase separation^24,29,60−65^ consider water and ions implicitly by invoking the Debye–Hückel mean-field approximation to describe electrostatic interactions.^108^ The Debye–Hückel approximation is necessary to reduce the degrees of freedom of the system and make the simulations of condensates feasible; yet, it assumes that the effects of monovalent counterions in solution can be reduced to the screening of the mean electrostatic potential generated by the amino acid (or nucleotide) charges. Therefore, the approximation considers that the level of screening and, thus, the decay of charge–charge interactions with distance is the same in magnitude for amino acid pairs with identical absolute total charges (i.e., all pairs discussed in Figure 1). That is because such decay is described by a Yukawa function that uses the same prefactor and screening length for all pairs of charged amino acids, regardless of their identity. Crucially, the notable differences in free energy of binding among like-charged and oppositely charged amino acids pairs that we have observed atomistically (Figure 1) cannot be recapitulated if the coarse-grained charge–charge interactions are described using the same Yukawa function parameters for all types of charged pairs. To overcome this limitation, here we developed a new residue-resolution coarse-grained model for biomolecular phase separation, named the Mpipi-Recharged model. The Mpipi-Recharged aims to improve the representation of charged biomolecules in our original Mpipi model while maintaining its exceptional performance for prion-like domain proteins.^24,27^

The Mpipi-Recharged model employs a Yukawa function with parameters that vary depending on the specific amino acid pair (see Figure 1e). Such a modification allows Mpipi-Recharged to consider that, at the coarse-grained level, attraction and repulsion should be described asymmetrically to compensate for the loss of explicit ions and water and for the aggressive mapping of the many charges that an amino acid carries atomistically to just one charge centered on its α carbon. To parametrize the pair-dependent Yukawa function, we use our atomistic PMF results shown in Figure 1 (see the Methods section for further details on the parametrization and model potentials). The resulting pair-specific parameters of the Yukawa functions are given in Table S3. A comparison between the Mpipi-Recharged model interactions and the atomistic PMF curves is given in Figures S15 and S16. In Table S4, we present the equivalence between the pair-specific prefactor (*A*_*ij*_) of the Yukawa potential and the effective charges required to reproduce the Yukawa potential with such a prefactor using a standard Coulomb/Debye–Hückel potential.

**Figure 2:**
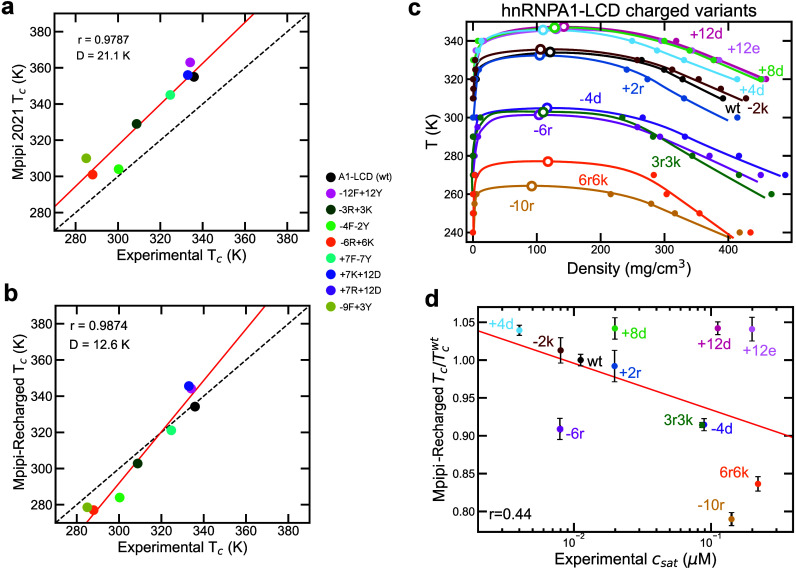
Predicted critical solution temperature *T*_c_ by the Mpipi (a) and Mpipi-Recharged (b) models against values estimated by us from experimental coexistence densities for hnRNPA1-LCD charged variants from ref (9). The Pearson correlation coefficient (*r*) and the root-mean-square deviation from the experimental values (*D*) are displayed for each set of modeling data. (c) Temperature-vs-density phase diagram for hnRNPA1-LCD charged variants. Solid symbols represent the obtained coexistence densities from DC simulations, and open symbols the estimated critical temperature through the law of rectilinear diameters and critical exponents.^109^ Continuous lines are included as a guide for the eye. (d) Predicted critical solution temperatures by the Mpipi-Recharged model against the experimental saturation concentration reported in ref (9) for the hnRNPA1-LCD charged variants. The simulated critical temperature has been normalized by the value *T*_c_^wt^ corresponding to wt-hnRNPA1-LCD.

To evaluate the performance of the new Mpipi-Recharged model, we subject it to the same rigorous tests previously used for the original Mpipi model.^24^ That is, we compare the model’s predictions against (1) single-molecule radius of gyration (*R*_*g*_) values of 46 different IDPs^9,110−121^ measured experimentally using small-angle X-ray scattering (SAXS) and Nuclear Magnetic Resonance (NMR) (Figure S2); and (2) experimental temperature-vs-concentration phase diagrams of hnRNPA1 Low Complexity Domain (hnRNPA1-LCD) variants.^9^ The amino acid sequences of the 46 IDPs and the corresponding experimental values of the R_*g*_ are provided in the Supporting Information. We find that, like the original Mpipi model, the Mpipi-Recharged model achieves excellent agreement with both the experimental *R*_*g*_ values (Figure S2) and the phase diagrams^9,24^ (Figure 2a–b).

Additionally, we compare critical solution temperatures predicted by the Mpipi-Recharged model (Figure 2c-d) against the experimental saturation concentrations for various hnRNPA1-LCD charge variants (*C*_*sat*_).^9^ Such a comparison assumes that the simulation predictions of critical solution temperature and the experimentally measured saturation concentrations are both good proxies of condensate thermodynamic stability.^27,78^ Capturing the phase behavior of the hnRNPA1-LCD charge variants is a stringent test, as mutating just a few charged residues can significantly alter the coexistence densities and saturation concentrations.^9,10^ Charge mutations can either destabilize or promote protein condensation, depending on the characteristics of the introduced residues and the sequence context.^9,27,122,123^ Indeed, experimental saturation concentrations for hnRNPA1-LCD charge variants differ by more than two orders of magnitude, highlighting the dramatic effects of these mutations.^9,10^ Importantly, we find that while the original Mpipi model shows a moderate correlation between predicted critical temperatures and experimental saturation concentrations for the hnRNPA1-LCD charge variants (Figure S4), the Mpipi-Recharged model improves the correlation coefficient between these two quantities by 50% (Figure 2d). Moreover, the Mpipi-Recharged model demonstrates greater sensitivity to charged residue mutations, as reflected in the predicted critical solution temperatures of the charge variants, which span nearly twice the temperature range compared to those predicted by the Mpipi model (Figure S4). This enhanced sensitivity suggests better qualitative agreement with the broad range of experimental saturation concentration values displayed by the charge variants.

Understanding the impact of charge blockiness—defined as the clustering of like-charged monomers along a polymer sequence^124,125^—is critical for studying biomolecular condensate stability. Yet, capturing this effect with residue-resolution coarse-grained models is particularly challenging when solvent and ions are treated implicitly.^91^ These models inherently overlook that the distribution of charges within a protein affects the organization and dynamics of ions condensed around the protein, the correlations between these ions, the screening of bare biomolecular charges by these ions, and the entropic gain upon ion release during formation of biomolecular interactions. Despite these limitations, models like Mpipi-Recharged can mitigate some of the effects arising from the lack of an explicit representation of ions through appropriate parametrization. Therefore, we now test how well Mpipi-Recharged performs when predicting the phase behavior of the wild-type (WT) N-terminal domain (NTD) of the DEAD-Box helicase 4 (DDX4) protein, a highly charged IDR with pronounced charge blockiness.^29,91^ We compare the behavior of the WT protein with three variants: a charge-scrambled (CS) variant, which maintains the same total charge but redistributes the charged residues uniformly; an arginine-to-lysine (RtoK) variant; and a phenylalanine-to-alanine (FtoA) variant. The phase behavior of the WT and the three variants has been extensively studied experimentally^89,103,104^ and with theory and simulations.^24,29,91,126^

**Figure 3:**
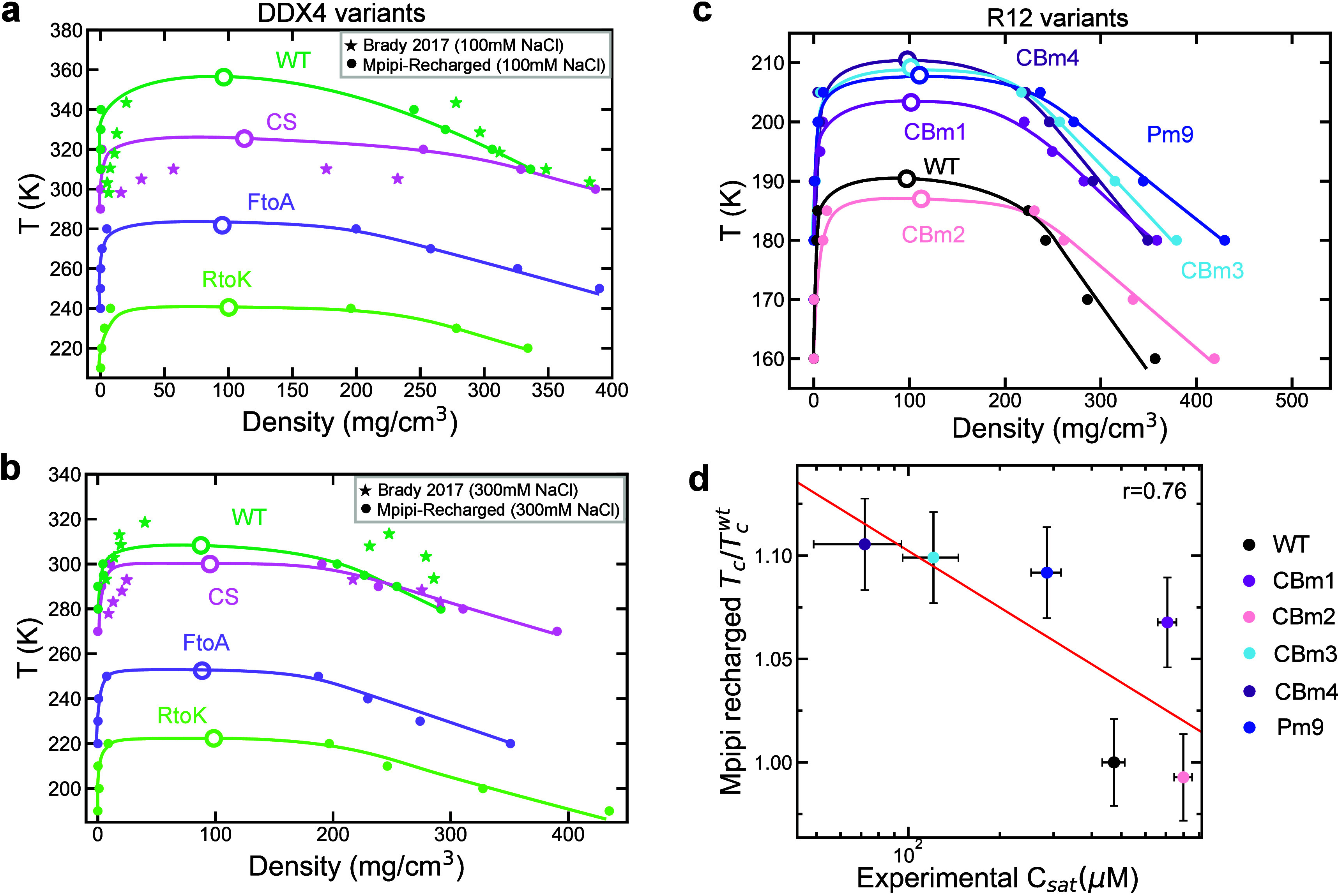
Predicted phase-separation behavior of DDX4 and R12 variants by the Mpipi-Recharged model. Phase diagram in the temperature-vs-density plane, at 100 mM NaCl in panel (a) and at 300 mM NaCl in panel (b), for DDX4 variants as studied in ref (103), obtained using direct coexistence simulations and the Mpipi-Recharged model. Solid circles indicate the equilibrium coexistence densities of each phase, empty symbols show the critical solution temperature predicted from simulations, and solid stars represent experimental data from Brady et al.^103^ for the WT and CS variants as indicated by the color code. (c) Phase diagram in the temperature-vs-density plane for R12 variants with different charged blockiness obtained using direct coexistence simulations and the Mpipi-Recharged model. Solid symbols indicate the equilibrium coexistence densities of each phase and empty symbols represent the critical solution temperature. (d) Critical solution temperature from simulations vs experimental saturation concentration *C*_sat_ for the same R12 variants.^46^ The critical solution temperature is normalized by the value *T*_c_^wt^ corresponding to the wild-type R12 sequence.

Experiments have established that, at 100 mM monovalent salt, the relative propensities of DDX4 variants to phase separate are as follows: WT > CS > FtoA > RtoK.^89,91,103,104^ Experimental phase diagrams mapping temperature versus concentration at various salt concentrations have also been measured for the WT and CS variants.^103^ These experiments reveal a critical solution temperature difference of approximately 50 K between the WT and CS variants at 100 mM NaCl.^103^ When comparing the Mpipi-Recharged model predictions with the experimental phase diagrams at this salt concentration, the model demonstrates near-quantitative accuracy (Figure 3a), predicting a 40 K difference in critical temperatures between the WT and CS variants. In contrast, the original Mpipi model struggles to differentiate between the two variants, predicting a critical temperature difference of only 10 K (Figure S5). At 300 mM NaCl, where experimental data show that the critical solution temperatures for both variants decrease^103^ and the difference narrows to about 10 K, the Mpipi-Recharged model continues to closely match experimental results (Figure 3b).

To investigate the importance of coarse-graining electrostatic attraction and repulsion asymmetrically, we repeated simulations for the DDX4 WT, CS, FtoA, and RtoK variants at 100 mM NaCl using a modified version of the Mpipi-Recharged model. In this modified version, termed Mpipi-Recharged-symmetric, a pair-agnostic symmetric Debye–Hückel potential, similar to that used in the original Mpipi model,^24^ replaces the asymmetric pair-specific Yukawa potential. As shown in Figure S6, switching from the pair-specific asymmetric to a pair-agnostic symmetric coarse-graining of electrostatic interactions in the Mpipi-Recharged model results in a significant underestimation of the critical solution temperatures for all DDX4 variants. Moreover, while the relative phase-separation trend is preserved at 100 mM NaCl, at 300 mM NaCl the CS variant is predicted to have a higher critical temperature than the WT sequence, contradicting in vitro observations.^103^ These results highlight that coarse-graining electrostatic forces asymmetrically is important to accurately predict the phase behavior of highly charged protein solutions, such as those of DDX4 (Figure 3a,b).

Another phase-separating system where charge blockiness plays a crucial role is the R12 segment of the Ki-67 protein.^46^ Experiments analyzing the phase behavior of different R12 variants report a strong correlation between the protein saturation concentration and the degree of charge blockiness.^46^ In Figure 3c, we use Mpipi-Recharged to compute the phase diagram in the temperature-vs-density plane for six variants of R12 (amino acid sequences provided in the Supporting Information) from direct coexistence^84^ simulations. As in the case of DDX4, we find excellent agreement between the predicted propensity to undergo phase separation for each variant (extracted from the *T*_c_ in our simulations) and the experimental saturation concentration reported in ref (46) (Figure 3d). Overall, these tests confirm the adequacy of coarse-graining charge–charge interactions asymmetrically to improve the predictions for highly charged condensates, particularly after considering the erroneous results predicted by the Mpipi-Recharged-symmetric test model (Figure S5).

### Mpipi-Recharged Captures the Regulation of the Stability of Complex Coacervates with Salt

Complex coacervates are charge-rich multicomponent droplets formed via phase separation of mixtures of oppositely charged polyelectrolyte biomolecules. The stability of complex coacervates is predominantly sustained by heterotypic electrostatic interactions among the different components.^7,34−36,53−55^ Not surprisingly, the biophysical properties of complex coacervates, and more generally of charge-rich multicomponent condensates, are intricately regulated by changes in their composition and the stoichiometry of the various components. This is because those changes can drastically alter the fine balance between attractive versus repulsive electrostatic interactions.^47,48,51,127−129^ In fact, monotonic changes in the stoichiometry of the species of multicomponent condensates can lead to striking nonlinear changes in their stability.^26,47,48,51,127−130^ For example, RNA–protein condensates are known to exhibit an RNA concentration-dependent re-entrant phase transition, where condensates present low stability at low and high RNA concentrations, and maximum stability at intermediate RNA concentrations.^47,48,51,127−129^

Describing the phase behavior of complex coacervates is a particularly difficult, yet important, goal for a residue-resolution coarse-grained model. To test the ability of Mpipi-Recharged to probe complex coacervation, we use it to simulate a two-component mixture made of two highly charged proteins: H1 and Prothymosin-α (ProTα). H1 is a positively charged multidomain protein, which consists of a short disordered N-terminal tail, a globular domain, and a long (∼100 amino acid) disordered positively charged C-terminal tail.

To treat proteins like H1, we first focus on adapting the Mpipi-Recharged model for multidomain proteins. Consistent with previous studies, we assume that the secondary structure of globular domains within these multidomain proteins remains stable throughout the simulations.^6,24,63,71,78^ Therefore, we describe globular domains as rigid bodies.^6,24,63^ To achieve this, we use an experimental atomistic structure (e.g., from the PDB or AlphaFold) as a reference. The relative positions of all interaction sites within the globular domain are maintained, with beads centered on the α carbon.^6,24,71,78^

Previous studies^6,24,29,76,78^ have modeled globular domains as rigid bodies, rescaling their interactions by a fixed factor (e.g., 0.7 in the Mpipi model^24^). However, no systematic effort has been made to evaluate how variations in the rescaling factor affect the accuracy of predictions relative to experimental measurements of protein solutions. In this work, we address this by titration of two different rescaling factors that reduce the contribution of the Wang–Frenkel potential to the total interaction energy for residues within globular domains.

**Figure 4:**
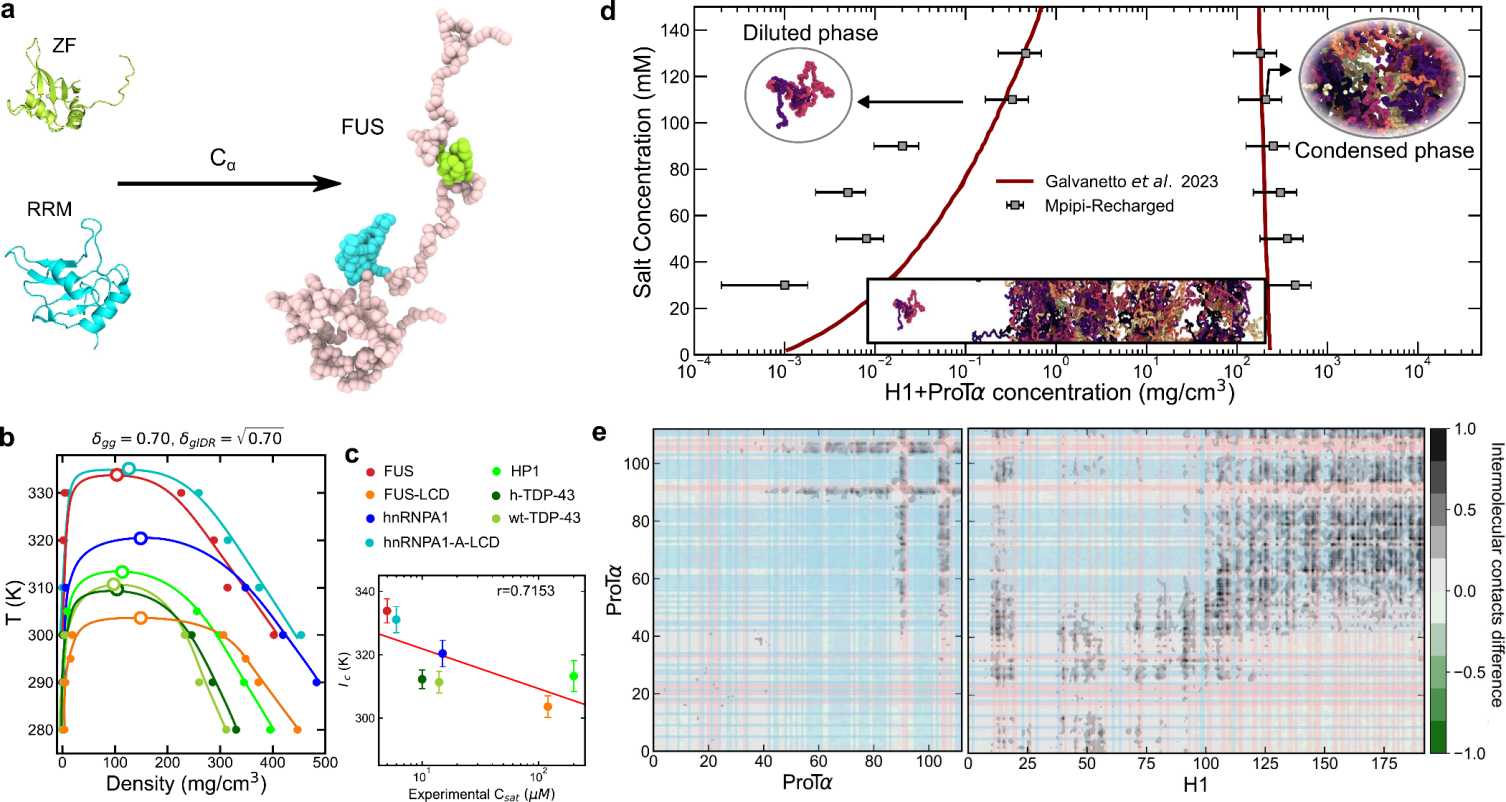
(a) Schematic representation of the globular domains of FUS from the PDB structures to the coarse-grained representation of the Mpipi-Recharged model. Globular domain (as those depicted by cyan and light green beads for FUS) interactions have been gradually scaled down to optimize the correlation between the model critical temperature and the experimental saturation concentration. (b) Phase diagram from direct coexistence simulations for multidomain proteins FUS (red), hnRNPA1 (blue), HP1 (lime green), h-TDP-43 (dark green), and wt-TDP-43 (olive green) and intrinsically disordered proteins FUS-LCD (orange) and hnRNPA1-LCD (light blue). Solid symbols indicate the equilibrium coexistence densities of the two phases, and empty symbols display the critical temperature for each system. δ_gg_ refers to factor by which Wang–Frenkel interaction strength for globular–globular interactions are rescaled, while δ_gIDR_ scales down interactions between a globular domain bead and an IDR bead. (c) Critical temperature from simulations using the parameters indicated in panel (b) vs the experimental saturation concentration *C*_sat_ for FUS,^51,131,132^ FUS-LCD,^132^ hnRNPA1,^133^ hnRNPA1-LCD,^9,134^ HP1,^43^ and TDP-43.^21,135^ (d) Phase diagram of a 1:1.2 H1–ProTα mixture in the salt concentration (KCl)-vs-concentration plane at *T* = 273 K as predicted by the Mpipi-Recharged model (gray squares). *In vitro* results for the same system reported by Galvanetto et al.^7^ are depicted by a red line. Representative snapshots are provided for the simulation slab (bottom), the condensed phase (right), and the dilute phase (left). (e) Intermolecular contact frequency difference (in number of contacts per residue) between ProTα–ProTα (left) and H1–ProTα (right) between the system at 30 mM and 130 mM KCl concentration. The thick lines across the panels indicate the positively charged (red) and negatively charged (blue) blocks in ProTα and H1 sequences.

The first factor, δ_gg_, reduces the Wang–Frenkel interaction strength for globular–globular interactions, while the second factor, δ_gIDR_, scales down interactions between a globular domain bead and an IDR bead. During the titration process, we varied the values of δ_gg_ and δ_gIDR_ from 0.75 to 0.65 and evaluated their effects on the predicted critical solution temperature using the Mpipi-Recharged model, correlating the results with experimental saturation concentrations. We previously demonstrated that critical solution temperature and saturation concentration are strongly inversely correlated (e.g., lower critical solution temperatures correspond to higher saturation concentrations).^88^ For this assessment, we focused on the phase behavior of five multidomain proteins with available experimental data: FUS,^51,131,132^ hnRNPA1,^133^ HP1,^43^ TDP-43^21,135^ (wild-type TDP-43), and a TDP-43 variant with an α-helix motif in its C-terminal tail (h-TDP-43).^135^ Detailed information on the atomistic structures used to construct each coarse-grained protein model can be found in the Supporting Information. Additionally, Figure 4a provides a schematic depiction of the model for FUS. Our analysis (Figure S7) reveals that the strongest correlation between experimental saturation concentrations and the critical solution temperatures predicted by the Mpipi-Recharged model is achieved with δ_gg_ = 0.7 and  (Figure 4b).

Additionally, we tested an alternative approach recently proposed in ref (63) within the CALVADOS3 model. Instead of rescaling the interactions within globular domains, the CALVADOS3 model shifts the center of the globular domain beads from the C_α_ atom to their COM. While positioning the globular beads at the COM yields excellent results in the CALVADOS3 model, we find that for Mpipi-Recharged it results in a significantly weaker correlation between the predicted critical solution temperature and the experimental saturation concentration (Figure S8), compared to using the 0.7 rescaling factor. We hypothesize that this discrepancy stems from the differing treatments of charge–charge interactions between the Mpipi-Recharged and CALVADOS3 models.

After establishing the modeling strategy for multidomain proteins, we use the Mpipi-Recharged to probe the complex coacervation of the H1–ProTα system. Adequately capturing the balance of electrostatic attraction and repulsion in the H1–ProTα system is challenging because both proteins have very high net charges: H1 has a net charge of +53e, while ProTα has a charge of −44e. Inspired by the experiments of Galvanetto et al.,^7^ we perform direct coexistence simulations of a H1–ProTα mixture with 1:1.2 stoichiometry, which is nearly electroneutral. We vary the monovalent salt concentrations by varying the values of the Yukawa potential screening length (see the Methods section). From these set of simulations, we compute the phase diagram of the H1–ProTα mixture in the plane of monovalent salt concentration versus total biomolecular concentration or density, which we show in Figure 4d. Reassuringly, Mpipi-Recharged reproduces the experimental phase diagram (red curve)^7^ with near-quantitative accuracy. Naturally, the simulation predictions of the concentrations of the diluted phase deviate the most from the experimental values because estimating such low concentrations from direct coexistence simulations is inadequate due to the amplification of finite-size effects in the diluted phase.

As we did for DDX4 (Figure S6), we demonstrate the importance of describing electrostatic attraction and repulsion asymmetrically to accurately predict the salt-dependent phase behavior of the H1–ProTα system by repeating simulations with the Mpipi-Recharged-symmetric test model. In these simulations, the Mpipi-Recharged-symmetric model fails to predict phase separation at any of the studied salt concentrations, likely due to an overestimation of repulsive interactions (Figure S10). To further validate this observation, we tested an alternative model, the HPS-cation-π model, under the same conditions. The HPS-cation-π model significantly underestimates both the coexistence densities and the critical salt concentration of the H1–ProTα system, predicting no phase separation above ∼100 mM NaCl. This again points to an overestimation of repulsive interactions in the model (Figure S10). Together, these results indicate that residue-resolution coarse-grained models employing a symmetric Debye–Hückel potential tend to overestimate the role of electrostatic repulsion in the phase behavior of protein solutions. These findings highlight the importance of incorporating an asymmetric description of electrostatic interactions in implicit solvent coarse-grained models to achieve a proper balance between repulsion and attraction, thereby improving the accuracy of the models in reproducing experimental observations of charged protein solutions.

We further investigate the impact of salt concentration on the phase behavior of the H1–ProTα coacervates by evaluating the intermolecular pairwise contact frequencies among the different amino acids in the two proteins. In Figure 4e we show the variation (in number of contacts per residue; further details provided in the Methods section) of the number of intermolecular contacts from 130 mM to 30 mM monovalent salt concentration. Our homotypic intermolecular contact maps for ProTα–ProTα (Figure 4e; Left) and heterotypic contact maps for ProTα–H1 (Figure 4e; Right) show a considerable enhancement between oppositely charged residue contacts (see red (positively charged) and blue (negatively charged) blocks in the chart axes of Figures 4e and S9) as the monovalent salt concentration decreases. These results reinforce studies showing how the stability of H1–ProTα condensates is mainly sustained by electrostatic interactions, and how relatively small variations in salt concentration can strongly regulate their formation and dissolution.^6,7,45,68,103,129^ The most frequent intermolecular contacts sustaining H1–ProTα condensates are reported in Figure S18. Importantly, while attractive electrostatic interactions such as K–D or K–E substantially contribute to enabling phase separation of H1–ProTα, complex coacervates formed by short charged polypeptides (e.g., polyK(10)-polyD(10) as shown in Figure S17) cannot undergo phase separation under physiological conditions solely driven by electrostatic interactions. These results highlight the context-dependent nature of electrostatic interactions, which are more effective at driving condensate-stabilizing contacts in desolvated environments.

The excellent agreement between the predictions of the Mpipi-Recharged model with the experimental data for the H1–ProTα system further highlights the adequacy of using an asymmetric pair-specific electrostatic potential to improve the description of the phase behavior of highly charged systems.

### Mpipi-Recharged Accurately Captures the RNA-Re-Entrant Behavior of RNA-Binding Proteins

**Figure 5:**
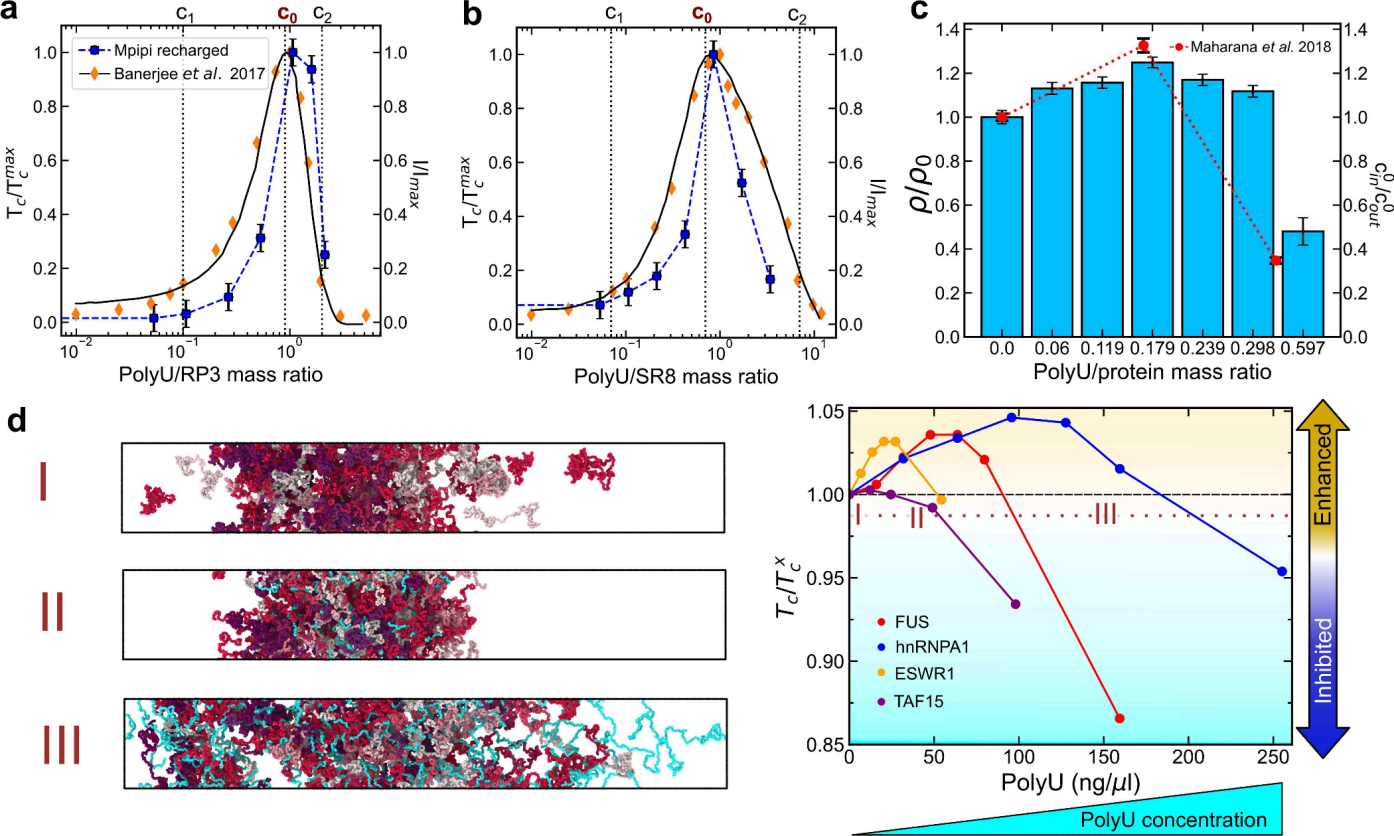
Predictions of the RNA-driven re-entrant phase behavior of protein condensates by the Mpipi-Recharged model. Comparison of simulated critical solution temperature (blue symbols) with *in vitro* solution turbidity experiments^47^ (yellow symbols) as a function of the polyU/peptides mass ratio for RP3 (a) and SR8 synthetic peptides (b). Both simulation critical temperatures (*T*_c_) and fluorescence intensities (*I*) are normalized by the maximum value of the set. The phase regimes indicated by *C*_1_, *C*_0_, and *C*_2_ are extracted from the work of Banerjee et al.^47^ as explained in the text. (c) Bulk density of polyU–FUS condensates at 300 K from NpT simulations (at 0 bar and 300 K) as a function of the polyU/protein mass ratio. Red symbols represent the *in vitro* protein partition coefficient for different polyU–FUS mixtures normalized by that of a pure FUS solution as reported by Maharana et al.^51^ The computed density from simulations with different concentrations of polyU was renormalized by that of pure FUS condensates at the same conditions. (d) Right: RNA re-entrant behavior for different multidomain RBPs (as indicated in the legend) showing the variation in the critical temperature (normalized by that of pure protein condensates, *T*_c_^x^) as a function of the polyU concentration. Dashed line sets the separation between promoting and hindering phase separation. Left: Representative snapshots of direct coexistence simulations of FUS with different polyU concentrations (as indicated in the left panel) and at a temperature of *T* = 0.98*T*_c_^FUS^ (depicted by the horizontal dotted line in the right panel).

Seminal work^47^ by Banerjee and colleagues first discovered that complex coacervates made by short positively charged synthetic peptides (SR8 and RP3) and single-stranded RNA (polyU) exhibit an RNA concentration-dependent re-entrant phase transition. Specifically, they found that the peptide–RNA mixtures only form complex coacervates at intermediate polyU concentrations. When the concentration of polyU was too low (i.e., *C* < *C*_1_, where *C* is the polyU concentration and *C*_1_ the low polyU concentration threshold) or too high (i.e., *C* > *C*_2_, where *C*_2_ is the high polyU concentration threshold), phase separation did not take place (Figure 5a-b; orange diamonds). Furthermore, at intermediate polyU concentrations (*C*_1_ < *C* < *C*_2_), the stability of the condensates reached a maximum when the stoichiometry of the mixture was slightly beyond the electroneutral point (*C*_0_). Nonmonotonic changes in the stability of multicomponent condensates have also been reported for mixtures of FUS-PLD and hnRNAPA1-LCD,^26^ and mixtures of PRC1 and RING1B.^130^ As in the case of protein–RNA mixtures, in both of these additional examples, associative electrostatic interactions between the two components were identified as the driving forces for the phenomena.

To test whether the Mpipi-Recharged model can reproduce the experimental RNA-driven re-entrant phase behavior of peptide–polyU mixtures observed experimentally by Banerjee et al.,^47^ we derive parameters to describe polyU within our Mpipi-Recharged model using a resolution of one-bead per nucleotide. To obtain an estimation of the parameters of the Wang–Frenkel and Yukawa potentials for the model, we perform atomistic umbrella sampling simulations of different pairs of amino acids with uracil (U) (Figure S1). We used the AMBER ff03ws force field both for the amino acids and for the RNA nucleotides.^136^ Further details on the simulations and parameter derivations can be found in the Methods section and the Supporting Information. These atomistic simulations are used as a reference to adjust the parameters of the Mpipi-Recharged model for RNA.

Using Mpipi and Mpipi-Recharged, we perform MD simulations of the condensed liquid phases at constant pressure (0 bar) for SR8–polyU mixtures and RP3–polyU mixtures at various stoichiometries. From these simulations, we estimate the critical solution temperatures, which provide a quantitative measurement of condensate stability. We have previously demonstrated that pure condensed liquid simulations at constant pressure (e.g., 0 bar) allow for the estimation of critical solution temperatures for multicomponent mixtures at fixed stoichiometries, unlike direct coexistence simulations where controlling the condensate composition is difficult.^79,85^

We compare the values of the critical solution temperatures predicted by Mpipi (Figure S11) and Mpipi-Recharged (Figure 5a,b) against the experimental values of fluorescence intensities reported by Banerjee et al.^47^ These values are normalized by the maximum value of *T*_c_, which occurs at concentration *C*_0_. The comparison is made for two different peptide–polyU mixtures as a function of the polyU/peptide mass ratio, and the results are contrasted with the corresponding experimental values of fluorescence intensities (normalized by maximum intensity at *C*_0_). Figure 5a-b demonstrates that Mpipi-Recharged improves upon the already very good predictions of the original Mpipi model. Notably, Mpipi-Recharged not only recapitulates the RNA-driven re-entrant phase behavior of both polyU–RP3 and polyU–SR8 solutions—as does the original Mpipi (Figure S11)—but also predicts the concentration thresholds at which maximum stability of the complex coacervates is observed (i.e., *C*_0_) in quantitative agreement with the experimental values. We verify the importance of our asymmetric electrostatic potential by performing simulations with the HPS-cation-π model (Figure S12). These simulations reveal that the HPS-cation-π model predicts phase separation of polyU–RP3 and polyU–SR8 mixtures only near the electroneutral point. Notably, the HPS-cation-π model fails to capture the gradual change in condensate stability with increasing RNA concentration. Instead, it exhibits sharp transitions: a very narrow difference between C_1_ and C_0_ marks the change from no phase separation to maximum stability, and another narrow difference between C_0_ and C_2_ signals the change from maximum stability back to its dissolution. This behavior contrasts starkly with experimental observations, where fluorescence intensities vary gradually across a much broader range of RNA concentrations. The discrepancy between the HPS-cation-π results and the experiments likely arises from an imbalance between R–R and U–U electrostatic repulsion and R–U attraction in the model. These results underscore the importance of describing attractive and repulsive electrostatic interactions nonsymmetrically at the coarse-grained level to achieve better agreement with experimental behavior.

To further test the performance of Mpipi-Recharged, we examine the behavior of polyU and FUS mixtures, which are also known to exhibit an RNA-driven re-entrant phase transition.^51,132,137^ Inspired by the experimental setup of Maharana et al.,^51^ we perform constant-pressure condensed-liquid simulations of mixtures of 250-nucleotide polyU and the 526-residue FUS protein at various stoichiometries. From these simulations, we measure the equilibrium coexistence densities of the condensed liquids at equilibrium (ρ) as a function of the mixture stoichiometry. The density of the condensed phase directly relates to the stability of the condensates; i.e., higher density implies higher *T*_c_,^78,138^ and thus, higher condensate stability. We normalize these condensed liquid densities by dividing them by the density of the pure FUS condensed liquid (ρ_0_). The normalized density indicates the relative stability of the different polyU–FUS condensates compared to the pure FUS condensates. We then compare the normalized condensed liquid densities from our simulations with the experimentally measured partition coefficient of FUS.^51^ The partition coefficient of FUS is defined as the concentration of FUS inside the condensate (*C*_*in*_) divided by the concentration of FUS in the diluted phase (*C*_*out*_) and, as in the original study, it is presented normalized by the value of the partition coefficient in the pure FUS system (*C*_*in*_^0^/*C*_*out*_^0^). While the original version of Mpipi qualitatively captures the experimental trends,^77,85,86^ Mpipi-Recharged, additionally quantitatively predicts the optimal RNA concentration that enhances the stability of polyU–FUS condensates. Moreover, Mpipi-Recharged also correctly predicts the polyU concentration at which the condensates become less stable than those of pure FUS (Figure 5c).

Finally, we used Mpipi-Recharged to compare the RNA-driven re-entrant phase behavior of condensates containing 250-nucleotide polyU with one of the following proteins: EWSR1, TAF15, FUS, or hnRNPA1. For this, we computed the critical temperature of the different protein–polyU condensates as a function of polyU concentration (Figure 5d). Our simulation results capture the observation of low stability at both low and high polyU concentrations, and high stability at intermediate polyU concentrations for all the four mixtures. Consistently with experimental fluorescent microscopy images, we observe that condensates made from either EWSR1 or TAF15 proteins dissolve at much lower polyU concentrations than hnRNPA1 and FUS condensates. As in the experiments,^51^ our simulations show that hnRNPA1 is capable of recruiting much higher amounts of polyU, followed by FUS, before condensate dissolution. The polyU/protein mass ratio threshold at which condensates stability begins to decrease, in all cases, falls very close to the value that produces electroneutral mixtures; that is, ∼ 11 ng/μL for ESWR1, ∼ 7 ng/μL for TAF15, ∼ 25 ng/μL for FUS, and ∼50 ng/μL for hnRNPA1 (for the protein concentration given in ref (51)). These results suggest that the competition between electrostatic association between proteins and RNA, and the electrostatic repulsion among RNAs as a function of RNA concentration inducing the RNA-driven re-entrant phase behavior observed are well-captured by the Mpipi-Recharged model.

### Limitations of the Mpipi-Recharged Model

The Mpipi-Recharged model is specifically designed to describe the phase behavior of IDPs, multidomain proteins containing IDRs and globular domains, as well as disordered single-stranded RNAs. Its primary advantage is that it enhances the quantitative and qualitative characterization of the phase behavior of charged protein solutions while maintaining the computational efficiency of its predecessor. However, Mpipi-Recharged shares limitations with other state-of-the-art residue-resolution coarse-grained models, including Mpipi, CALVADOS, and HPS.

These models provide significantly higher computational efficiency compared to atomistic simulations and explicit-water coarse-grained models and are particularly effective at capturing the effects of amino acid sequence mutations on the stability and material properties of condensates. Despite their efficiency, residue-resolution coarse-grained models remain limited in their ability to explore the phase behavior of large biomolecules, such as proteins exceeding approximately 500 amino acids or RNAs with hundreds of nucleotides, as well as multicomponent solutions with more than four components, due to constraints related to finite-size effects and the computational demands of such complex systems.

Another limitation of residue-resolution coarse-grained models is that they treat IDPs/IDRs as fully flexible polymers, hence ignoring their intricate structural behavior. Furthermore, globular domains are treated as rigid bodies or with elastic network models, which overlook dynamic conformational rearrangements that can influence phase behavior.^139^ Recently, a residue-specific angular, dihedral, and correction map potential was used to improve backbone conformations of proteins in the Mpipi model.^140^

Residue-resolution coarse-grained models rely on simplified interaction potentials, such as Lennard-Jones or Wang–Frenkel terms, which may not fully capture sequence specificity. Multibody potentials may be needed to address this limitation because specific interactions often involve cooperative binding of multiple residues, hydrogen bonding, ion-mediated bridging, and charge clustering.

While residue-resolution coarse-grained models can predict equilibrium phase diagrams, they often fail to capture nonequilibrium behavior of condensates, like their liquid-to-solid transitions and the emergence of arrested states. Nonetheless, Mpipi has been successfully used to investigate the molecular mechanisms behind liquid-to-solid transitions and kinetic arrest of condensates by being paired with an advanced dynamic algorithm^77,98^ that introduces nonconservative changes to the forces as a function of time.

Electrostatic interactions play a crucial role in determining the phase behavior of biomolecular condensates, particularly in systems dominated by charged biomolecules. Most residue-resolution coarse-grained models, such as the original Mpipi model, use pair-agnostic Debye–Hückel potentials to describe salt-screened electrostatic interactions. This approach often overestimates repulsive forces between like-charged residues while underestimating attraction between oppositely charged residues. Mpipi-Recharged addresses this limitation by incorporating an asymmetric pair-specific Yukawa potential informed by atomistic PMF results, reproducing the phase behavior of charged biomolecular condensates in better agreement with experiments than its predecessor (Figures 2-5).

Despite improving the description of electrostatic effects with respect to its predecessor, due to its mean-field treatment of water and ions, Mpipi-Recharged still faces the following limitations. The model ignores ion-specific effects, such as the Hofmeister series or ion-bridging, and the role of multivalent ions, which are likely critical in biomolecular condensates in cells.

Ions may partition asymmetrically into condensates and the dilute phase, creating salt concentration gradients that can influence condensate stability.^102^ By invoking a Yukawa potential, Mpipi-Recharge considers that counterions in solution lead to uniform screening across the whole system, which ignores that screening is likely distinct in different regions of the condensate (e.g., bulk vs interphase) and between the condensate and the dilute phase. In addition, while the effective charge of biomolecules can vary in biomolecular condensates due to factors such as changes in pH, salt concentration, or the proximity of charged species, the Mpipi-Recharged model, like other residue-resolution models, does not explicitly capture this dynamic charge regulation.

By implicitly treating water and ions, Mpipi-Recharged does not explicitly account for the entropic changes associated with the reorganization of these species. However, the model indirectly incorporates some entropy-driven effects of water and ions through its asymmetric pair-specific Yukawa potential. By weakening the repulsion among like-charged pairs, the model accounts indirectly for the translational entropy of ions and water, which favors their localization near like-charged residues to reduce repulsion. Similarly, the stronger attraction between oppositely charged residues reflects the entropic gain achieved when counterions and water molecules are released from charged residues as they come into close proximity.

Explicit-solvent models, including MARTINI,^68,141,142^ SPICA,^143^ and HPSx-mW,^66^ have been developed to address the limitations of implicit-solvent models. These models have been successfully applied to study solvation effects in FUS-LCD condensates,^66,68,69^ conformational changes of multidomain proteins under different solute conditions,^141,142^ and the stability of poliovirus protein capsids in solution.^143,144^ However, explicit-solvent models are computationally expensive and suffer from slower dynamics, which limit their applicability to large-scale systems such as multicomponent condensates composed of hundreds of proteins and nucleic acids.^102,145^

A promising strategy to overcome these challenges involves optimizing the interaction potentials in implicit-solvent coarse-grained models using machine learning algorithms to approximate solvation free energies.^146−148^ This approach ensures physicochemical realism without compromising computational efficiency. Additionally, advancements such as improved parameter optimization and the introduction of more versatile potential expressions, as demonstrated in this study, enhance prediction quality, achieve near-quantitative accuracy, and ensure computational feasibility.

The Mpipi-Recharged model also incorporates a simple one-bead-per-nucleotide representation for single-stranded disordered RNA, parametrized specifically for polyU–polyU and polyU–protein interactions. However, this approach does not account for canonical Watson–Crick base pairing,^149,150^ noncanonical secondary structures (e.g., G–U wobble pairs),^151^ or phase transitions of pure RNA into liquid-like^152,153^ or solid-like condensed phases under specific salt conditions.^150,154^ Furthermore, Mpipi-Recharged oversimplifies RNA backbone flexibility, which can significantly influence phase behavior. This has been addressed by one-bead per nucleotide RNA models that merge specialized configurational potentials (including bond, angles and dihedral terms) with multibody forces but for pure RNA condensates.

Like other residue-resolution models,^60,155^ Mpipi-Recharged is inherently limited in capturing features beyond nonspecific RNA–protein interactions.^78,86^ Capturing specific RNA–protein binding often requires specialized potentials, such as multibody forces, which come at the cost of significantly increased computational expense.

Higher-resolution RNA models, such as those using three beads per nucleotide with implicit solvent,^156^ provide better representation of RNA structural features but have higher computational costs and limited compatibility with standard residue-resolution implicit-solvent protein models. Similarly, high-resolution explicit-solvent RNA models, such as the MARTINI RNA model,^157^ successfully reproduce stable RNA structures by representing nucleotides with multiple interacting sites. However, these models are computationally prohibitive for simulating large condensates containing tens of RNA strands and hundreds of nucleotides, which are feasible to simulate with Mpipi-Recharged (e.g., Figure 5d).

Despite its limitations, the Mpipi-Recharged model strikes a practical balance between accuracy and efficiency. Its computational efficiency allows it to investigate the phase behavior of highly charged protein or RNA–protein condensates, which are challenging for higher-resolution or explicit-solvent models. While future efforts are needed to address its constraints, Mpipi-Recharged offers a realistic and computationally feasible description of the experimental phase behavior of highly charged condensates, making it a valuable tool.

## Conclusions

In this work we introduce the Mpipi-Recharged model, a residue/nucleotide-resolution coarse-grained model for disordered proteins, multidomain proteins, and mixtures of proteins and single-stranded disordered RNAs. Mpipi-Recharged builds on the success of our original Mpipi model,^24^ by improving the description of charge–charge interactions, which are crucial to characterize the behavior of highly charged condensates. The Mpipi-Recharged model retains the key advantages of its predecessor: (a) employing the Wang–Frenkel potential^75^ to describe nonelectrostatic associative interactions, (b) deriving parameters from a combination of atomistic simulations of amino acid pairs and bioinformatics data, and (c) replacing the commonly used Lorentz–Berthelot combination rules with amino-acid-pair-specific parameters. These features collectively allow the Mpipi models to balance both π-based versus non-πinteractions, and arginine versus lysine associative interactions, yielding excellent predictions with experiments.^9,24,27^

A unique feature of the new Mpipi-Recharged model is that it abandons the symmetric description of charge–charge interactions (i.e., the use of a general Debye–Hückel potential for all charged amino acid pairs) in favor of an asymmetric pair-specific Yukawa potential. Such model feature was established based on our atomistic PMF curves of charged–charged amino acid pairs, which reveal that at mean-field level, there is a significant asymmetry in the strength of associative versus repulsive electrostatic interactions. Specifically, we observe a substantial reduction (up to −4 kcal/mol) in the free energy when amino acids with opposite charges bind to one another (i.e., R–E, R–D, K–E, and K–D) but negligible changes (less than +1 kcal/mol) when two amino acids of equal charges approach one another (i.e., R–R, K–K, E–E, and D–D). Notably, the decrease in the free energy of arginine upon binding to negatively charged amino acids is much lower than that of lysine, consistent with experimental observations^6,102^ and previous simulations.^6,24^

The striking contrast in the strength of interactions established by oppositely charged versus like-charged species is consistent with differences in the reorientation of amino acid chains as they come closer, changes in ion solvation, and variations in the translational entropy of ions and the solvent. Specifically, as the distance between amino acid pairs decreases, the side chains of oppositely charged amino acids, along with the solvating water molecules and surrounding ions, reorganize themselves to maximize (for oppositely charged pairs) electrostatic attraction or minimize (for like-charged pairs) repulsion.

We validate the new Mpipi-Recharge model against experimental single-molecule radii of gyration of approximately 50 IDRs,^9,110−121^ achieving near-quantitative agreement in all cases (Figure S2). Moreover, the model accurately predicts the experimental phase diagrams of hnRNPA1-A-LCD variants *in vitro*(9) (Figure 2b), similar to the original Mpipi model.^24^

Addressing the impact of charge blockiness on biomolecular phase behavior is perhaps one of the most challenging tasks for a coarse-grained model with implicit solvent and ions. These models inherently fail to account for how the distribution of charges within a protein dictates the organization and dynamics of ions surrounding such protein, the correlations between these ions, the screening of biomolecular charges by these ions, and the entropic gains from ion release during biomolecular interactions. Here we show that models like Mpipi-Recharged can alleviate some of these limitations through appropriate parametrization, thus partially compensating for the approximation of physical details, such as the absence of explicit solvent and ion representation. Specifically, we show that the asymmetric coarse-graining of electrostatic forces in Mpipi-Recharged results in excellent agreement with experiments probing the impact of charge blockiness on the stability of DDX4 and the R12 proteins (Figure 3). An additional stringent test, which we show Mpipi-Recharged excels at, is quantitatively recapitulating the salt-vs-concentration phase diagram of the H1–ProTα complex coacervate (Figure 4d). As in the case of charge blockiness, capturing the complex coacervation of H1–ProTα with a coarse-grained model is challenging due to the importance of correctly balancing electrostatic attraction versus repulsion.

Finally, we demonstrate that the Mpipi-Recharged model accurately describes the experimental RNA stoichiometric tresholds at which various protein–RNA condensates exhibit the different regimes of their reentrant phase behavior.^47−49,51^ That is, the model correctly predicts the polyU concentration that maximizes condensate stability for systems including FUS, or short synthetic peptides like RP3 or SR8 with polyU, and the concentration beyond which phase separation is hindered. For RNA-binding proteins such as TAF15, EWSR1, or hnRNPA1, the model matches the range of polyU concentrations that promote and subsequently hinder phase separation^51^ (Figure 5).

The Mpipi-Recharged model exploits the flexibility of both Wang–Frenkel and Yukawa potentials to include pair-specific parameters to approximate intermolecular interactions based on the specific chemical makeup of interacting species rather than the absolute charge of each amino acid or the mean hydrophobicity of the pair. This approach enhances the computational tools available for exploring the connection between phase transitions in biomolecular solutions and the physicochemical properties of the constituent biomolecules. Overall, the Mpipi-Recharged model demonstrates how, by compensating for their inherent lack of physical detail with carefully designed energy functions and parametrizations, approximate coarse-grained models can achieve excellent agreement with experimental results. Additionally, it complements experimental studies by offering mechanistic physicochemical insights into biomolecular phase behavior.

## Methods

### Mpipi-Recharged Model for Disordered, Globular, and Multidomain Proteins

Our residue-level coarse-grained model is built based on our original Mpipi model for biomolecular phase separation.^155^ Proteins are coarse-grained at amino acid resolution; i.e., each amino acid is represented with a single bead centered on its C_α_ atom. The mass of the bead corresponds to the total mass of the amino acid and its molecular diameter (σ) is estimated by assuming that the amino acid has a spherical shape with volume equal to its van der Waals volume.^158^ Beads that represent amino acids within IDRs/IDPs are connected by stiff harmonic bonds that maintain the mean *C*_α_–*C*_α_ distance observed experimentally in the backbones of proteins.^159^ Globular domains are treated as rigid bodies with the positions of all their amino acid beads being fixed relative to each other using an atomistic structure (e.g., AlphaFold prediction or crystal structure from the Protein Data Bank) as the reference (see Supporting Information for further details).

The total energy of a protein or a protein solution in the Mpipi-Recharged model is the sum of pairwise bonded (*E*_Bonded_) and nonbonded (*E*_Nonbonded_) potentials:
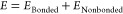
1The bonded potential is implemented to mantain amino acids in the protein backbone and is implemented for consecutive amino acids within a protein sequence that belong to the same IDR. However, IDRs are modeled as fully flexible polymers; thus, no energetic penalty for bending or torsion is considered. For bonds between an IDR and a globular domain in a multidomain protein, we also use the same bonded potential. The bonded potential is given by
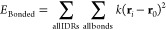
2where the equilibrium *C*_α_–*C*_α_ bond length is *r*_0_ = 3.81 Å, as suggested experimentally,^159^ and the spring constant, *k* = 9.6 kcal/(molÅ^2^), is set sufficiently high to ensure that the equilibrium bond lengths are preserved. The first sum iterates over all IDRs in the system and the second sum iterates over all bonds in each protein, as done before.^24^

The nonbonded potential is calculated for all pair of amino acids *ij*, excluding 1–2 consecutive interactions along the sequence. This nonbonded potential consist of the sum of a nonelectrostatic Wang–Frenkel potential (*E*_WF_) and an electrostatic Yukawa potential (*E*_Y_), as shown in eq 1.
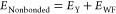
3The nonelectrostatic Wang–Frenkel potential^75^ (*E*_WF_) is given by
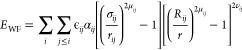
4where
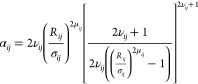
5Here σ_*ij*_ is the molecular diameter of the pair, defined from the individual molecular diameter of the individual amino acids (σ_*i*_ and σ_*j*_) using the Lorentz–Berthelot mixing rules: ). This pair molecular diameter, along with the pair-specific cutoff, *R*_*ij*_ = 3σ_*ij*_, define the pairwise distance at which the potential changes from repulsive (*r*_*ij*_ < σ_*ij*_) to attractive (σ_*ij*_>*r*_*ij*_> *R*_*ij*_). As in the original Mpipi model, the depth of the attractive well, – ϵ_*ij*_, is set specifically for each pair based on our atomistic PMF curves and bioinformatics data,^89^ rather than defined using Lorentz–Berthelot combination rules. All values of ϵ_*ij*_, σ_*ij*_, ν_*ij*_, and μ_*ij*_ are given in Table S4. Abandoning combination rules provides the Mpipi model with unique flexibility in its parametrization, allowing it to correctly balance the much stronger contributions of π-based versus non-π-based associations. For example, unlike what would be possible with Lorentz–Berthelot combination rules, pair-specific parameters can achieve a much larger value of ϵ_*ij*_ for both arginine–tyrosine and tyrosine–tyrosine interactions, while keeping that of arginine–arginine sufficiently low to consistently match the atomistic PMFs for the three pairs.

The exponents μ_*ij*_ and ν_*ij*_ are positive integer numbers that modulate the shape of the Wang–Frenkel potential. Specifically, higher values of μ_*ij*_ result in a steeper increase in the repulsive part of the potential energy as the distance decreases below σ_*ij*_. As in our previous model, we have set ν_*ij*_ = 1 for all pairs which ensures an efficient computational balance (see discussion in^75^). However, unlike the original Mpipi model, where μ_*ij*_ = 2 for most pairs, we have used higher values of μ_*ij*_ ranging from 2 to 12. Consequently, the average value of μ_*ij*_ in the Mpipi-Recharged and Mpipi models are ⟨μ_*ij*_⟩ = 3.45 and ⟨μ_*ij*_⟩ = 2.05, respectively. Using higher values of μ_*ij*_ is crucial to avoid interpenetration of beads when values of ϵ_*ij*_ are low.

One of the most notable changes in the Mpipi-Recharged model is the description of electrostatic interactions. In the Mpipi-Recharged model, these interactions are described with the Yukawa potential^160^ instead of the Debye–Hückel potential^108^ used originally. The Yukawa potential, given below, is a more general and flexible form of the screened Coulomb potential, given that the strength of interactions can be defined in a pair-specific manner simply by controlling the value of the parameter *A*_*ij*_:
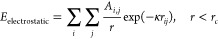
6Here, κ defines the screening length from the concentration of monovalent counterions in solution (*c*_*s*_), according to the expression , where *B*(ϵ_*r*_) = *e*^2^/4*π k*_*B*_*Tϵ*_0_ϵ_*r*_. The dielectric constant varies with temperature according to the following empiric formula:^161^
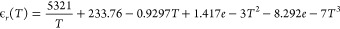
7

As mentioned earlier, the parameters *A*_*ij*_ of the Yukawa potential ((6)) are determined in a pair-specific manner informed by our atomistic PMF curves (Table S3). These atomistic simulations (Figure 1) suggest that, at the coarse-grained level, the relative strengths of electrostatic attraction and repulsion among charged amino acid pairs should vary considerably. Therefore, in the Mpipi-Recharged model, we set the Yukawa parameters describing interactions between oppositely charged pairs to be significantly stronger than those among pairs with the same charge. As demonstrated in the Results section of this work, this crucial consideration enables the Mpipi-Recharged model to more accurately capture the effects of charge–charge interactions in biomolecular phase separation. Moreover, it outperforms its predecessors for highly charged systems, while maintaining a low computational cost by avoiding the explicit description of water and ions. For the model parameter values of *A*_*ij*_, we can estimate the effective charge of each residue–residue pair in a Coulombic discrete charge scheme. By comparing the Yukawa potential with a Coulombic-like potential we obtain that
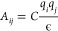
8where *C* is an energy-conversion constant, ϵ = 80 is the relative dielectric constant of water, and *q*_*i*_ and *q*_*j*_ are the charges of the *i*th and *j*th residues, respectively. The values of *A*_*ij*_ provided in Table S3 are expressed in kcal mol^–1^ Å, so that *C* = 332.15 kcal mol^–1^*e*^–2^ Å. Therefore, the charge of R–R interaction results |*q*_*R*_*q*_*R*_| = 0.98 *e*^2^, while the charges for the R–E pair is |*q*_*R*_*q*_*E*_| = 1.19 *e*^2^. We provide the values of the charge product in Table S4.

The interaction energy between pairs of amino acids in the Mpipi-Recharged model is represented as the sum of a pair-specific Wang-Frenkel potential, which describes nonionic interactions, and a Yukawa potential, which accounts for screened ionic interactions. This design choice enables the model to decouple the contributions of nonionic and ionic forces to phase separation. However, this combination of potentials complicates the direct fitting the Yukawa potential parameters to the PMF curves of charged pairs. The PMF curves represent the overall interaction free energy for specific configurations, reflecting not just electrostatic contributions but also other forces, such as van der Waals and hydrophobic interactions. Since the relative contributions of these forces are not well-defined, this introduces an additional free parameter into the model. To address this, we use the PMF results as a reference to balance the relative contributions of electrostatic attraction and repulsion. Specifically, as we did for parameters of the Wang-Frenkel potential in the original Mpipi model before,^24^ we adjust the values of the prefactor *A*_*ij*_ by a suitable multiplicative factor so that the relative values of the integrals of the well depths of the PMF curves of residue pairs *i* and *j*—normalized by the value for R–D (the strongest interacting pair)—are reasonably approximated those of the full Mpipi-Recharged model (Figure S16).

### Model for Disordered Single-Stranded RNA Chains

To model disordered single-stranded RNA chains within the Mpipi-Recharged model, we use one bead per nucleotide centered on the phosphorus atom. The total energy for RNA molecules consists on the same bonded and nonbonded terms given in eq 1 above. Single-stranded RNA chains are considered as fully flexible polymers, using the stiff harmonic potential *E*_*Bonded*_ given above, with the same spring constant used for proteins but an equilibrium bond length of *r*_0_ = 5.0 Å, which is close to the phosphorus–phosphorus distance reported for RNA.^162^ As for proteins, nonbonded interactions are defined by the sum of a Wang–Frenkel and Yukawa potentials, with parameters given in Tables S3 and S5.

### Atomistic Umbrella Sampling Molecular Dynamics Simulations to Calculate Potential of Mean Force for Pairs of Amino Acids and Pairs of Amino Acids and Uridine

We perform atomistic umbrella sampling MD simulations to estimate the free energy of interactions among charged–charged amino acid pairs and amino acid–Uridine pairs at different values of their COM of distances. These simulations were conducted in explicit water and ions, at a salt concentration of 150 mM NaCl, with Na^+^ and Cl^–^ ions. We use the a99sb-disp/PARMBSC1/JC-SPC/E-ion/TIP4*P*/2005 force field combination.^95,96^

In these simulations, the heavy atoms are restricted in the two perpendicular directions to that of the reaction coordinate using a harmonic potential function with a force constant of 240 kcal/mol/nm^2^. In the case of the amino acid pairs, the N- and C-terminal ends were capped using acetyl and *N*-methyl groups, respectively. The amino acids in each pair are oriented and restrained so that their side chains face each other. Their arrangements are based on the most common configurations observed in protein structures, because these are expected to correspond to their strongest binding modes. In cases where the preferred interaction was ambiguous, multiple orientations have been tested to identify those yielding the strongest interaction.^6,18,24^ At the PMF minima for each amino acid pair configuration, the side chains exhibit the smallest molecular separation within the residue, resulting in side chain–side chain interactions dominating the behavior. However, the PMF also incorporates inter-residue contributions from the other groups in the amino acids (e.g., backbone to side chain), given the size of the residue relative to the interaction potential cutoff of 1.4 nm.The pairwise COM distance between the pair of amino acids is varied from around 0.5 to 2 nm with 0.05 nm interval, yielding 34 to 40 simulation windows. In each window, we restrain the COM distance using a force constant of 1500 kcal/mol/nm^2^.

The integration of MD is carried out with a 2 fs time step. The cutoff radius for both dispersive interactions and the real part of electrostatic interactions is 1.4 nm. We use particle mesh Ewald summations^163^ to deal with electrostatic interactions. All simulations are run at the constant pressure of p = 1 bar, using an anisotropic Parrinello–Rahman barostat^164^ with a relaxation time of 1 ps. To fix the temperature, we employ a velocity-rescale thermostat^165^ with a relaxation time of 0.5 ps. Each window is simulated for 10 ns and three independent runs for each system to ensure statistical robustness. To compute PMF curves, we perform the analysis on the final 9 ns of each simulation. The final PMF curves are derived using the Weighted Histogram Analysis Method,^166^ enhanced with Bayesian bootstrapping. The resulting PMF curves complement the ones we have estimated previously for the parametrization of our original Mpipi model.^24^

### Calculation of Temperature vs Density Phase Diagrams via Direct Coexistence Simulations

We perform direct coexistence simulations^84,167^ to compute the phase diagrams of various protein and protein/RNA solutions in the temperature-vs-concentration plane. These simulations utilize an elongated simulation box to simultaneously simulate the high-density and low-density phases, separated by an interface. The simulation box is a right rectangular prism, with the longer side perpendicular to the interfaces. Periodic boundary conditions are applied across all directions of the simulation box.

Optimising the dimensions of the box is crucial to minimizing finite size effects. When defining the simulation box, we adhere to the following guidelines:

1. Use at least 100 proteins per simulation box for single-component systems and 64 for two-component systems.
2. Avoid self-interactions through periodic boundary conditions by ensuring that the short sides of the box are each larger than at least 2*R*_*g*_, where *R*_*g*_ is the radius of gyration of the largest molecule in the mixture within the condensate.
3. Verify that the long dimension of the simulation box is sufficiently long so that the overall density of the biomolecular solution inside the box is approximately 0.1 g/cm^3^. Higher densities can understabilize the condensed phase and may result in a significant underestimation of the critical solution temperature.

Once the simulation setup has been defined, we prepare a preliminary condensed phase at a given temperature by performing an NpT simulation at 1 bar and T = 273 K. Then, we place this preliminary condensed phase in the elongated simulation box and run an NVT direct coexistence simulation at a given temperature. We use a Langevin thermostat with a relaxation time of 5 ps and a time step of 10 fs. We perform each simulation for a total of ∼1.5 μs and verify that equilibrium has been reached by monitoring both the behavior of the potential energy of the system and the density of the system as as a function of the long side of the simulation box. We then perform a statistical analysis on the last 0.5–1 μs of the simulation postequilibration. During the analysis, we unwrap the system coordinates on the short sides of the box and recenter the coordinates of the biomolecules on the COM of the condensed phase to calculate the average equilibrium density as a function of the simulation box. If two different densities are detected—i.e., a high density phase and a low density phase—, we average out the densities of the two phases excluding the fluctuations of the interfaces, and plot the value of the two densities (on the *x*-axis) vs the simulation temperature (*y*-axis). To construct the coexistence curve of the phase diagram, we vary the temperature and repeat this procedure until only a single homogeneous phase is detected (i.e., when we are above the upper critical solution temperature). For each phase diagram, we estimate the coexistence densities at about 10 different temperatures, reducing the temperature spacing as we get closer to the critical solution temperature (with a maximum grid of 5 K). We then evaluate the critical temperature (*T*_c_) and critical density (ρ_*c*_) using the law of critical exponents and rectilinear diameters^109^ making sure that for the fit we use only the 3–4 temperature-vs-densities points closer to the critical region that still show sharp interfaces:
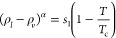
9
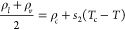
10where ρ_*l*_ and ρ_*v*_ refer to the densities of the condensed and the diluted phases respectively, *s*_1_ and *s*_2_ are fitting parameters, and α = 3.06 accounts for the critical exponent of the three-dimensional Ising model.^109^ All calculations are carried out in LAMMPS.^168^ Production runs are typically of the order of 1–2 μs depending on the components of the system and the molecular weight of the protein sequence.

### Estimation of the Critical Temperature via NpT Simulations

To calculate the critical temperature of multicomponent mixtures at precise stoichiometries, we run simulations using a cubic homogeneous box of protein/RNA mixtures in the NpT ensemble. This method avoids RNA migration to the diluted phase while ensuring an accurate estimation of the critical temperature.^85^ The initial configuration is arranged so that the RNA strands are homogeneously distributed and the density is near bulk conditions ρ_*box*_ ∼ 0.3 g/cm^3^. The temperature and pressure were kept constant using a Nosé–Hoover thermostat^169^ at T = 300 K (with 5 ps relaxation time) and a Parrinello–Rahman^164^ isotropic barostat at p = 0 bar (with a 5 ps relaxation time), respectively, and a time step of 10 fs. The critical temperature is calculated running simulations at different temperatures and taking the average of the highest phase separating temperature (with ρ_*box*_ ≳ 0.2) and the lowest overcritical temperature (with ρ_*box*_ ≲ 0.1). All calculations are carried out in LAMMPS.^168^ We note that using this approach, only the coexisting line of the condensed phase can be evaluated, whereas with direct coexistence simulations both coexistence lines of the diluted and condensed phase can be measured. However, using the NpT ensemble we ensure precise stoichiometries within condensates for multicomponent mixtures.

### Calculations of Single-Molecule Radii of Gyration

Single-molecule radii of gyration (*R*_*g*_) are computed for the IDRs listed in the Supporting Information. One replica of each corresponding protein is simulated in a cubic box (with a box size of ∼20 nm) using NVT simulations at the corresponding temperature (see Table S1) for 1.5 μs and a simulation time step of 10 fs. We use a Langevin thermostat with a relaxation time of 5 ps. All calculations are carried out in LAMMPS.^168^

## Data Availability

The data that supports the findings of this study are available within the article and its Supporting Methods. The LAMMPS and GROMACS files of the residue-resolution models and all-atom simulations, respectively, as well as the scripts to run the Mpipi-Recharged are available in the GitHub database under the accession code: [GitHub]. We also include an implementation of the Mpipi-Recharged model in OpenMM in the following repository [OpenMM].

## Supporting Information Available

Detailed description of the different computational methods employed in the study, model parameters, sequences of the proteins, and additional figures supporting our results including calculations with different coarse-grained models (PDF)
Transparent Peer Review report available (PDF)
