# Supplementary material for: Chemically Informed Coarse-Graining of Electrostatic Forces in Charge-Rich Biomolecular Condensates
Source: ACS Cent Sci. 2025 Feb 11;11(2):302–21. doi: 10.1021/acscentsci.4c01617 (PMC11869137; doi:10.1021/acscentsci.4c01617)
Supplement: Supplementary file 1 — oc4c01617_si_001.pdf [file oc4c01617_si_001.pdf]

Chemically-informed coarse-graining of electrostatic forces in charge-rich biomolecular condensates (Supporting Information)

Andrés R. Tejedor

Yusuf Hamied Department of Chemistry, University of Cambridge, Lensfield Road, Cambridge CB2 1EW, UK and Department of Physical-Chemistry Universidad Complutense de Madrid Av. Complutense s/n, Madrid 28040, Spain

Anne Aguirre Gonzalez, M. Julia Maristany, Pin Yu Chew, and Kieran Russell

Yusuf Hamied Department of Chemistry, University of Cambridge, Lensfield Road, Cambridge CB2 1EW, UK

Jorge Ramirez

Department of Chemical Engineering, Universidad Politécnica de Madrid, José Gutiérrez Abascal 2, 28006, Madrid, Spain

Jorge R. Espinosa*

Department of Physical-Chemistry Universidad Complutense de Madrid Av. Complutense s/n, Madrid 28040, Spain and Maxwell Centre, Cavendish Laboratory, Department of physics, University of Cambridge, JJ Thomson Avenue, Cambridge CB3 0HE, United Kingdom

Rosana Collepardo-Guevara†

Yusuf Hamied Department of Chemistry, University of Cambridge, Lensfield Road, Cambridge CB2 1EW, UK and Department of Genetics University of Cambridge Cambridge CB2 3EH, UK

(Dated: January 31, 2025)

* jorgerene@ucm.es
† rc597@cam.ac.uk

SI. SEQUENCES AND PDBS OF THE STUDIED PROTEINS

A. Sequences for radii of gyration

Some sequences are such as Prot-α and some hnRNPA1 variants are provided later so we have also calculated their phase behaviour. However, we give all the experimental values of all the radii of gyration studied along with the corresponding reference in Table S1.

α-synuclein:
MDVFMKGLSK AKEGVVAAAE KTKQGVAEAA GKTKEGVLYV GSKTKEGVVH GVATVAEKTK EQVTNVGGAV
VTGVTAVAQK TVEGAGSIAA ATGFVKKDQL GKNEEGAPQE GILEDMPVDP DNEAYEMPSE EGYQDYEPEA

ACTR:
GTQNRPLLRN SLDDLVGPPS NLEGQSDERA LLDQLHTLLS NTDATGLEEI DRALGIPELV NQGQALEPKQ D

Ash1:
GASASSSPSP STPTKSGKMR SRSSSPVRPK AYTPSPRSPN YHRFALDSPP QSPRRSSNSS ITKKGSRRSS
GSSPTRHTTR VCV

FhuA:
ESAWGPAATI AARQSATGTK TDTPIQKVPQ SISVVTAEEM ALHQPKSVKE ALSYTPGVSV GTRGASNTYD
HLIIRGFAAE GQSQNNYLNG LKLQGNFYND AVIDPYMLER AEIMRGPVSV LYGKSSPGGL LNMVSKRPTT
EPLK

hNHE1cdt:
MVPAHKLDSP TMSRARIGSD PLAYEPKEDL PVITIDPASP QSPESVDLVN EELKGKVLGL SRDPAKVAEE
DEDDDGGIMM RSKETSSPGT DDVFTPAPSD SPSSQRIQRC LSDPGPHPEP GEGEPFFPKG Q

IBB:
GCTNENANTP AARLHRFKNK GKDSTEMRRR RIEVNVELRK AKKDDQMLKR RNVSSFPDDA TSPLQENRNN
QGTVNWSVDD IVKGINSSNV ENQLQAT

K10:
MQTAPVPMPD LKNVKSKIGS TENLKHQPGG GKVQIVYKPV DLSKVTSKCG SLGNIHHKPG GGQVEVKSEK
LDFKDRVQSK IGSLDNITHV PGGGNKKIET HKLTFRENAK AKTDHGAEIV YKSPVVSGDT SPRHLSNVSS
TGSIDMVDSP QLATLADEVS ASLAKQGL

K16:
MSSPGSPGTP GSRSRTPSLP TPPTREPKKV AVVRTPPKSP SSAKSRLQTA PVPMPDLKNV KSKIGSTENL
KHQPGGGKVQ IINKKLDLSN VQSKCGSKDN IKHVPGGGSV QIVYKPVDLS KVTSKCGSLG NIHHKPGGGQ
VEVKSEKLDF KDRVQSKIGS LDNITHVPGG GNKKIE

K17:
MSSPGSPGTP GSRSRTPSLP TPPTREPKKV AVVRTPPKSP SSAKSRLQTA PVPMPDLKNV KSKIGSTENL
KHQPGGGKVQ IVYKPVDLSK VTSKCGSLGN IHHKPGGGQV EVKSEKLDFK DRVQSKIGSL DNITHVPGGG
NKKIE

K18:
MQTAPVPMPD LKNVKSKIGS TENLKHQPGG GKVQIINKKL DLSNVQSKCG SKDNIKHVPG GGSVQIVYKP
VDLSKVTSKC GSLGNIHHKP GGGQVEVKSE KLDFKDRVQS KIGSLDNITH VPGGGNKKIE

K23:
MAEPRQEFEV MEDHAGTYGL GDRKDQGGYT MHQDQEGDTD AGLKAEEAGI GDTPSLEDEA AGHVTQARMV
SKSKDGTGSD DKKAKGADGK TKIATPRGAA PPGQKGQANA TRIPAKTPPA PKTPPSSGEP PKSGDRSGYS
SPGSPGTPGS RSRTPSLPTP PTREPKKVAV VRTPPKSPSS AKSRLKKIET HKLTFRENAK AKTDHGAEIV

YKSPVVSGDT SPRHLSNVSS TGSIDMVDSP QLATLADEVS ASLAKQGL

K25:
MAEPRQEFEV MEDHAGTYGL GDRKDQGGYT MHQDQEGDTD AGLKAEEAGI GDTPSLEDEA AGHVTQARMV
SKSKDGTGSD DKKAKGADGK TKIATPRGAA PPGQKGQANA TRIPAKTPPA PKTPPSSGEP PKSGDRSGYS
SPGSPGTPGS RSRTPSLPTP PTREPKKVAV VRTPPKSPSS AKSRL

K27:
MSSPGSPGTP GSRSRTPSLP TPPTREPKKV AVVRTPPKSP SSAKSRLQTA PVPMPDLKNV KSKIGSTENL
KHQPGGGKVQ IVYKPVDLSK VTSKCGSLGN IHHKPGGGQV EVKSEKLDFK DRVQSKIGSL DNITHVPGGG
NKKIETHKLT FRENAKAKTD HGAEIVY

K32:
MSSPGSPGTP GSRSRTPSLP TPPTREPKKV AVVRTPPKSP SSAKSRLQTA PVPMPDLKNV KSKIGSTENL
KHQPGGGKVQ IINKKLDLSN VQSKCGSKDN IKHVPGGGSV QIVYKPVDLS KVTSKCGSLG NIHHKPGGGQ
VEVKSEKLDF KDRVQSKIGS LDNITHVPGG GNKKIETHKL TFRENAKAKT DHGAEIVY

K44:
MAEPRQEFEV MEDHAGTYGL GDRKDQGGYT MHQDQEGDTD AGLKAEEAGI GDTPSLEDEA AGHVTQARMV
SKSKDGTGSD DKKAKGADGK TKIATPRGAA PPGQKGQANA TRIPAKTPPA PKTPPSSGEP PKSGDRSGYS
SPGSPGTPGS RSRTPSLPTP PTREPKKVAV VRTPPKSPSS AKSRLQTAPV PMPDLKNVKS KIGSTENLKH
QPGGGKVQIV YKPVDLSKVT SKCGSLGNIH HKPGGGQVEV KSEKLDFKDR VQSKIGSLDN ITHVPGGGNK
KIE

N49:
GCQTSRGLFG NNNTNNINNS SSGMNNASAG LFGSKP

N98:
GCFNKSFGTP FGGGTGGFGT TSTFGQNTGF GTTSGGAFGT SAFGSSNNTG GLFGNSQTKP GGLFGTSSFS
QPATSTSTGF GFGTSTGTAN TLFGTASTGT SLFSSQNNAF AQNKPTGFGN FGTSTSSGGL FGTTNTTSNP
FGSTSGSLFG P

NLS:
ACETNKRKRE QISTDNEAKM QIQEEKSPKK KRKKRSSKAN KPPE

NSP:
GCNFNTPQQN KTPFSFGTAN NNSNTTNQNS STGAGAFGTG QSTFGFNNSA PNNTNNANSS ITPAFGSNNT
GNTAFGNSNP TSNVFGSNNS TTNTFGSNSA GTSLFGSSSA QQTKSNGTAG GNTFGSSSLF NNSTNSNTTK
PAFGGLNFGG GNNTTPSSTG NANTSNNLFG ATANAN

NUL:
GCGFKGFDTS SSSSNSAASS SFKFGVSSSS SGPSQTLTST GNFKFGDQGG FKIGVSSDSG SINPMSEGFK
FSKPIGDFKF GVSSESKPEE VKKDSKNDNF KFGLSSGLSN PV

NUS:
GCPSASPAFG ANQTPTFGQS QGASQPNPPG FGSISSSTAL FPTGSQPAPP TFGTVSSSSQ PPVFGQQPSQ
SAFGSGTTPN

P53:
MEEPQSDPSV EPPLSQETFS DLWKLLPENN VLSPLPSQAM DDLMLSPDDI EQWFTEDPGP DEAPRMPEAA
PPVAPAPAAP TPAAPAPAPS WPL

RNaseA:
VLLPLLVLVL LLVRVEPSLG KETAAAKFER QHIDSNPSSV SSSNYCNQMM KSRNLTQGRC KPVNTFVHES
LADVQAVCSQ KNVACKNGQT NCYQSYSTMS ITDCRETGSS KYPNCAYKTT QAKKHIIVAC EGNPYVPVHY
DASV

Sic1:
GSMTPSTPPR SRGTRYLAQP SGNTSSSALM QGQKTPQKPS QNLVPVTPST TKSFKNAPLL APPNSNMGMT
SPFNGLTSPQ RSPFPKSSVK RT

SH4-UD:
MGSNKSKPKD ASQRRRSLEP AENVHGAGGG AFPASQTPSK PASADGHRGP SAAFAPAAAE PKLFGGFNSS DTVTSPQRAG PLAGG

A1:
GSMASASSSQ RGRSGSGNFG GGRGGGFGGN DNFGRGGNFS GRGGFGGSRG GGGYGGSGDG YNGFGNDGSN FGGGGSYNDF GNYNNQSSNF GPMKGGNFGG RSSGGSGGGG QYFAKPRNQG GYGGSSSSSS YGSGRRF

A1-NLS:
GSMASASSSQ RGRSGSGNFG GGRGGGFGGN DNFGRGGNFS GRGGFGGSRG GGGYGGSGDG YNGFGNDGSN FGGGGSYNDF GNYNNQSSNF GPMKGGNFGG RSSGPYGGGG QYFAKPRNQG GYGGSSSSSS YGSGRRF

-8F+4Y:
GSMASASSSQ RGRSGSGNFG GGRGGGYGGN DNGGRGGNYS GRGGFGGSRG GGGYGGSGDG YNGGGNDGSN YGGGGSYNDS GNYNNQSSNF GPMKGGNYGG RSSGGSGGGG QYGAKPRNQG GYGGSSSSSS YGSGRRF

-9F+6Y:
GSMASASSSQ RGRSGSGNFG GGRGGGYGGN DNYGRGGNYS GRGGFGGSRG GGGYGGSGDG YNGGGNDGSN YGGGGSYNDS GNYNNQSSNF GPMKGGNYGG RSSGGSGGGG QYGAKPRNQG GYGGSSSSSS YGSGRRY

+2R:
GSMASASSSQ RGRSGSGNFG GGRGGGFGGN DNFGRGGNFS GRGGFGGSRG GGGYGGSGDG YNGFRNDGSN FGGGGRYNDF GNYNNQSSNF GPMKGGNFGG RSSGPYGGGG QYFAKPRNQG GYGGSSSSSS YGSGRRF

+7R:
GSMASASSSQ RGRSGRGNFG GGRGGGFGGN DNFGRGGNFS GRGGFGGSRG GGRYGGSGDR YNGFGNDGRN FGGGGSYNDF GNYNNQSSNF GPMKGGNFRG RSSGPYGRGG QYFAKPRNQG GYGGSSSSRS YGSGRRF

-10R+10K:
GSMASASSSQ KGKSGSGNFG GGKGGGFGGN DNFGKGGNFS GKGGFGGSKG GGGYGGSGDG YNGFGNDGSN FGGGGSYNDF GNYNNQSSNF GPMKGGNFGG KSSGGSGGGG QYFAKPKNQG GYGGSSSSSS YGSGKKF

B. hnRNPA1 variants

Below, we give the sequence of hnRNPA1-A-LCD (wild-type) and the different variants we employed.

wt:
MASASSSQRG RSGSGNFGGG RGGGFGGNDN FGRGGNFSGR GGFGGSRGGG GYGGSGDGYN GFGNDGSNFG GGGSYNDFGN YNNQSSNFGP MKGGNFGGRS SGPYGGGGQY FAKPRNQGGY GGSSSSSSYG SGRRF

-3R+3K:
MASASSSQRG KSGSGNFGGG RGGGFGGNDN FGRGGNFSGR GGFGGSKGGG GYGGSGDGYN GFGNDGSNFG GGGSYNDFGN YNNQSSNFGP MKGGNFGGRS SGGSGGGGQY FAKPRNQGGY GGSSSSSSYG SGRKF

-4F-2Y:
MASASSSQRG RSGSGNSGGG RGGGFGGNDN FGRGGNSSGR GGFGGSRGGG GYGGSGDGYN GFGNDGSNSG GGGSSNDFGN YNNQSSNFGP MKGGNFGGRS SGGSGGGGQY SAKPRNQGGY GGSSSSSSSG SGRRF

-6R+6K:
MASASSSQKG KSGSGNFGGG RGGGFGGNDN FGKGGNFSGR GGFGGSKGGG GYGGSGDGYN GFGNDGSNFG GGGSYNDFGN YNNQSSNFGP MKGGNFGGKS SGGSGGGGQY FAKPRNQGGY GGSSSSSSYG SGRKF

+7F-7Y:
MASASSSQRG RSGSGNFGGG RGGGFGGNDN FGRGGNFSGR GGFGGSRGGG GFGGSGDGFN GFGNDGSNFG GGGSFNDFGN FNNQSSNFGP MKGGNFGGRS SGGSGGGGQF FAKPRNQGGF GGSSSSSSFG SGRRF

+7K+12D
MASADSSQRD RDDKGNFGDG RGGGFGGNDN FGRGGNFSDR GGFGGSRGDG KYGGDGDKYN GFGNDGKNFG
GGGSYNDFGN YNNQSSNFDP MKGGNFKDRS SGPYDKGGQY FAKPRNQGGY GGSSSSKSYG SDRRF

+7R+12D
MASADSSQRD RDDRGNFGDG RGGGFGGNDN FGRGGNFSDR GGFGGSRGDG RYGGDGDRYN GFGNDGRNFG
GGGSYNDFGN YNNQSSNFDP MKGGNFRDRS SGPYDRGGQY FAKPRNQGGY GGSSSSRSYG SDRRF

-9F+3Y
MASASSSQRG RSGSGNFGGG RGGGYGGNDN GGRGGNYSGR GGFGGSRGGG GYGGSGDGYN GGGNDGSNYG
GGGSYNDSGN GNNQSSNFGP MKGGNYGGRS SGGSGGGGQY GAKPRNQGGY GGSSSSSSYG SGRRS

-12F+12Y
MASASSSQRG RSGSGNYGGG RGGGYGGNDN YGRGGNYSGR GGYGGSRGGG GYGGSGDGYN GYGNDGSNYG
GGGSYNDYGN YNNQSSNYGP MKGGNYGGRS SGGSGGGGQY YAKPRNQGGY GGSSSSSSYG SGRRY

-12F+12Y
MASADSSQRD RDDSGNFGDG RGGGFGGNDN FGRGGNFSDR GGFGGSRGDG GYGGDGDGYN GFGNDGSNFG
GGGSYNDFGN YNNQSSNFDP MKGGNFGDRS SGPYDGGGQY FAKPRNQGGY GGSSSSSSYG SDRRF

+12D
MASADSSQRD RDDSGNFGDG RGGGFGGNDN FGRGGNFSDR GGFGGSRGDG GYGGDGDGYN GFGNDGSNFG
GGGSYNDFGN YNNQSSNFDP MKGGNFGDRS SGPYDGGGQY FAKPRNQGGY GGSSSSSSYG SDRRF

+12E
MASAESSQRE REESGNFGEG RGGGFGGNDN FGRGGNFSER GGFGGSRGEG GYGGEGDGYN GFGNDGSNFG
GGGSYNDFGN YNNQSSNFEP MKGGNFGERS SGPYEGGGQY FAKPRNQGGY GGSSSSSSYG SERRF

-4D
MASASSSQRG RSGSGNFGGG RGGGFGGNGN FGRGGNFSGR GGFGGSRGGG GYGGSGGGYN GFGNSGSNFG
GGGSYNGFGN YNNQSSNFGP MKGGNFGGRS SGPYGGGGQY FAKPRNQGGY GGSSSSSSYG SGRRF

+4D
MASASSSQRD RSGSGNFGGG RGGGFGGNDN FGRGGNFSGR GDFGGSRGGG GYGGSGDGYN GFGNDGSNFG
GGGSYNDFGN YNNQSSNFGP MKGGNFGGRS SDPYGGGGQY FAKPRNQGGY GGSSSSSSYD SGRRF

+8D
MASASSSQRD RSGSGNFGGG RDGGFGGNDN FGRGDNFSGR GDFGGSRDGG GYGGSGDGYN GFGNDGSNFG
GGGSYNDFGN YNNQSSNFGP MKGGNFGGRS SDPYGGGGQY FAKPRNQDGY GGSSSSSSYD SGRRF

-10R
MASASSSQGG SSGSGNFGGG GGGGFGGNDN FGGGGNFSGS GGFGGSGGGG GYGGSGDGYN GFGNDGSNFG
GGGSYNDFGN YNNQSSNFGP MKGGNFGGSS SGPYGGGGQY FAKPGNQGGY GGSSSSSSYG SGGGF

-6R
MASASSSQGG RSGSGNFGGG RGGGFGGNDN FGGGGNFSGS GGFGGSRGGG GYGGSGDGYN GFGNDGSNFG
GGGSYNDFGN YNNQSSNFGP MKGGNFGGSS SGPYGGGGQY FAKPGNQGGY GGSSSSSSYG SGGRF

C. Multi-domain proteins

Here, we provide the sequence and PDBs corresponding to the analysis of multi-domain proteins. We also include the sequence of FUS-LCD.

FUS:
MASNDYTQQA TQSYGAYPTQ PGQGYSQQSS QPYGQQSYSG YSQSTDTSGY GQSSYSSYGQ SQNTGYGTQS
TPQGYGSTGG YGSSQSSQSS YGQQSSYPGY GQQPAPSSTS GSYGSSSQSS SYGQPQSGSY SQQPSYGGQQ
QSYGQQQSYN PPQGYGQQNQ YNSSSGGGGG GGGGGNYGQD QSSMSSGGGS GGGYGNQDQS GGGGSGGYGQ
QDRGGRGRGG SGGGGGGGGG GYNRSSGGYE PRGRGGGRGG RGGMGGSDRG GFNKFGGPRD QGSRHDSEQD
NSDNNTIFVQ GLGENVTIES VADYFKQIGI IKTNKKTGQP MINLYTDRET GKLKGEATVS FDDPPSAKAA

IDWFDGKEFS GNPIKVSFAT RRADFNRGGG NGRGGRGRGG PMGRGGYGGG GSGGGGRGGF PSGGGGGGGQ
QRAGDWKCPN PTCENMNFSW RNECNQCKAP KPDGPGGGPG GSHMGGNYGD DRRGGRGGYD RGGYRGRGGD
RGGFRGGRGG GDRGGFGPGK MDSRGEHRQD RRERPY

FUS-LCD:
MASNDYTQQA TQSYGAYPTQ PGQGYSQQSS QPYGQQSYSG YSQSTDTSGY GQSSYSSYGQ SQNTGYGTQS
TPQGYGSTGG YGSSQSSQSS YGQQSSYPGY GQQPAPSSTS GSYGSSSQSS SYGQPQSGSY SQQPSYGGQQ
QSYGQQQSYN PPQGYGQQNQ YNS

hnRNPA1:
MSKSESPKEP EQLRKLFIGG LSFETTDESL RSHFEQWGTL TDCVVMRDPN TKRSRGFGFV TYATVEEVDA
AMNARPHKVD GRVVEPKRAV SREDSQRPGA HLTVKKIFVG GIKEDTEEHH LRDYFEQYGK IEVIEIMTDR
GSGKKRGFAF VTFDDHDSVD KIVIQKYHTV NGHNCEVRKA LSKQEMASAS SSQRGRSGSG NFGGGRGGGF
GGNDNFGRGG NFSGRGGFGG SRGGGGYGGS GDGYNGFGND GGYGGGGPGY SGGSRGYGSG GQGYGNQGSG
YGGSGSYDSY NNGGGGGFGG GSGSNFGGGG SYNDFGNYNN QSSNFGPMKG GNFGGRSSGP YGGGGQYFAK
PRNQGGYGGS SSSSSYGSGR RF

TDP-43:
MSEYIRVTED ENDEPIEIPS EDDGTVLLST VTAQFPGACG LRYRNPVSQC MRGVRLVEGI LHAPDAGWGN
LVYVVNYPKD NKRKMDETDA SSAVKVKRAV QKTSDLIVLG LPWKTTEQDL KEYFSTFGEV LMVQVKKDLK
TGHSKGFGFV RFTEYETQVK VMSQRHMIDG RWCDCKLPNS KQSQDEPLRS RKVFVGRCTE DMTEDELREF
FSQYGDVMDV FIPKPFRAFA FVTFADDQIA QSLCGEDLII KGISVHISNA EPKHNSNRQL ERSGRFGGNP
GGFGNQGGFG NSRGGGAGLG NNQGSNMGGG MNFGAFSINP AMMAAAQAAL QSSWGMMGML ASQQNQSGPS
GNNQNQGNMQ REPNQAFGSG NNSYSGSNSG AAIGWGSASN AGSGSGFNGG FGSSMDSKSS GWGM

HP1:
MGKKTKRTAD SSSSEDEEEY VVEKVLDRRV VKGQVEYLLK WKGFSEEHNT WEPEKNLDCP ELISEFMKKY
KKMKEGENNK PREKSESNKR KSNFSNSADD IKSKKKREQS NDIARGFERG LEPEKIIGAT DSCGDLMFLM
KWKDTDEADL VLAKEANVKC PQIVIAFYEE RLTWHAYPED AENKEKETAK S

The following Protein Data Bank (PDB) codes were used to build the globular structured domains of: FUS (residues from 285–371 (PDB code: 2LCW) and from 422–453 (PDB code: 6G99)), wt-TDP-43 (residues 2-38, 40-49 and 51-79 all included in the same PDB, (PDB code: 5MDI) and from residues 193-267 (PDB code: 1WF0)), h-TDP-43 (additionally to the structured domains of wt-TDP-43, this variant has an α–helical domain from residues 307-349 (PBD code: 2N2C)), hnRNPA1 (residues from 8-91 and 103-181 in the same PDB (PDB code: 1L3K)), and HP1α dimer (residues 18-75 (PDB code: 3FDT) and the CSD in residues 110-173 (PDB code: 3I3C)).

D. R12 variants

Here, we provide the R12 variants studied in this work and taken from [1].

Wild-type R12:
KTTKIACKSP QPDPVDTPAS TKQRPKRNLR KADVEEEFLA LRKRTPSAGK AMDTPKPAVS DEKNINTFVE
TPVQKLDLLG NLPGSKRQPQ TPKEKAEALE DLVGFKELFQ TP

Pm9:
KTTKIACKSP QPDPVDEPAS TKQRPKRNLR KADVEEEFLA LRKREPEAGK AMDEPKPAVS DEKNINEFVE
EPVQKLDLLG NLPGEKRQPQ EPKEKAEALE DLVGFKELFQ EP

CBm-1:
KTTKIACKSP QPDPVDTPEE EKERPKRELR KEDVEEEFLA LRKRTPSAGK AMDTPKPAVS DEKNINTFVE
TPVQKLDLLG NLPGSKRQPQ TPKEKAEALE DLVGFKELFQ TP

CBm-2:
KTTKIACKSP QPDPVDTPAS TQQQPQQNLQ QADVEEEFLA LRKRTPSAGK AMDTPKPAVS DEKNINTFVE
TPVQKLDLLG NLPGSKRQPQ TPKEKAEALE DLVGFKELFQ TP

CBm-3:
KTTKIACKSP QPDPVDTPAS TKQRPKRNLR KADVEEEFLA LRKRTPSEEK EVDTPKPEVE DEKEIETFVE TPVEKLDLLG NLPGSKRQPQ TPKEKAEALE DLVGFKELFQ TP

CBm-4:
KTTKIACKSP QPDPVDTPAS TKQRPKRNLR KADVEEEFLA LRKRTPSAGK AMDTPKPAVS DEKNINTFVE TPVQKLDLLE ELPESKREPE TPKEKEEELE DLVEFKELFE TP

E. H1 and ProTα

H1:
TENSTSAPAA KPKRAKASKK STDHPKYSDM IVAAIQAEKN RAGSSRQSIQ KYIKSHYKVG ENADSQIKLS IKRLVTTGVL KQTKGVGASG SFRLAKSDEP KKSVAFKKTK KEIKKVATPK KASKPKKAAS KAPTKKPKAT PVKKAKKKLA ATPKKAKKPK TVKAKPVKAS KPKKAKPVKP KAKSSAKRAG KKK

ProTα:
GPMSDAAVDT SSEITTKDLK EKKEVVEEAE NGRDAPANGN AENEENGEQE ADNEVDEEEE EGGEEEEEEE EGDGEEEDGD EDEEAESATG KRAAEDDEDD DVDTKKQKTD EDD

SII. URIDINE POTENTIAL MEAN FORCE CALCULATIONS.

We carried out potential mean force (PMF) calculations to assess the energetic interplay between Uridine and different amino acids significant for phase separation.

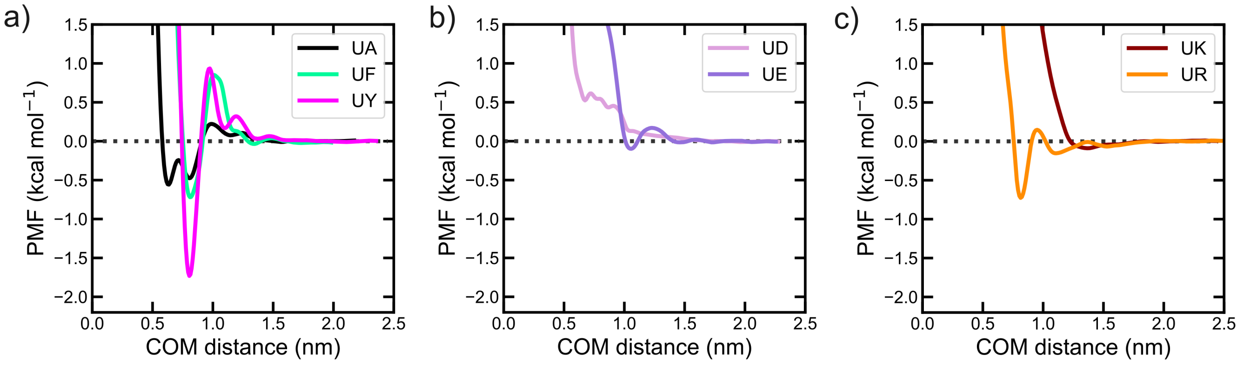

FIG. S1: Potential mean force calculations of Uridine nucleotide with different amino-acids including π (a), negative (b) and positive (c) residues.

SIII. FREE ENERGY INTERACTION BY INTEGRATION OF PMFS.

Here, we provide the value of the interaction considered as the minimum of the PMF curves to calculate the free-energy interaction in an alternative way. In Fig. S2 we show the resulting interaction energy normalised by the maximum value.

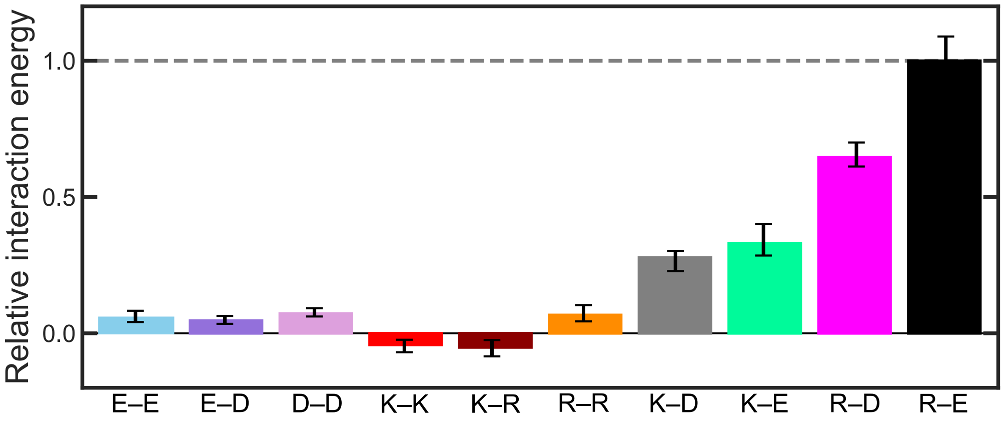

FIG. S2: Comparison of the free-energy of binding among charged amino acids relative to the R–D interaction. The values for the interaction are obtained from the minimum of the corresponding potential of mean force curve in panels **a-c** of Fig. 1 of the main. Error bars indicate the standard error extracted from the integral of the PMFs.

SIV. SINGLE PROTEIN RADII OF GYRATION.

The radii of gyration provided in Fig. S3 have been calculated as described in the Methods section of the manuscript. The parameter r indicates the linear coefficient parameters and D the deviation.

	R_g (nm)	C_{salt} (mM)	T (K)	Ref.
α-synuclein	3.31	185	293	[2]
ACTR	2.51	200	278	[3]
Ash1	2.85	150	298	[4]
FhuA	3.34	150	298	[5]
hNHE1cdt	3.63	200	278	[3]
IBB	3.12	162	298	[6]
K10	4.00	150	288	[7]
K16	3.90	150	288	[7]
K17	3.60	150	288	[7]
K18	3.80	150	288	[7]
K25	4.40	150	288	[7]
K23	4.90	150	288	[7]
K27	3.70	150	288	[7]
K32	4.20	150	288	[7]
K44	5.20	150	288	[7]
N49	1.59	162	298	[6]
N98	2.86	162	298	[6]
NLS	2.40	162	298	[6]
NSP	4.10	162	298	[6]
NUL	3.00	162	298	[6]
NUS	2.49	162	298	[6]
P53	2.87	108	298	[8]
ProTα	3.79	155	300	[9, 10]
RNaseA	3.36	150	298	[5]
SH4-UD	2.90	217	300	[11]
Sic1	3.21	162	298	[12]
A1	2.76	150	298	[13]
A1-NLS	2.58	150	298	[13]
-12F+12Y	2.60	150	298	[13]
+7F-7Y	2.72	150	298	[13]
-9F+6Y	2.66	150	298	[13]
-8F+4Y	2.71	150	298	[13]
-9F+6Y	2.68	150	298	[13]
-10R	2.67	150	298	[13]
-6R	2.57	150	298	[13]
+2R	2.62	150	298	[13]
+7R	2.71	150	298	[13]
-3R+3K	2.63	150	298	[13]
-6R+6K	2.79	150	298	[13]
10r10k	2.85	150	298	[13]
-4D	2.64	150	298	[13]
+4D	2.72	150	298	[13]
8D	2.69	150	298	[13]
+12D	2.80	150	298	[13]
+12E	2.85	150	298	[13]
+7K+12D	2.92	150	298	[13]

TABLE S1: Experimental single-protein radii of gyration including the salt concentration (C_{salt}), the temperature of the measure, and the reference.

SV. PARAMETRIZATION OF THE MPIPI-RECHARGED.

The Mpipi-Recharged uses PMF calculations as a framework to understand the attractive vs. repulsive electrostatic interaction balance finding an asymmetry in the results PMF

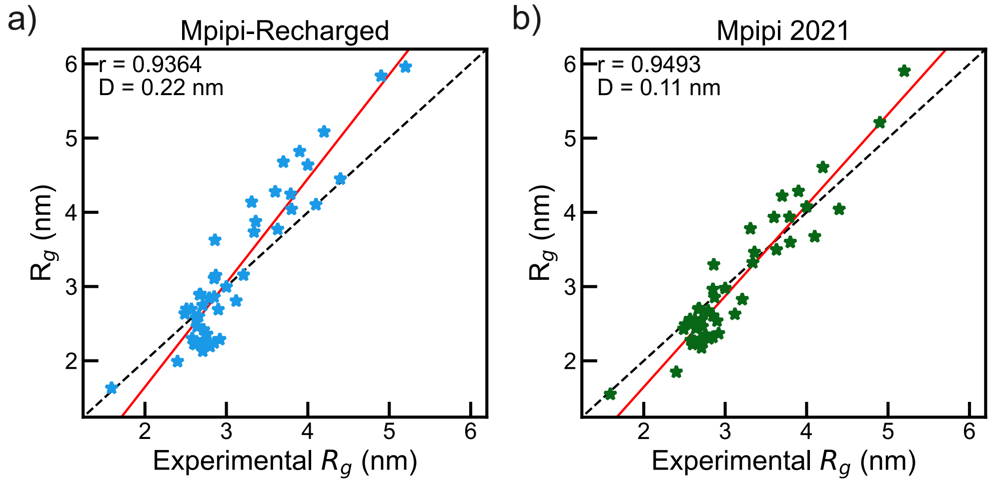

FIG. S3: Comparison of the simulated radii of gyration of single-protein using the Mpipi-Recharged (a) and the orginal Mpipi (Mpipi 2021 [14]) (b) with the corresponding experimental value.

curve (see Fig. 1 in the main text). However, our atomistic calculations are not directly implemented into our CG parameters, but they serve

SVI. HNRNPA1 VARIANTS CRITICAL TEMPERATURE

In Table S2 we show the critical temperature obtained from the experiments in [13]. Furthermore, we have calculated the phase diagram of the charged variants of hnRNPA1-LCD and the resulting critical temperature is shown in Fig. S5 for the original Mpipi model [14]. The resulting critical temperature is compared with the experimental saturation concentration obtained in the work of Bremer *et al.* [13].

Protein	Experimental T_c (K)
Wild-type	336
-12F+12Y	334
-3R+3K	309
-4F-2Y	300
-6R+6K	288
+7F-7Y	325
+7K+12D	333
+7R+12D	373
-9F+3Y	285

TABLE S2: Experimental critical temperature of the different hnRNPA1 variants studied, estimated from the phase diagrams reported by Bremer and co-workers [13].

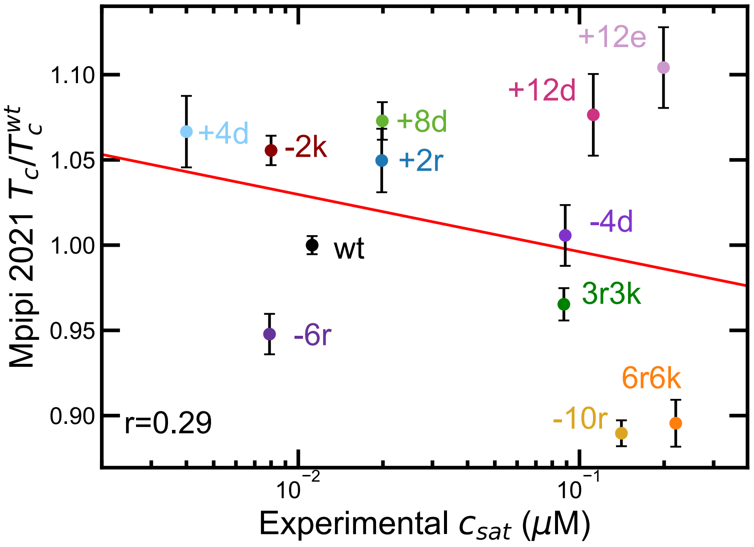

FIG. S4: Comparison of the simulated critical temperature using the original Mpipi [14] with the saturation concentration measured in experiments [13].

SVII. DDX4 PHASE DIAGRAMS USING MODELS WITH EXPLICIT CHARGES

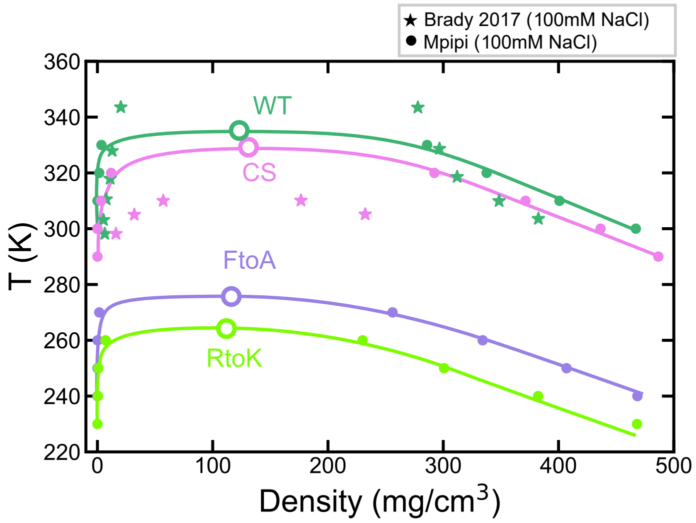

FIG. S5: Comparison of the simulated phase diagram using the original Mpipi [14] at [NaCl]=100mM with the experimental phase diagram calculated in the work of Brady *et al.* [15].

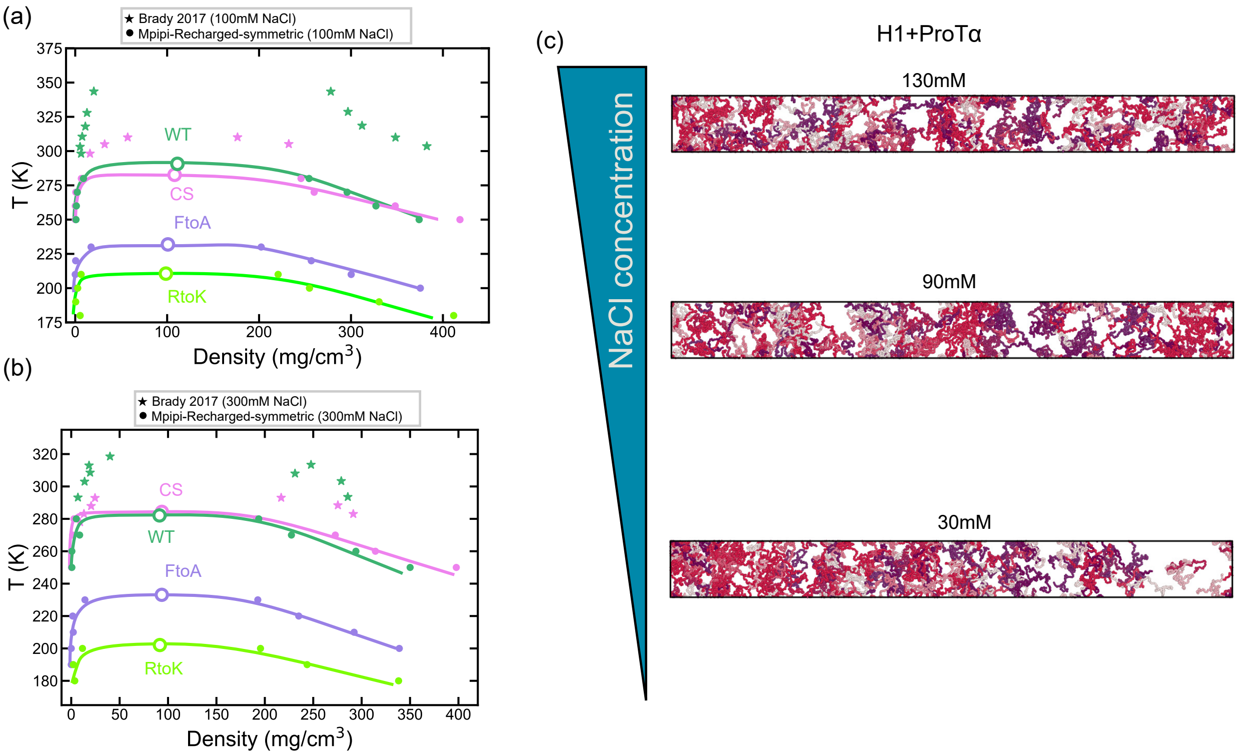

FIG. S6: Comparison of the simulated phase diagram using the Mpipi-Recharged with explicit charges of the original Mpipi [14] at [NaCl]=100mM (a) and [NaCl]=300mM (b) at with the experimental phase diagram calculated in the work of Brady *et al.* [15]. (c) H1+ProTα significant snapshots showing no phase separation at any salt concentration.

SVIII. GLOBULAR PROTEINS ANALYSIS

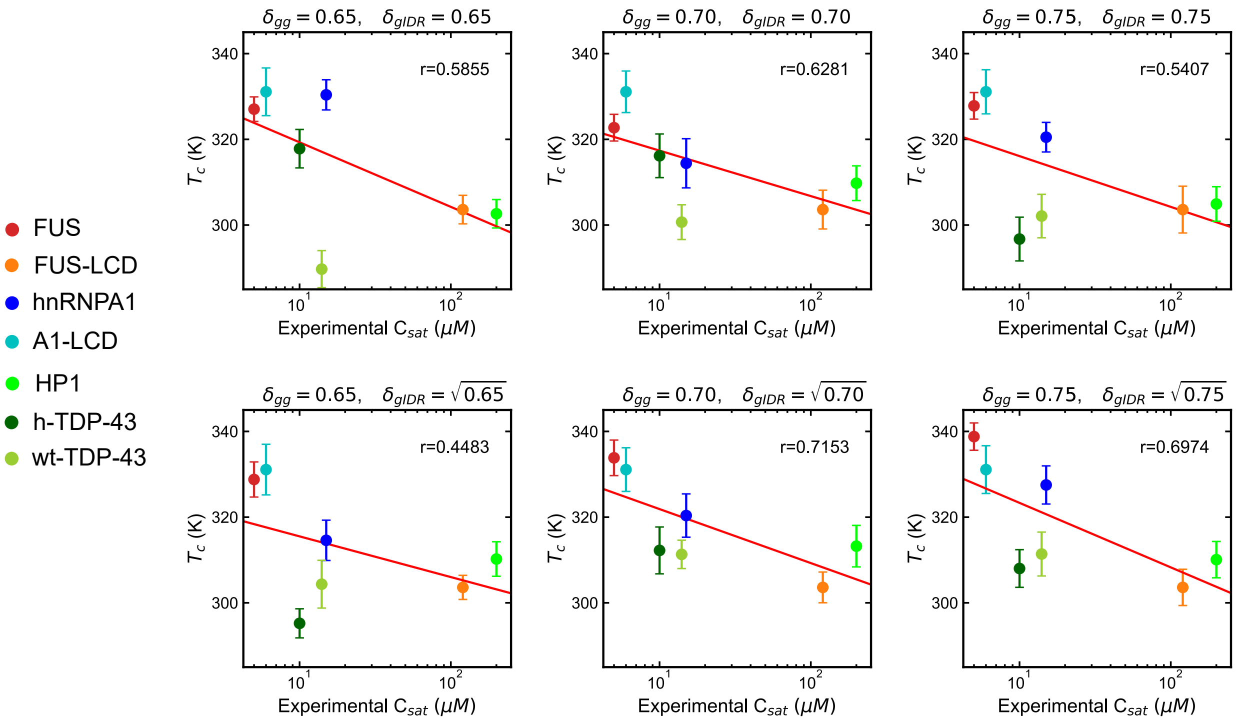

FIG. S7: Critical temperature of the studied multi-domain proteins from simulations using different parameters (as indicated in the top of each panel) vs. the experimental saturation concentration C_{sat} for FUS [16–18], FUS-LCD [18], hnRNPA1 [19], hnRNPA1-LCD [13, 20], HP1 [21], and TDP-43 [22, 23].

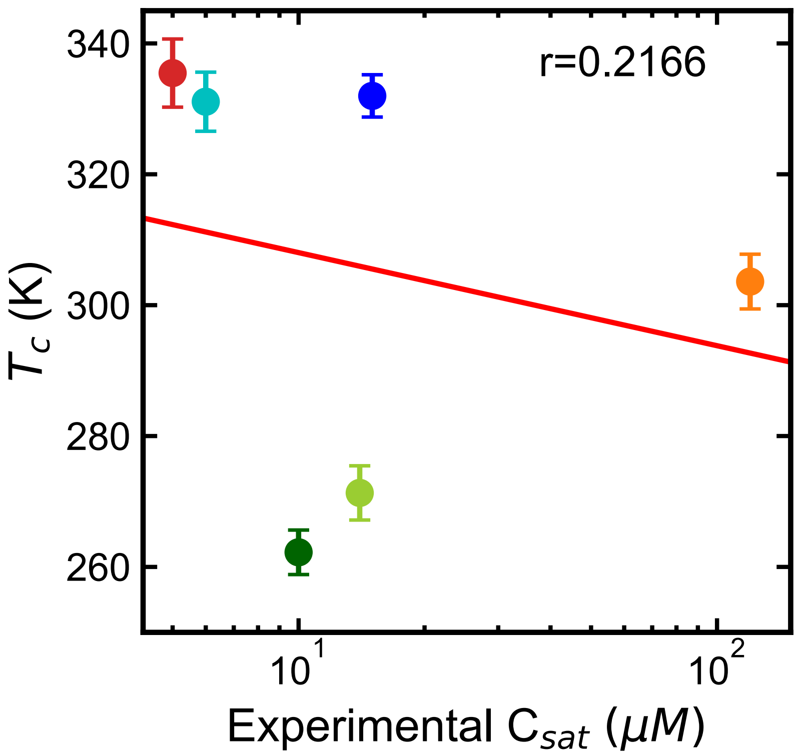

FIG. S8: Critical temperature of the studied multi-domain proteins from simulations using the position of the centre of mass vs. the experimental saturation concentration C_{sat} for FUS [16–18], FUS-LCD [18], hnRNPA1 [19], hnRNPA1-LCD [13, 20], and TDP-43 [22, 23]. The color code is the same as in the previous figure

SIX. MAPS OF CONTACTS

We calculated the H1-ProTα intermolecular contact maps from DC trajectories at a salt concentration of [NaCl]= 30mM and [NaCl]= 130mM. We used a σ-dependent cutoff that accounts for the specific nature of the residue, and it is set to $1.2\sigma_{ij}$, where σ_{ij} accounts for the mean excluded volume of the specific amino acids i^{th} and j^{th}. Here we provide all the contact maps between all the components of the H1+ProTα complex coacervate including the position of the charged residues (Fig. S7A-C) and without indicating the charged residues (Fig. S7D-F). The aspect ratio of all contact maps has been adapted to be square.

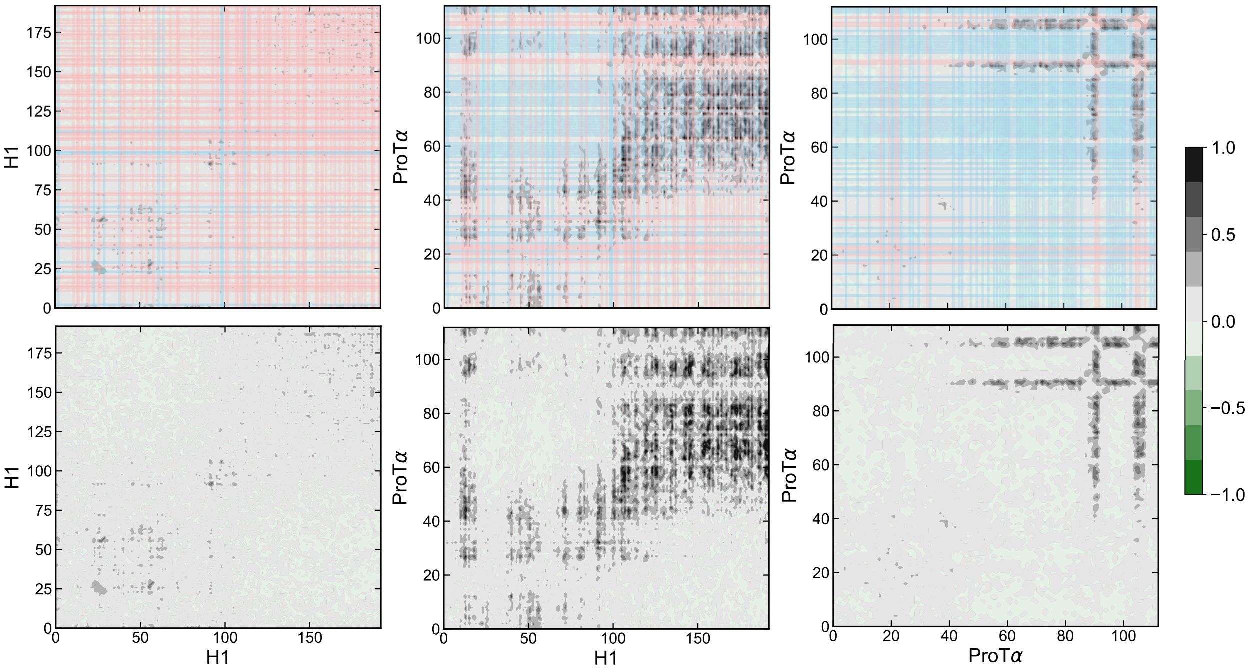

FIG. S9: Intermolecular contact frequency difference (in number of contacts per residue) between H1-H1, H1-ProTα, and ProTα-ProTα (as indicated in the axis labels) between the system at 30mM and 130mM of KCl concentration. Top panels include red lines to indicate the positively charged residues, and blue lines for the negatively charged residues.

SX. ELECTROSTATIC-DRIVEN PHASE SEPARATION USING MODELS WITH EXPLICIT CHARGES

Using the Mpipi-Recharged model but with explicit charges from the Mpipi, no phase separation is observed at any value of salt concentration.

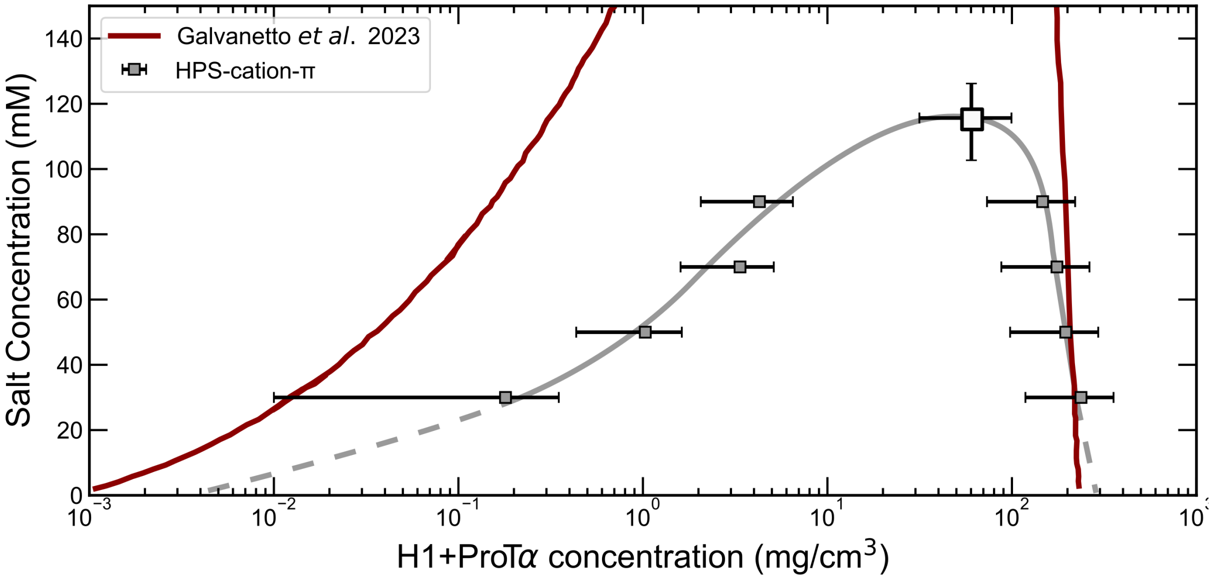

FIG. S10: Intermolecular contact frequency difference (in number of contacts per residue) between H1-H1, H1-ProTα, and ProTα-ProTα (as indicated in the axis labels) between the system at 30mM and 130mM of KCl concentration. Top panels include red lines to indicate the positively charged residues, and blue lines for the negatively charged residues.

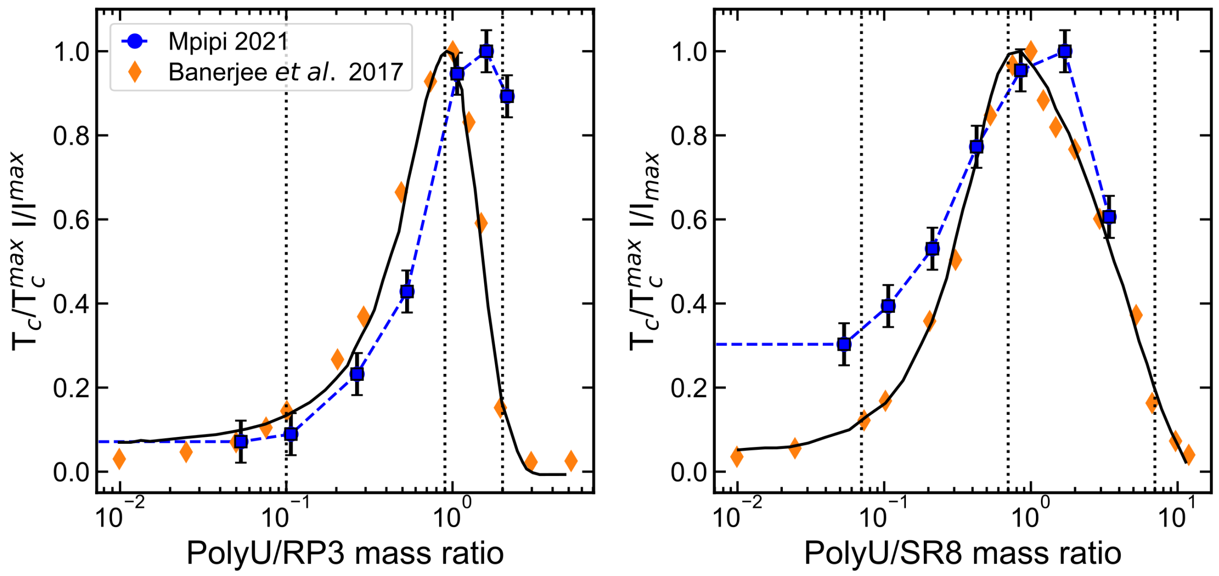

FIG. S11: Predictions of the RNA-driven reentrant phase behaviour of protein condensates by the Mpipi model [14]. Comparison of simulated critical temperature (blue symbols) with in vitro solution turbidity experiments [24] (yellow symbols) as a function of the polyU/peptides mass ratio for RP3 (**a**) and SR8 synthetic peptides (**b**). Both simulation critical temperatures (T_c) and fluorescence intensities (I) are normalised by the maximum value of the set.

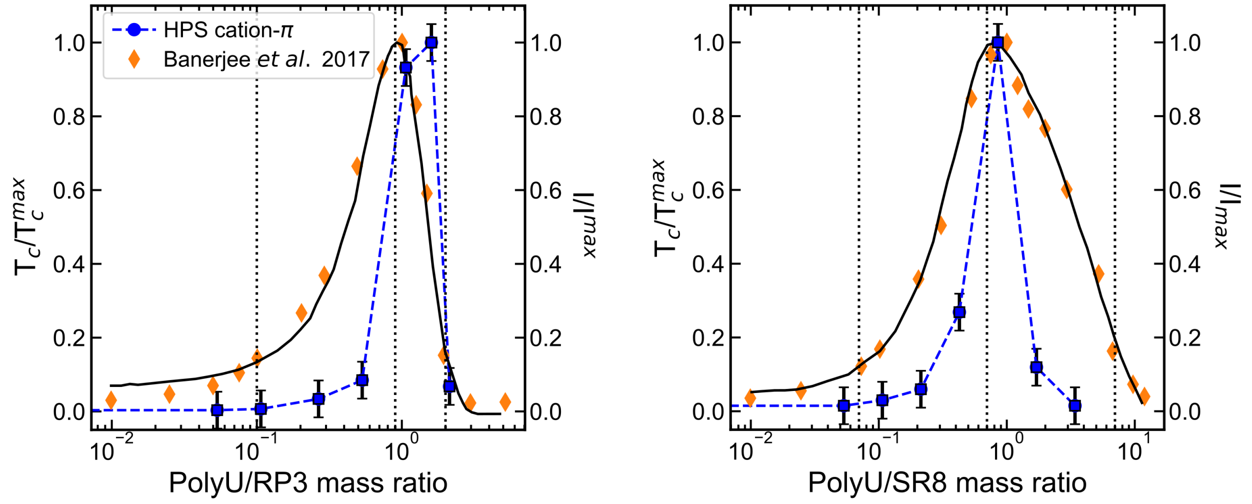

FIG. S12: Predictions of the RNA-driven reentrant phase behaviour of protein condensates by the HPS-cation-π model [14]. Comparison of simulated critical temperature (blue symbols) with in vitro solution turbidity experiments [24] (yellow symbols) as a function of the polyU/peptides mass ratio for RP3 (**a**) and SR8 synthetic peptides (**b**). Both simulation critical temperatures (T_c) and fluorescence intensities (I) are normalised by the maximum value of the set.

SXI. MPIPI-RECHARGED PHASE SEPARATION SUMMARY.

We plotted in Fig. S13 a summary figure that gathers all the simulated critical temperatures vs. the corresponding saturation concentration. The fit to the data gives a significant correlation coefficient of r=0.731 in a wide range of saturation concentrations. The red line represents a linear fit ($y = A\log_{10} x + B$) obtaining the coefficients $A = -53.5K$ and $B = 386K$.

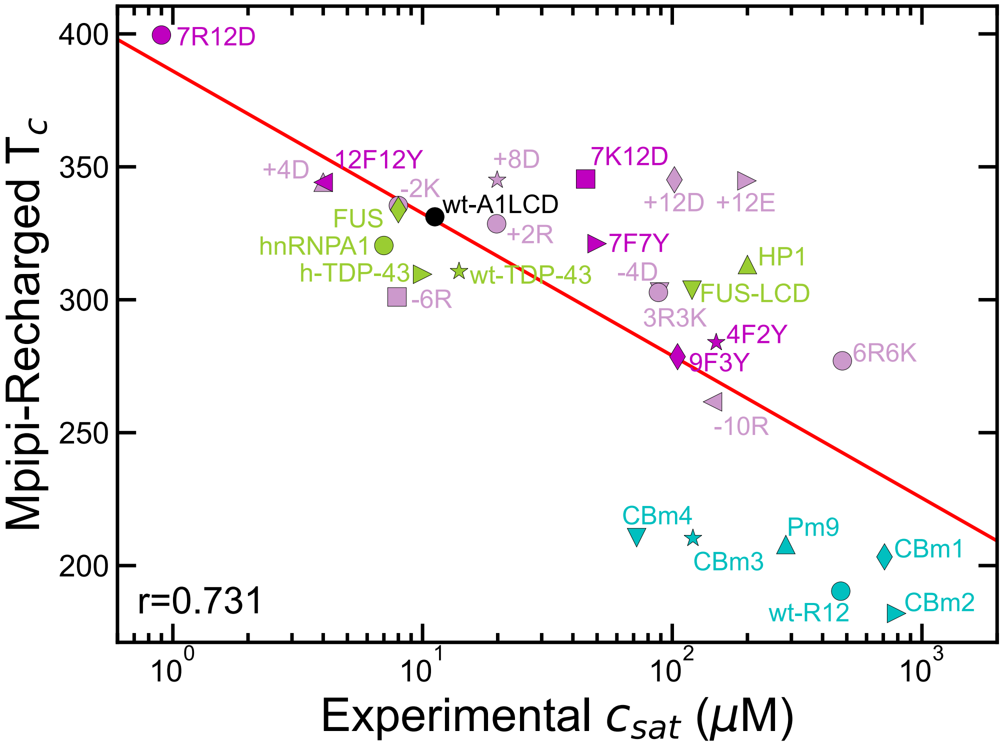

FIG. S13: Comparison of our simulated critical temperature with the experimental saturation concentration C_{sat} for all the variants simulated in this work. The colours group the systems in A1-LCD (black), A1-LCD charged variants (pink), other A1-LCD variants (magenta), multi-domain proteins (yellow-green), and R12 variants (cyan). Solid red line provide the fit to the data.

SXII. MPIPI-RECHARGED PARAMETERS

We have plotted in Fig. S14 the interaction strength normalized by the maximum interaction pair using only the Wang-Frenkel potential (right) and both the Wang-Frenkel and Yukawa contributions.

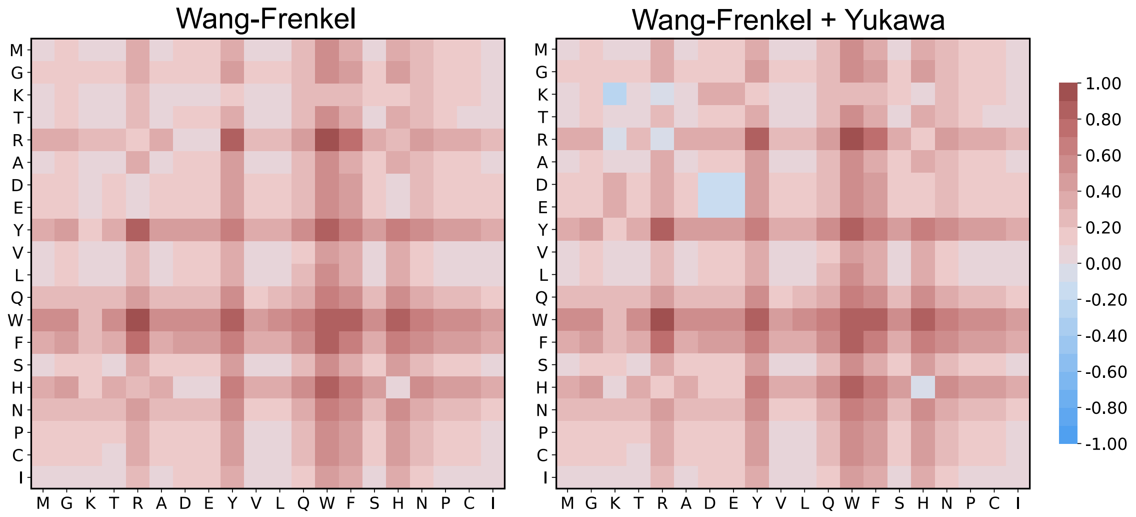

FIG. S14: Relative interaction strength values for the Mpipi-Recharged model considering the Wang-Frenkel (right panel) and both the Wang-Frenkel and Yukawa contributions. Values have been normalized by the highest interaction value.

In Table S3 we provide the parameters for the Yukawa interaction for all the charged amino acids. The interaction is given by the parameter A_{ij}. The analogous Coulomb potential is given by

$$E_{Coulomb} = \frac{k}{\epsilon}\frac{q_i q_j}{r}, \tag{S1}$$

where $k = 1/4\pi\varepsilon_0$ is the Coulombic constant, ϵ is the dielectric constant of the medium, and q_i, q_i are the charges of the interacting particles. Given this potential, the interaction parameter for the Yukawa potential is calculated as $A_{ij} = k\, q_i q_j/\epsilon$. For the values typically used in previous models, *i.e.* $k = 331.61$ kcal mol$^{-1}e^{-2}$ Å, $\epsilon = 80$, and $q_{i,j} = \pm 1e$ (or $q_H = +0.5e$ for histidine), the magnitude of the interaction is $|A_{ij}| = 4.145$ (or $|A_{iH}| = 2.073$ when involving histidine). The sign of A_{ij} depends on whether the interaction is attractive or repulsive.

	**R**	**K**	**H**	**D**	**E**
**R**	+4.00	+4.00	+1.47	-4.91	-4.93
**K**	+4.00	+4.00	+1.48	-4.33	-4.34
**H**	+1.47	+1.48	+1.04	-2.46	-2.45
**D**	-4.91	-4.33	-2.46	+4.00	+4.00
**E**	-4.93	-4.34	-2.45	+4.00	+4.00

TABLE S3: Parameter A_{ij} (in kcal mol^{-1} Å) for the Yukawa potential for each charged pair-residue.

The parameter A_{ij} can be directly converted into the product $q_i q_j$ to verify the actual value of the charges when considering a Coulomb-like potential.

	q_R	q_K	q_H	q_D	q_E
q_R	+0.963	+0.963	+0.354	-1.182	-1.187
q_K	+0.963	+0.963	+0.354	-1.043	-1.042
q_H	+0.354	+0.354	+0.25	-0.593	-0.592
q_D	-1.182	-1.043	-0.593	+0.963	+0.963
q_E	-1.187	-1.042	-0.592	+0.963	+0.963

TABLE S4: Charge product $q_i q_j$ equivalent to the paremeter A_{ij} for the Yukawa potential for each charged pair-residue.

We now compare the interaction of the Mpipi-Recharged with the calculated interactions from the PMF calculations. In Fig. S15

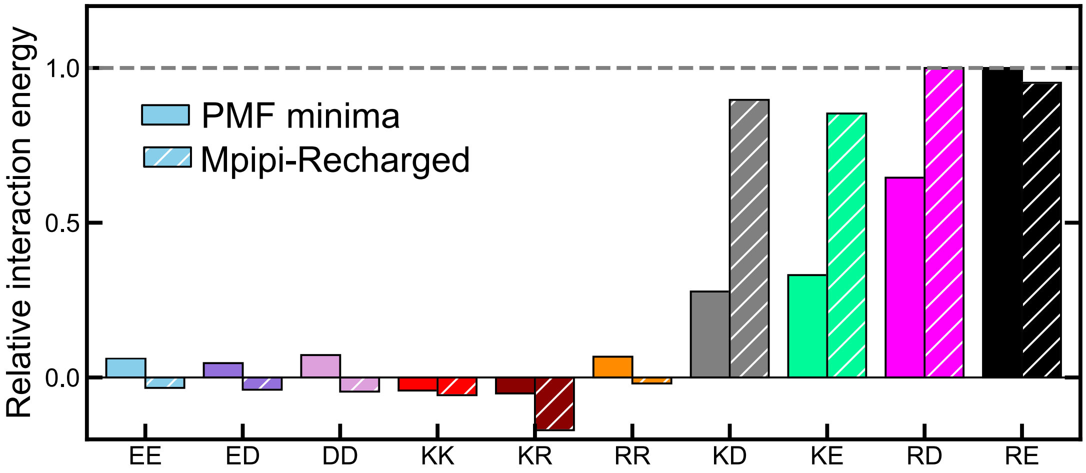

FIG. S15: Relative interaction strength values from the PMF calculations (solid bars) and for the Mpipi-Recharged model considering both the Wang-Frenkel and Yukawa contributions (dashed bars). Values have been normalized by the highest interaction value and the interactions from the PMFs are calculated with the minimum as presented in Fig. 1 in the main.

Finally, we test whether the attractive electrostatic interactions (e.g. KE, RE) are reasonably balanced contribute to condensate stability while a system containing only short polymers of charged residues remain dissolved. To prove this, we have run a simulation of polyK10 with polyD10 at 300K in a rectangular slab. The result is a homogeneous system with no hint of phase separation at all (Fig. S17).

Furthermore, we have analysed the most frequent pair residues interacting inside the H1-ProTα condensate. As can be seen in Fig. S18, the attractive electrostatic interactions are those sustaining the condensate by far, being KE and KD the most frequent interacting pairs.

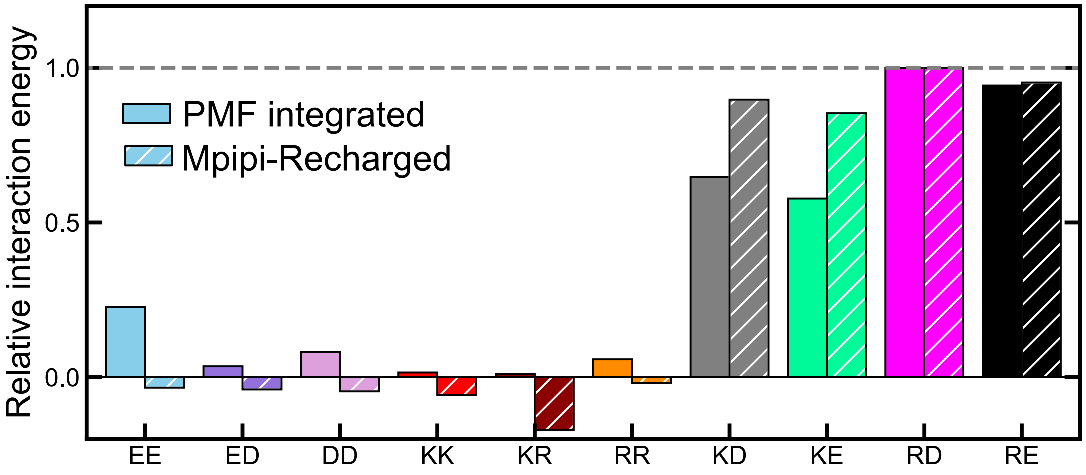

FIG. S16: Relative interaction strength values from the PMF calculations (solid bars) and for the Mpipi-Recharged model considering both the Wang-Frenkel and Yukawa contributions (dashed bars). Values have been normalized by the highest interaction value and the interactions from the integration of the attractive part of the PMF curves as presented in Fig. S2.

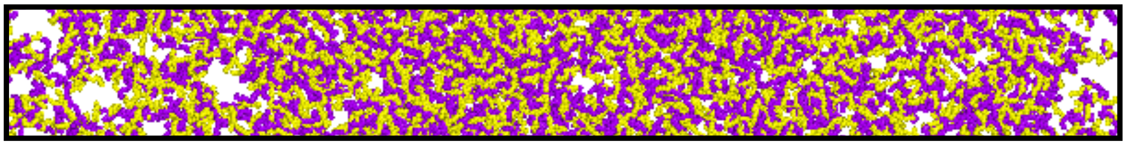

FIG. S17: Representative slab snapshot of the direct coexistence of polyK10 and polyD10 at 300K.

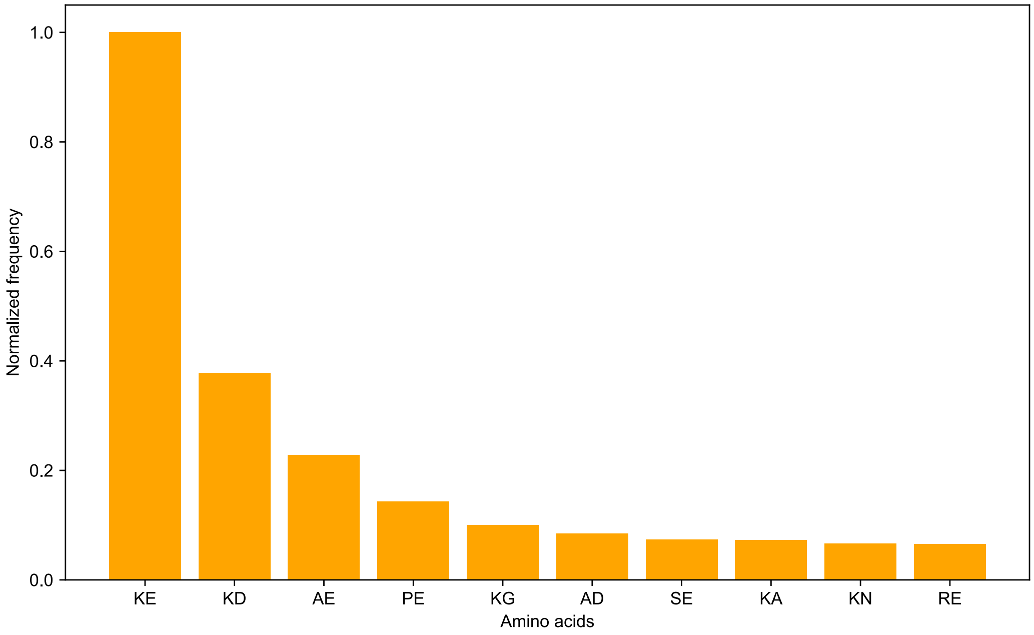

FIG. S18: Most frequent amino acid pair contacts from H1-ProTα direct coexistence at 130mM of NaCl.

Table of parameters:

Residue ith	Residue jth	ϵ (kcal mol^{-1})	σ (Å)	ν	μ
**A**	**A**	0.0912	5.2701	1	4
**A**	**D**	0.1363	5.5468	1	3
**A**	**E**	0.1437	5.7239	1	3
**A**	**Y**	0.3808	6.0019	1	3
**A**	**V**	0.0529	5.7680	1	4
**A**	**L**	0.0577	5.9021	1	4
**A**	**Q**	0.2226	5.7740	1	3
**A**	**W**	0.5005	6.1683	1	3
**A**	**F**	0.3582	5.9498	1	3
**A**	**S**	0.1017	5.3414	1	4
**A**	**H**	0.3680	5.8039	1	3
**A**	**N**	0.2169	5.5967	1	3
**A**	**P**	0.1166	5.5381	1	3
**A**	**C**	0.1084	5.4972	1	4
**A**	**I**	0.0484	6.0959	1	4
**C**	**C**	0.1257	5.7244	1	3
**C**	**I**	0.0657	6.3230	1	4
**D**	**D**	0.1379	5.8235	1	3
**D**	**E**	0.1434	6.0006	1	3
**D**	**Y**	0.4132	6.2786	1	3
**D**	**V**	0.0994	6.0448	1	4
**D**	**L**	0.1040	6.1788	1	4
**D**	**Q**	0.2632	6.0507	1	3
**D**	**W**	0.5289	6.4450	1	3
**D**	**F**	0.3914	6.2265	1	3
**D**	**S**	0.1465	5.6181	1	3
**D**	**H**	0.0080	6.0807	1	7
**D**	**N**	0.2576	5.8734	1	3
**D**	**P**	0.1608	5.8148	1	3
**D**	**C**	0.1530	5.7739	1	3
**D**	**I**	0.0951	6.3726	1	4
**E**	**E**	0.1489	6.1777	1	3
**E**	**Y**	0.4202	6.4557	1	3
**E**	**V**	0.1068	6.2218	1	4
**E**	**L**	0.1114	6.3559	1	4
**E**	**Q**	0.2706	6.2278	1	3
**E**	**W**	0.5360	6.6221	1	3
**E**	**F**	0.3984	6.4036	1	3
**E**	**S**	0.1539	5.7952	1	3
**E**	**H**	0.0080	6.2577	1	7
**E**	**N**	0.2650	6.0505	1	3
**E**	**P**	0.1682	5.9919	1	3
**E**	**C**	0.1604	5.9510	1	3
**E**	**I**	0.1025	6.5497	1	4
**F**	**F**	0.6004	6.6296	1	2
**F**	**S**	0.3681	6.0211	1	3
**F**	**H**	0.6198	6.4837	1	2
**F**	**N**	0.4770	6.2765	1	3
**F**	**P**	0.3822	6.2179	1	3
**F**	**C**	0.3745	6.1770	1	3
**F**	**I**	0.3178	6.7756	1	3
**G**	**A**	0.1321	4.9826	1	3
**G**	**C**	0.1493	5.2097	1	3
**G**	**D**	0.1758	5.2593	1	3
**G**	**E**	0.1832	5.4364	1	3
**G**	**F**	0.3968	5.6623	1	3
**G**	**G**	0.1730	4.6951	1	3

**G**	**H**	0.4089	5.5165	1	3
**G**	**I**	0.0893	5.8084	1	4
**G**	**K**	0.1231	5.6832	1	4
**G**	**L**	0.0986	5.6146	1	4
**G**	**N**	0.2578	5.3092	1	3
**G**	**P**	0.1575	5.2506	1	3
**G**	**Q**	0.2635	5.4865	1	3
**G**	**R**	0.3353	5.7671	1	3
**G**	**S**	0.1426	5.0539	1	3
**G**	**T**	0.1158	5.2921	1	3
**G**	**V**	0.0939	5.4806	1	4
**G**	**W**	0.5393	5.8808	1	3
**G**	**Y**	0.4195	5.7144	1	3
**H**	**H**	0.0524	6.3378	1	4
**H**	**N**	0.4937	6.1306	1	3
**H**	**P**	0.3934	6.0720	1	3
**H**	**C**	0.3852	6.0311	1	3
**H**	**I**	0.3252	6.6297	1	3
**I**	**I**	0.0057	6.9217	1	12
**K**	**K**	0.0455	6.6713	1	5
**K**	**T**	0.0674	6.2802	1	4
**K**	**R**	0.2167	6.7552	1	3
**K**	**A**	0.0847	5.9707	1	4
**K**	**D**	0.0009	6.2474	1	9
**K**	**E**	0.0009	6.4245	1	9
**K**	**Y**	0.1842	6.7025	1	3
**K**	**V**	0.0442	6.4687	1	5
**K**	**L**	0.0492	6.6027	1	5
**K**	**Q**	0.2241	6.4746	1	3
**K**	**W**	0.1984	6.8690	1	3
**K**	**F**	0.2061	6.6504	1	3
**K**	**S**	0.0959	6.0420	1	4
**K**	**H**	0.1663	6.5046	1	3
**K**	**N**	0.2180	6.2973	1	3
**K**	**P**	0.1117	6.2388	1	4
**K**	**C**	0.1030	6.1979	1	4
**K**	**I**	0.0394	6.7965	1	5
**L**	**L**	0.0242	6.5341	1	6
**L**	**Q**	0.1891	6.4060	1	3
**L**	**W**	0.4687	6.8003	1	3
**L**	**F**	0.3265	6.5818	1	3
**L**	**S**	0.0682	5.9734	1	4
**L**	**H**	0.3345	6.4359	1	3
**L**	**N**	0.1833	6.2287	1	3
**L**	**P**	0.0831	6.1701	1	4
**L**	**C**	0.0749	6.1292	1	4
**L**	**I**	0.0149	6.7279	1	6
**M**	**A**	0.0825	5.8690	1	4
**M**	**C**	0.0998	6.0962	1	4
**M**	**D**	0.1280	6.1457	1	3
**M**	**E**	0.1354	6.3228	1	3
**M**	**F**	0.3507	6.5488	1	3
**M**	**G**	0.1234	5.5815	1	3
**M**	**H**	0.3593	6.4029	1	3
**M**	**I**	0.0398	6.6948	1	5
**M**	**K**	0.0706	6.5696	1	4
**M**	**L**	0.0490	6.5010	1	4
**M**	**M**	0.0739	6.4680	1	4
**M**	**N**	0.2082	6.1957	1	3
**M**	**P**	0.1079	6.1371	1	4

**M**	**Q**	0.2140	6.3729	1	3
**M**	**R**	0.2876	6.6535	1	3
**M**	**S**	0.0931	5.9403	1	4
**M**	**T**	0.0662	6.1785	1	4
**M**	**V**	0.0443	6.3670	1	5
**M**	**W**	0.4923	6.7673	1	3
**M**	**Y**	0.3727	6.6008	1	3
**N**	**N**	0.3425	5.9234	1	3
**N**	**P**	0.2423	5.8648	1	3
**N**	**C**	0.2341	5.8239	1	3
**N**	**I**	0.1741	6.4225	1	3
**P**	**P**	0.1420	5.8062	1	3
**P**	**C**	0.1338	5.7653	1	3
**P**	**I**	0.0738	6.3639	1	4
**Q**	**Q**	0.3540	6.2779	1	3
**Q**	**W**	0.6252	6.6722	1	2
**Q**	**F**	0.4824	6.4537	1	3
**Q**	**S**	0.2332	5.8453	1	3
**Q**	**H**	0.4994	6.3078	1	3
**Q**	**N**	0.3483	6.1006	1	3
**Q**	**P**	0.2480	6.0420	1	3
**Q**	**C**	0.2399	6.0011	1	3
**Q**	**I**	0.1799	6.5998	1	3
**R**	**R**	0.1507	6.8391	1	3
**R**	**A**	0.2959	6.0546	1	3
**R**	**D**	0.0067	6.3313	1	7
**R**	**E**	0.0069	6.5084	1	7
**R**	**Y**	0.8042	6.7863	1	2
**R**	**V**	0.2591	6.5525	1	3
**R**	**L**	0.2636	6.6866	1	3
**R**	**Q**	0.4225	6.5585	1	3
**R**	**W**	0.9341	6.9528	1	2
**R**	**F**	0.7170	6.7343	1	2
**R**	**S**	0.3061	6.1259	1	3
**R**	**H**	0.2083	6.5884	1	3
**R**	**N**	0.4170	6.3812	1	3
**R**	**P**	0.3204	6.3226	1	3
**R**	**C**	0.3125	6.2817	1	3
**R**	**I**	0.2547	6.8804	1	3
**S**	**S**	0.1123	5.4127	1	4
**S**	**H**	0.3785	5.8752	1	3
**S**	**N**	0.2274	5.6680	1	3
**S**	**P**	0.1271	5.6094	1	3
**S**	**C**	0.1190	5.5685	1	3
**S**	**I**	0.0590	6.1672	1	4
**T**	**T**	0.0586	5.8891	1	4
**T**	**R**	0.2802	6.3641	1	3
**T**	**A**	0.0749	5.5796	1	4
**T**	**D**	0.1206	5.8563	1	3
**T**	**E**	0.1280	6.0334	1	3
**T**	**Y**	0.3654	6.3113	1	3
**T**	**V**	0.0366	6.0775	1	5
**T**	**L**	0.0414	6.2116	1	5
**T**	**Q**	0.2063	6.0835	1	3
**T**	**W**	0.4850	6.4778	1	3
**T**	**F**	0.3428	6.2593	1	3
**T**	**S**	0.0854	5.6509	1	4
**T**	**H**	0.3517	6.1134	1	3
**T**	**N**	0.2006	5.9062	1	3
**T**	**P**	0.1003	5.8476	1	4

**T**	**C**	0.0921	5.8067	1	4
**T**	**I**	0.0321	6.4054	1	5
**V**	**V**	0.0147	6.2660	1	6
**V**	**L**	0.0194	6.4000	1	6
**V**	**Q**	0.1844	6.2719	1	3
**V**	**W**	0.4642	6.6663	1	3
**V**	**F**	0.3220	6.4478	1	3
**V**	**S**	0.0635	5.8393	1	4
**V**	**H**	0.3297	6.3019	1	3
**V**	**N**	0.1786	6.0947	1	3
**V**	**P**	0.0784	6.0361	1	4
**V**	**C**	0.0702	5.9952	1	4
**V**	**I**	0.0102	6.5938	1	7
**W**	**W**	0.8031	7.0666	1	2
**W**	**F**	0.7703	6.8481	1	2
**W**	**S**	0.5105	6.2396	1	3
**W**	**H**	0.7632	6.7022	1	2
**W**	**N**	0.6198	6.4950	1	2
**W**	**P**	0.5246	6.4364	1	3
**W**	**C**	0.5169	6.3955	1	3
**W**	**I**	0.4599	6.9941	1	3
**Y**	**Y**	0.6458	6.7336	1	2
**Y**	**V**	0.3447	6.4998	1	3
**Y**	**L**	0.3492	6.6339	1	3
**Y**	**Q**	0.5050	6.5057	1	3
**Y**	**W**	0.7929	6.9001	1	2
**Y**	**F**	0.6231	6.6816	1	2
**Y**	**S**	0.3908	6.0732	1	3
**Y**	**H**	0.6424	6.5357	1	2
**Y**	**N**	0.4996	6.3285	1	3
**Y**	**P**	0.4048	6.2699	1	3
**Y**	**C**	0.3971	6.2290	1	3
**Y**	**I**	0.3404	6.8277	1	3
**M**	**U**	0.1723	7.3190	1	3
**G**	**U**	0.2007	6.4326	1	3
**K**	**U**	0.0972	7.4207	1	3
**T**	**U**	0.1679	7.0295	1	3
**R**	**U**	0.3949	7.5045	1	3
**A**	**U**	0.1772	6.7200	1	3
**D**	**U**	0.1920	6.9968	1	3
**E**	**U**	0.1953	7.1738	1	3
**Y**	**U**	0.6621	7.4518	1	3
**V**	**U**	0.1553	7.2180	1	3
**L**	**U**	0.1580	7.3520	1	3
**Q**	**U**	0.2527	7.2239	1	3
**W**	**U**	0.4276	7.6183	1	3
**F**	**U**	0.3483	7.3998	1	3
**S**	**U**	0.1833	6.7913	1	3
**H**	**U**	0.1661	7.2539	1	3
**N**	**U**	0.2494	7.0467	1	3
**P**	**U**	0.1918	6.9881	1	3
**C**	**U**	0.1872	6.9472	1	3
**I**	**U**	0.1527	7.5458	1	3
**U**	**U**	0.1100	8.1700	1	3

TABLE S5: Parameters of the Mpipi-Recharged

[1] H. Yamazaki, M. Takagi, H. Kosako, T. Hirano, and S. H. Yoshimura, "Cell cycle-specific phase separation regulated by protein charge blockiness," *Nature Cell Biology*, vol. 24, no. 5, pp. 625–632, 2022.

[2] K. Araki, N. Yagi, R. Nakatani, H. Sekiguchi, M. So, H. Yagi, N. Ohta, Y. Nagai, Y. Goto, and H. Mochizuki, "A small-angle x-ray scattering study of alpha-synuclein from human red blood cells," *Scientific reports*, vol. 6, no. 1, p. 30473, 2016.

[3] M. Kjaergaard, A.-B. Nørholm, R. Hendus-Altenburger, S. F. Pedersen, F. M. Poulsen, and B. B. Kragelund, "Temperature-dependent structural changes in intrinsically disordered proteins: Formation of α–helices or loss of polyproline ii?," *Protein Science*, vol. 19, no. 8, pp. 1555–1564, 2010.

[4] E. W. Martin, A. S. Holehouse, C. R. Grace, A. Hughes, R. V. Pappu, and T. Mittag, "Sequence determinants of the conformational properties of an intrinsically disordered protein prior to and upon multisite phosphorylation," *Journal of the American Chemical Society*, vol. 138, no. 47, pp. 15323–15335, 2016.

[5] J. A. Riback, M. A. Bowman, A. M. Zmyslowski, C. R. Knoverek, J. M. Jumper, J. R. Hinshaw, E. B. Kaye, K. F. Freed, P. L. Clark, and T. R. Sosnick, "Innovative scattering analysis shows that hydrophobic disordered proteins are expanded in water," *Science*, vol. 358, no. 6360, pp. 238–241, 2017.

[6] G. Fuertes, N. Banterle, K. M. Ruff, A. Chowdhury, D. Mercadante, C. Koehler, M. Kachala, G. Estrada Girona, S. Milles, A. Mishra, *et al.*, "Decoupling of size and shape fluctuations in heteropolymeric sequences reconciles discrepancies in SAXS vs. FRET measurements," *Proceedings of the National Academy of Sciences*, vol. 114, no. 31, pp. E6342–E6351, 2017.

[7] E. Mylonas, A. Hascher, P. Bernado, M. Blackledge, E. Mandelkow, and D. I. Svergun, "Domain conformation of tau protein studied by solution small-angle x-ray scattering," *Biochemistry*, vol. 47, no. 39, pp. 10345–10353, 2008.

[8] M. Wells, H. Tidow, T. J. Rutherford, P. Markwick, M. R. Jensen, E. Mylonas, D. I. Svergun, M. Blackledge, and A. R. Fersht, "Structure of tumor suppressor p53 and its intrinsically disordered N-terminal transactivation domain," *Proceedings of the National academy of Sciences*, vol. 105, no. 15, pp. 5762–5767, 2008.

[9] V. N. Uversky, J. R. Gillespie, and A. L. Fink, "Why are "natively unfolded" proteins unstructured under physiologic conditions?," *Proteins: structure, function, and bioinformatics*, vol. 41, no. 3, pp. 415–427, 2000.

[10] U. Baul, D. Chakraborty, M. L. Mugnai, J. E. Straub, and D. Thirumalai, "Sequence effects on size, shape, and structural heterogeneity in intrinsically disordered proteins," *The Journal of Physical Chemistry B*, vol. 123, no. 16, pp. 3462–3474, 2019.

[11] M. Arbesú, M. Maffei, T. N. Cordeiro, J. M. Teixeira, Y. Pérez, P. Bernadó, S. Roche, and M. Pons, "The unique domain forms a fuzzy intramolecular complex in src family kinases," *Structure*, vol. 25, no. 4, pp. 630–640, 2017.

[12] G.-N. W. Gomes, M. Krzeminski, A. Namini, E. W. Martin, T. Mittag, T. Head-Gordon, J. D. Forman-Kay, and C. C. Gradinaru, "Conformational ensembles of an intrinsically disordered protein consistent with NMR, SAXS, and single-molecule FRET," *Journal of the American Chemical Society*, vol. 142, no. 37, pp. 15697–15710, 2020.

[13] A. Bremer, M. Farag, W. M. Borcherds, I. Peran, E. W. Martin, R. V. Pappu, and T. Mittag, "Deciphering how naturally occurring sequence features impact the phase behaviours of disordered prion-like domains," *Nature Chemistry*, vol. 14, no. 2, pp. 196–207, 2022.

[14] J. A. Joseph, A. Reinhardt, A. Aguirre, P. Y. Chew, K. O. Russell, J. R. Espinosa, A. Garaizar, and R. Collepardo-Guevara, "Physics-driven coarse-grained model for biomolecular phase separation with near-quantitative accuracy," *Nature Computational Science*, vol. 1, no. 11, pp. 732–743, 2021.

[15] J. P. Brady, P. J. Farber, A. Sekhar, Y.-H. Lin, R. Huang, A. Bah, T. J. Nott, H. S. Chan, A. J. Baldwin, J. D. Forman-Kay, *et al.*, "Structural and hydrodynamic properties of an intrinsically disordered region of a germ cell-specific protein on phase separation," *Proceedings of the National Academy of Sciences*, vol. 114, no. 39, pp. E8194–E8203, 2017.

[16] S. Maharana, J. Wang, D. K. Papadopoulos, D. Richter, A. Pozniakovsky, I. Poser, M. Bickle, S. Rizk, J. Guillen-Boixet, T. M. Franzmann, M. Jahnel, L. Marrone, Y.-T. Chang, J. Sterneckert, P. Tomancak, A. A. Hyman, and S. Alberti, "RNA buffers the phase separation behavior of prion-like RNA binding proteins," *Science*, vol. 360, no. 6391, pp. 918–921, 2018.

[17] J. Wang, J.-M. Choi, A. S. Holehouse, H. O. Lee, X. Zhang, M. Jahnel, S. Maharana,

R. Lemaitre, A. Pozniakovsky, D. Drechsel, *et al.*, "A molecular grammar governing the driving forces for phase separation of prion-like RNA binding proteins," *Cell*, vol. 174, no. 3, pp. 688–699, 2018.

[18] K. A. Burke, A. M. Janke, C. L. Rhine, and N. L. Fawzi, "Residue-by-residue view of in vitro FUS granules that bind the c-terminal domain of RNA polymerase ii," *Molecular cell*, vol. 60, no. 2, pp. 231–241, 2015.

[19] A. Molliex, J. Temirov, J. Lee, M. Coughlin, A. P. Kanagaraj, H. J. Kim, T. Mittag, and J. P. Taylor, "Phase separation by low complexity domains promotes stress granule assembly and drives pathological fibrillization," *Cell*, vol. 163, no. 1, pp. 123–133, 2015.

[20] Q. Li, X. Peng, Y. Li, W. Tang, J. Zhu, J. Huang, Y. Qi, and Z. Zhang, "Llpsdb: a database of proteins undergoing liquid–liquid phase separation in vitro," *Nucleic acids research*, vol. 48, no. D1, pp. D320–D327, 2020.

[21] A. G. Larson, D. Elnatan, M. M. Keenen, M. J. Trnka, J. B. Johnston, A. L. Burlingame, D. A. Agard, S. Redding, and G. J. Narlikar, "Liquid droplet formation by HP1α suggests a role for phase separation in heterochromatin," *Nature*, vol. 547, no. 7662, pp. 236–240, 2017.

[22] L. McGurk, E. Gomes, L. Guo, J. Mojsilovic-Petrovic, V. Tran, R. G. Kalb, J. Shorter, and N. M. Bonini, "Poly (adp-ribose) prevents pathological phase separation of TDP-43 by promoting liquid demixing and stress granule localization," *Molecular cell*, vol. 71, no. 5, pp. 703–717, 2018.

[23] A. E. Conicella, G. L. Dignon, G. H. Zerze, H. B. Schmidt, M. Alexandra, Y. C. Kim, R. Rohatgi, Y. M. Ayala, J. Mittal, and N. L. Fawzi, "TDP-43 α-helical structure tunes liquid–liquid phase separation and function," *Proceedings of the National Academy of Sciences*, vol. 117, no. 11, pp. 5883–5894, 2020.

[24] P. R. Banerjee, A. N. Milin, M. M. Moosa, P. L. Onuchic, and A. A. Deniz, "Reentrant phase transition drives dynamic substructure formation in ribonucleoprotein droplets," *Angewandte Chemie*, vol. 129, no. 38, pp. 11512–11517, 2017.
