# Supplementary material for: Chemically Informed Coarse-Graining of Electrostatic Forces in Charge-Rich Biomolecular Condensates
Source: ACS Cent Sci. 2025 Feb 11;11(2):302–21. doi: 10.1021/acscentsci.4c01617 (PMC11869137; doi:10.1021/acscentsci.4c01617)
Supplement: Supplementary file 2 — oc4c01617_si_002.pdf [file oc4c01617_si_002.pdf]

oc-2024-016178.R1

Name: Peer Review Information for "Chemically-informed coarse-graining of electrostatic forces in charge-rich biomolecular condensates"

First Round of Reviewer Comments

Reviewer: 1

Comments to the Author

The ability to simulate the behaviour of biomolecular condensates is important, and the Mpipi force field has already proven to be a valuable model in this field. Here, the authors describe a new version of the model, Mpipi-Recharged, which introduces more accurate potentials for charged amino acids. Following the original parametrization strategy underlying Mpipi, the new potentials are based on PMF calculations of amino acid pairs performed at the all-atom level.

Although I appreciate the importance of improving force fields in general, the current work has three major shortcomings in my view.

1) It remains largely unclear how the new potentials were obtained and why certain choices were made:

With respect to the atomistic PMFs, it remains unclear whether or not only the side chains were considered, or entire amino acids. Furthermore, it is mentioned that the amino acids were restrained, but it is unclear why and how this would correspond to the way these amino acids interact with each other inside an actual condensate.

Worryingly, the actual potentials that are parametrized are not compared to the target PMFs, so we cannot judge how closely Mpipi-Recharged manages to reproduce the atomistic PMFs. And why not use the PMFs directly to model the interaction between charged residues (either using tabulated potentials or by polynomial fitting) ?

The screening of interactions pertaining folded domains is also done rather ad-hoc, using a single property (critical solution temperature) to inform the model. No validation on interactions between

folded proteins is presented - how well does Mpipi-Recharged model dimerization or higher-order oligomerization of soluble, folded, proteins ?

2) The apparent improved behaviour of Mpipi-Recharged versus Mpipi, as claimed by the authors, is largely exaggerated, overselling the results:

For instance, the Rg of the old Mpipi model in fact shows better agreement with experiment than the improved version (which clearly overshoots the Rg for longer IDPs). The correlation between predicted critical temperature and experimental saturation concentrations (Fig 2d) shows only marginal improvement for the new model, and the same can be said for the results shown in Fig 3.

Finally the ability to reproduce phase behaviour of condensates with polyU is not a stringent test, as this is largely governed by charge neutralization, which should be captured with even the simplest one-bead-per-residue models with explicit charges.

3) No discussion whatsoever is presented on the possible shortcomings of the new model, the underlying assumptions, and limitations:

How would the choice of all-atom force field influence the results? How can the model be applied to state points (ionic strength, temperature) other than those used for the reference PMFs ?

The authors provide some rationale for the use of Mpipi over all-atom models, but how about explicit solvent CG approaches such as Martini and SPICA? And how does Mpipi-Recharged compares to other one-bead-per-residue models such as Calvados and HPS ?

Finally, the claim that asymmetric potentials are required for an accurate description of charged IDPs seems plausible, but actually no proof is given other than that Mpipi-Recharged (marginally) improves the behavior of Mpipi. But is this a direct consequence of the asymmetric potentials? This is not clear.

Reviewer: 2

Comments to the Author

In this manuscript, the authors present the Mpipi-Recharged model, an implicit-solvent, residue-resolution coarse-grained (CG) model aiming to resolve challenges in describing charge e=ects in biomolecular phase behavior present in predecessor models (such as Mpipi). Instead of using a symmetric description of charge–charge interactions, the model adopts an asymmetric, pair-specific Yukawa potential to recapitulate the PMF of atomistic simulations of explicit solvent and ions they've sampled, enabling the accurate capture of asymmetry and phase behavior of highly charged proteins and complex coacervates. The authors validate the model against the same experimental results used to validate the Mpipi model, such as single-molecule radii of gyration of many IDPs, critical solution temperatures, and saturation concentrations. The model also finds agreement with experimental results for descriptions of charge-blockiness, a challenge to many residueresolution CG models.

Overall, the manuscript demonstrates an improvement from a class of implicit-solvent CG models aimed to investigate phase behaviors in solvated biomolecular systems and are in general congruence with experimental results. However, I provide here several major and minor issues the authors should address:

Major comments:

- The authors should report standard errors on all plots of PMFs, such as Figure 1.
- The authors should discuss the performance of this model with available, machinelearned, implicit-solvent models for similar biomolecular systems of interest, such as the model presented by Airas J, Ding X, and Zhang B, *ACS Cent Sci*, 2023.

Minor comments:

- When abbreviating terms, the authors should introduce the full term before using its abbreviations, such as "IDRs" ("intrinsically disordered regions") and "IDPs" ("intrinsically disordered proteins").
- The authors should abbreviate terms that are repeated many times, such as "molecular dynamics" (MD), "coarse-grained" (CG).
- The authors should not abbreviate terms that are only used one time, such as "Weighted Histogram Analysis Method."
- On page 6, the authors misspelled "asymmetric."
- The authors did not provide section numbers after many instances of phrase "see section," such as on pages 8, 12, 18, and 21.

Reviewer: 3

Comments to the Author

The authors present one step further in their efforts to characterize condensates, especially those leading to liquid phases related to IDPs and structure-less nucleic acids. The work is focused on electrostatic model. The authors followed ideas of already existing models (including their Mpipi one) which use a high level of coarse graining, where details of the side chains are ignored. The idea here is to use a slightly different electrostatic description that is parametrized using atomistic simulations as reference and is validate with a large range of macroscopic descriptors of disordered proteins and some phase diagrams. The paper is very interesting, and there is a huge amount of work presented here. I believe that it merits publication, but I believe the authors should take into consideration comments below:

There is a huge amount of papers on the biases of atomistic simulations of charge-charge interactions. Some authors use specific tuning of van der Waals terms to correct over-stabilization of complexes, others played with more polar solvent models, even an increasing number of authors scale down directly the charges (following a complex reasoning on effective dielectric constants). All this tuning-activity activity reflects the unpleasant fact that atomistic representations of ion pairs are not necessarily correct, and the authors should provide evidence that they are representing well the experimental reality. For example, lysine acetate or arginine acetate are (I think) soluble in water. Can author's simulations represent this? On the same lines, the minimum of a PMF should not be interpreted as a binding free energy, the PMF needs to be integrated, and this is especially important for multiple minima PMFs. Caution is needed as this seems to be a bottom-up coarse grained model. I would recommend some checking of the reference calculations.

Eq. 2 is extended to all bonds, understanding sequential contacts only, right? no need to add additional terms? Do they reproduce the sampled "bonded" geometries of IDPs in atomistic simulations (using a IDP-friendly FF)? Can the authors comment on this?

I am curious on the need to use WF potential for vW interactions. It is very complex, and probably not the most efficient one from a computational point of view. Many fitted parameters improve flexibility, but also the risk of overtraining. Can the authors comment on this?

I must recognize that I did not know Yukawa potential, but looking at eq. 6, it looks like a scaled Debye Huckel, where the term QQ/epsilon is a fitted variable. Could they discuss about what is the effective charge that are implicit to the fitted Aij terms depending on the pairs? This will help to convince on the lack of biases in training.

The authors should discuss how much of the fitted parameters are dependent on the vW terms they are using. Playing simultaneously with effective size and the charge improve fitting capabilities, but also overtraining risks. Some calculations on the final model using small systems would help to convince the reader

Format of some references are wrong.

In summary, despite my comments above, I am very positive on this paper.

Author's Response to Peer Review Comments:

Please find attached the response to the comments raised by the reviewers.

Reviewer 1

The ability to simulate the behaviour of biomolecular condensates is important, and the Mpipi force field has already proven to be a valuable model in this field. Here, the authors describe a new version of the model, Mpipi-Recharged, which introduces more accurate potentials for charged amino acids. Following the original parametrization strategy underlying Mpipi, the new potentials are **based on PMF calculations** of amino acid pairs performed at the all-atom level.

Although I appreciate the importance of improving force fields in general, the current work has three major shortcomings in my view.

We kindly thank the reviewer for recognizing the impact of the Mpipi model in the field, and for providing a set of comments that have helped us to improve the outcome and impact of our present work. Please find below a detailed response on the different comments, questions, and concerns pointed out by the reviewer.

1) It remains largely unclear **how the new potentials were obtained** and why certain choices were made:

With respect to the atomistic PMFs, it remains unclear whether or not only the side chains were considered, or **entire amino acids**.

We thank the reviewer for highlighting the need for additional clarity in the description of our atomistic PMF calculations. Our all-atom simulations were conducted using the entire residue, including the amine group, carboxyl group, and side chain. The N- and C-terminal ends were capped with acetyl and N-methyl groups, respectively.

Furthermore, it is mentioned that the amino acids were restrained, but it is unclear why and how this would correspond to the way these **amino acids interact with each other inside an actual condensate**

To the best of our knowledge, the precise orientations among amino acid pairs inside a biomolecular condensate have not yet been characterized experimentally for any system and under any experimental condition, and neither are the configurations of individual biomolecules inside condensates at high resolution. Thus, the way amino acids interact with each other inside an actual condensate is still unknown. There are extensive research efforts in this direction, and we are looking forward to notable advances in the coming years.

Given the lack of unequivocal information on the interaction modes of amino acids inside actual condensates, we focus on quantifying the strongest binding modes among each amino acid pair. Quantifying this is particularly insightful because it reveals an upper

bound for the interaction potential of each amino acid pair, providing critical insights into which pairs are more likely to contribute to the formation of the liquid network within condensates. It is well known that the strongest interacting residues form the anchor points of the network of molecular interactions in the condensates (e.g. Bremer et al Nat Chem 2022, Martin et al Science 2020).

To characterise this interaction, we follow a methodology that has been previously validated against experiments of biomolecular condensates in multiple publications of our group, such as Krainer *et al., Nat. Communs*. 2021; Joseph *et al., Nat. Comput. Sci*. 2021, or Torrino *et al., Cell*, 2024. Specifically, in our umbrella sampling simulations the amino acids in each pair are oriented and restrained so that their side chains face each other. Their arrangements are based on the most common configurations observed in protein structures, because these are expected to correspond to their strongest binding modes. In cases where the preferred interaction was ambiguous, multiple orientations have been tested to identify those yielding the strongest interaction (Joseph et al. Nat Comp Scie 2021 and Krainer et al Nat Communs 2021).

At the PMF minima for each amino acid pair configuration, the side chains exhibit the smallest molecular separation within the residue, resulting in side chain–side chain interactions dominating the behaviour. However, the PMF also incorporates inter-residue contributions from the other groups in the amino acids (e.g. backbone to side chain), given the size of the residue relative to the interaction potential cut-off of 1.4 nm.

We have now clarified this relevant point in the Methods Section (pages 32 and 33) of our manuscript.

Worryingly, the actual potentials that **are parametrized are not compared to the target PMFs**, so we cannot judge how closely Mpipi-Recharged manages to reproduce the atomistic PMFs. And why **not use the PMFs directly** to model the interaction between charged residues (either using tabulated potentials or by polynomial fitting)?

Thank you for highlighting this point, which we fully agree requires clarification. In response, we have added a direct comparison between the parametrised potentials in Mpipi-Recharged and the target PMFs in the new Figures S15 and S16, similar to the comparison shown for Mpipi in our previous work (Joseph et al., Nat. Comput. Sci. 2021; Figure S3).

A critical feature of our model, as well as other residue-resolution coarse-grained models such as the HPS and CALVADOS families, is its ability to decouple the contributions of ionic and non-ionic forces to phase separation and to approximately probe electrostatic screening effects. This capability relies on the combination of a Lennard-Jones-like potential (the Wang-Frenkel potential in our case) to capture non-ionic interactions and a Yukawa-like potential to represent ionic interactions. However, this combination of

potentials complicates the direct fitting of the Yukawa potential parameters to the PMF curves of charged pairs. The PMF curves represent the overall interaction free energy for specific configurations, reflecting not just electrostatic contributions but also other forces, such as van der Waals and hydrophobic interactions. Since the relative contributions of these forces are not well-defined, this introduces an additional free parameter into the model. To address this, we use the PMF results as a reference to balance the relative contributions of electrostatic attraction and repulsion. Specifically, as we did for parameters of the Wang-Frenkel potential in the original Mpipi model before (Joseph et al Nat Comp Scie 2021), we compute the integrals of the well depths in our atomistic PMFs and normalize them by the value of the strongest interacting pair (in this case R-D). Then we adjust the values of the prefactor A_{ij} by a suitable multiplicative factor so that the normalized PMF integrals are reasonably approximated by the normalized integrals of the Mpipi-Recharged model (considering both the Yukawa and the Wang-Frenkel contribution). This comparison is shown in Fig. S16. In Table S4 we report the actual partial charges that each pair of residues would effectively establish at coarse-grained level if a standard Columbic/Debye Huckel potential would have been used instead of the Yukawa. Importantly, atomistic simulations such as PMF calculations are often used as we have done, i.e. a framework to guide the parametrization of coarse-grained models but not directly translated into parameters of the coarse-grained model (see 10.1073/pnas.2119800119, 10.1038/s41467-022-32874-0, 10.1016/j.cell.2024.10.048, and 10.1038/s41467-021-21181-9). We have added a discussion on this important point clarifying how the PMF calculations are used for the modelling parameterization strategy in pages 11, 31-33.

Finally, we want to emphasise that all coarse-grained models are inherently approximate and involve design decisions, such as the choice of potentials, resolution, bead characteristics, and interaction types. These choices must align with the specific questions being addressed. As highlighted by Choi et al. (PLoS Comput. Biol. 15, e1007028, 2019), there is no universally superior approach to coarse-grained model design or parameterisation—each has its strengths and limitations. While using tabulated potentials or polynomial fitting, as suggested by the reviewer, is an alternative design choice, it would compromise the ability of our model to decouple ionic and non-ionic forces and to probe electrostatic screening effects. This would diminish the model’s capacity to our applications of interest, such as investigating salt-dependent complex coacervation and RNA-driven reentrant phase transitions, and would not inherently improve the model's accuracy or predictive power.

The success of a coarse-grained model ultimately lies in its ability to reproduce experimentally observed behaviours while providing physicochemical and molecular insights that are inaccessible through experiments alone. Our model achieves this

balance, offering both simplicity and interpretability without sacrificing predictive accuracy.

The screening of interactions pertaining **folded domains is also done rather ad-hoc**, using a single property (critical solution temperature) to inform the model. No validation on interactions between folded proteins is presented - **how well does Mpipi-Recharged model dimerization or higher-order oligomerization of soluble, folded, proteins**?

The approach adopted in Mpipi-Recharged (i.e., interaction rescaling) has been widely used in coarse-grained models to account for buried residues within globular domains, employing various rescaling factors (as detailed in Refs. 10.1371/journal.pcbi.1005941, 10.1016/j.bpj.2021.11.003, and 10.1038/s43588-021-00155-3). However, in these prior studies, the effect of the applied rescaling factor on the experimental trends of phase separation was not rigorously validated. In Figs. 4c and S7 of our work, we address this gap by testing different rescaling factors for globular–globular and globular–disordered interactions and comparing the critical temperatures predicted by our model with experimental in vitro saturation concentrations. We have recently demonstrated a strong correlation between the critical solution temperature and saturation concentration, providing a foundation for this comparison (see Feito et al., PLOS Comput. Biol., 2024 or https://www.biorxiv.org/content/10.1101/2024.08.28.610132v2).

Our results show that using a rescaling factor of 0.7 successfully predicts the relative thermodynamic stability of a test set of proteins, suggesting that this empirical approach is robust within the scope of our model. Furthermore, motivated by the reviewer's comment, we also tested an alternative strategy recently proposed for the CALVADOS model (Ref. 10.1002/pro.5172), which replaces rescaling with a bead repositioning scheme. Specifically, in this approach, beads within globular domains are recentered from the Cα position to the residue centre of mass (COM). While this method performs excellently for the CALVADOS model, we find it produces significantly poorer results for Mpipi-Recharged compared to the 0.7 rescaling factor. We hypothesise that this discrepancy arises from the different treatments of charge–charge interactions between Mpipi-Recharged and CALVADOS. Based on these findings, we are confident that our approach, while empirical, is a reasonable and effective method for predicting the phase-separation propensities of multi-domain proteins.

Additionally, we note that there is a scarcity of quantitative experimental data on the thermodynamic stability of biomolecular condensates under equivalent solution conditions. This limitation constrains the ability to validate such models rigorously. Many other models, including CALVADOS, have instead validated against single-molecule properties, such as the radius of gyration. However, as we have shown recently (https://doi.org/10.1080/00268976.2024.2425757), accurately capturing single-molecule properties does not necessarily correlate with the ability to predict the

thermodynamic stability or material properties of condensates. This highlights the importance of directly testing phase separation trends against experimental observations, as we have done in this work.

In light of the reviewer's critique, we have updated the manuscript on pages 18 and 19 to elaborate on this point and discuss the new results presented in Fig. S8.

2) The apparent improved behaviour of Mpipi-Recharged versus Mpipi, as claimed by the authors, is largely exaggerated, overselling the results:

For instance, the **Rg of the old Mpipi model in fact shows better agreement with experiment than the improved version** (which clearly overshoots the Rg for longer IDPs). The correlation between predicted critical temperature and experimental saturation concentrations (Fig 2d) shows only **marginal improvement for the new model**, and the same can be said for the results shown in Fig 3.

We thank the reviewer for pointing out another issue that needed clarity. Regarding this point, below we discuss the **objective results** we use to back up our claim that the Mpipi-Recharged model represents a significant improvement over our original Mpipi model. The original Mpipi marginally outperforms Mpipi-Recharged in predicting single-protein radii of gyration for IDPs. However, Mpipi-Recharged objectively outperforms Mpipi when describing the phase behaviour of highly charged phase-separating systems. Our interest in chromatin and other highly charged systems is the original reason that pushed us to improve the Mpipi model, which already performs excellently for non-charged systems.

1. When comparing the critical solution temperatures of the charged variants of A1-LCD predicted by the models versus the values extracted from experimental coexistence densities, **the correlation coefficient for the Mpipi-Recharged model predictions is 50% higher than that of the Mpipi model** (r=0.44 for Mpipi-Recharged vs r=0.29 for Mpipi, see Figs. 2d and Fig. S4). Moreover, the Mpipi-Recharged model demonstrates greater sensitivity to charged residue mutations, as evidenced by the predicted critical solution temperatures of the charge mutants (Fig. 2d) spanning nearly twice the temperature range compared to those predicted by the Mpipi model (Fig. S4). In this regard, for additional variants of A1-LCD, we have demonstrated the higher accuracy of Mpipi-Recharged over the original Mpipi in predicting the phase diagram and condensate material properties (see Feito et al., PLOS Comput. Biol., 2024). This point is discussed in detail on page 13.

2. The phase diagrams of DDX4 mutants predicted by the Mpipi-Recharged model align more closely with experimental observations compared to those predicted by the original Mpipi model. In vitro experiments show a critical temperature

difference of 35 K between the WT-DDX4 and CS-DDX4 variants. Mpipi-Recharged predicts this difference as 40 K, whereas Mpipi predicts only 10 K. Similarly, the saturation concentration predicted by Mpipi-Recharged at 100 mM NaCl for these two variants is closer to experimental values. Additionally, the experimental coexistence densities are better captured by Mpipi-Recharged, as shown in Figs. 3a and S5. At both 100mM and 300 mM NaCl, Mpipi-Recharged accurately predict the correct phase behaviour, even as the density differences narrow as salt increases (Fig. 3b). These differences are discussed in detail on page 14 of the manuscript.

3. Other examples where Mpipi-Recharge outperforms Mpipi are the RNA-driven reentrant phase behaviour and the phase diagrams of H1+ProTα. We discuss these two cases in more detail below and in our answer to point 3.

4. Additionally, in our recent works: https://doi.org/10.1101/2024.08.28.610132 and https://doi.org/10.1080/00268976.2024.2425757 we test the performance of the Mpipi-Recharged model against Mpipi and four other models, demonstrating that it outperforms the others when it comes to predicting stability and material properties of condensates.

Finally, the ability to reproduce phase behaviour of condensates with polyU is not a stringent test, as this is **largely governed by charge neutralization**, which should be captured with even **the simplest one-bead-per-residue models with explicit charges**.

We also thank the reviewer for their constructive criticism regarding the RNA-driven reentrant phase behaviour. As we show below, while apparently trivial, recapitulating the experimental RNA-driven reentrant phase behaviour of protein solutions quantitatively is challenging for one-bead-per-residue models. As correctly commented by the reviewer, the RNA re-entrant behaviour is mainly electrostatically driven, and therefore, the balance between attraction and repulsion has big implications for whether the maximum stability of the condensate occurs at the electroneutral point or not, and also in modulating the coexistence densities.

Indeed, the challenges we have found in our previous work investigating a variety of reentrant phase transitions of biomolecular solutions with coarse-grained models that describe electrostatic attraction and repulsion symmetrically (Krainer et al Nat Communs 2021; Sanchez-Burgos et al PloS Comp Bio 2022; Brown et al Cell Reports 2023; Sanchez-Burgos et al Biophys J, 2023) have motivated our development of the Mpipi-Recharged model.

As we showed in Fig. 5, the Mpipi-Recharged model quantitatively captures the maximum concentration of RNA (C0) that can be added into the RP3 and the SR8 systems to induce

maximum condensate stability before the condensate destabilization begins. This concentration occurs slightly beyond the electroneutral point (negative total charge).

Mpipi-Recharged:

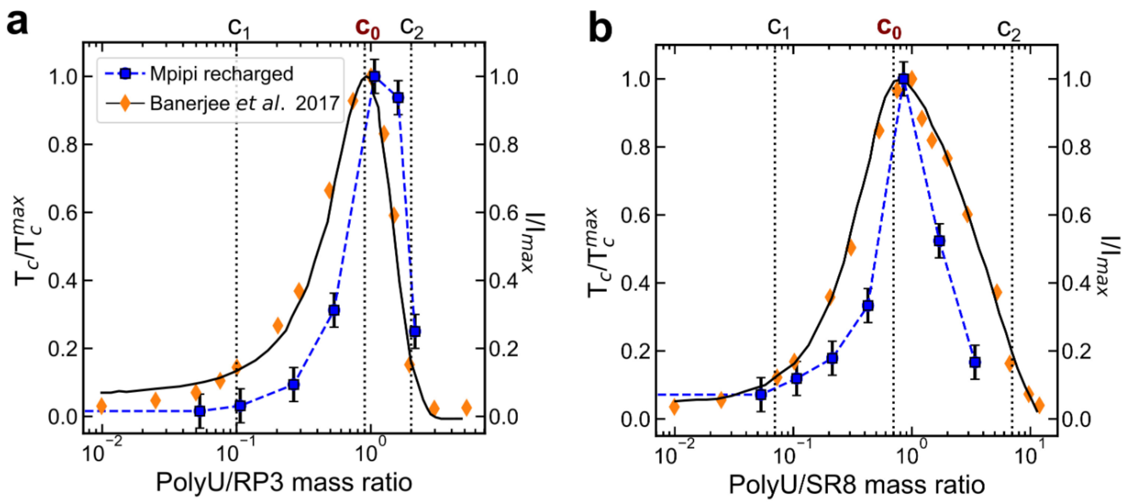

While the original Mpipi describes the direction of the change in condensate stability for RP3 and SR8 as RNA is added correctly, it overestimates the concentration C0 with respect to experiments. Furthermore, Mpipi overestimates the thermodynamic stability of the SR8 system at low RNA concentrations, likely due to the repulsion vs. attraction imbalance.

Mpipi: (Fig S11, already included in the original version of our manuscript)

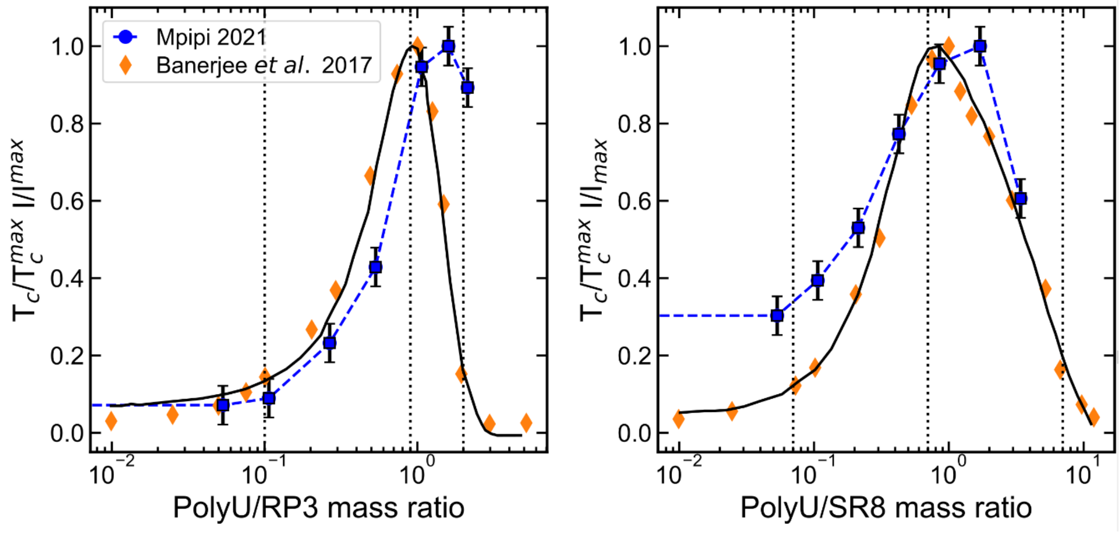

The HPS-cation-π model (new Fig. S12 shown below) is not able to capture the gradual increase (low RNA concentration) and gradual decrease (high RNA concentration) in condensate stability as the RNA concentration is monotonically increased. For both

systems systems, phase separation is only predicted in the close vicinity of the electroneutral point. A very narrow difference between C1 and C0 marks the change from no phase separation to maximum stability, and another narrow difference between C0 and C2 signals the change from maximum stability back to its dissolution. This behaviour contrasts starkly with the experimental results, where fluorescence intensities vary gradually across a much broader range of RNA concentrations. This discrepancy likely stems from an imbalance between electrostatic attraction among R-U and the repulsion among R-R and U-U. We have discussed the new figures in page 23.

HPS-cation-π:

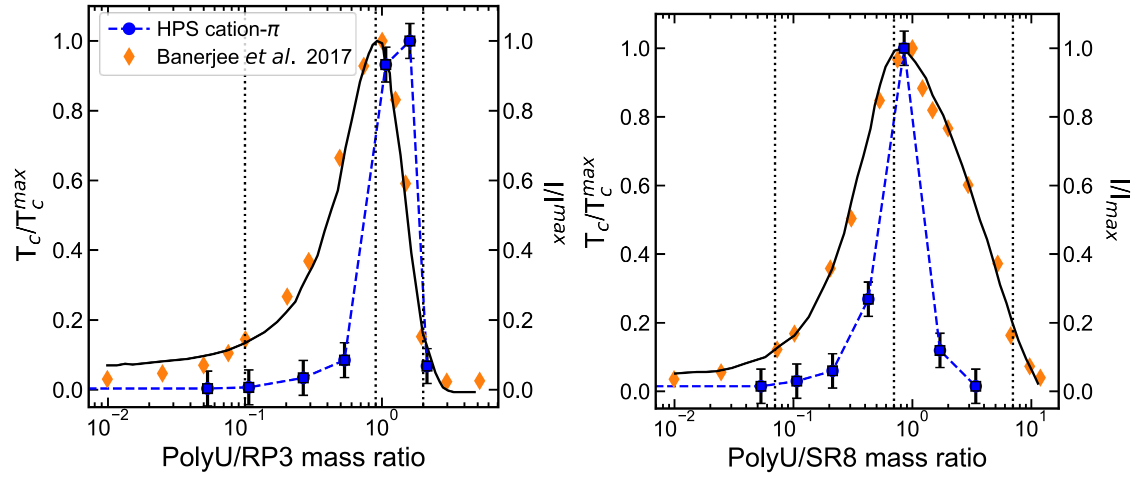

Hence, we conclude that even sophisticated residue-resolution models with point charges and symmetric screened potentials, such as the Mpipi or HPS-cation-π, cannot quantitatively reproduce such a simple behaviour because they fail to balance correctly attraction and repulsion.

3) No discussion whatsoever is presented on the possible shortcomings of the new model, the underlying assumptions, and limitations:

How would the **choice of all-atom force field influence the results**? How can the model be applied to state points (ionic strength, temperature) other than those used for the reference PMFs?

As commented in the previous question (point 1), our PMF calculations are not used to infer the exact energy associated to each interaction pair but rather to approximate the

relative strengths of interactions among the different atomistic charged pairs. Therefore, the choice of the atomistic force field is not expected to produce significant differences in our work. In previous work (Garaizar et al., PNAS 2022), we demonstrated that PMF estimates obtained using our approach were almost quantitatively identical to those derived using CHARMM36m (Ref. https://doi.org/10.1016/j.bpj.2016.11.971), confirming the robustness of the relative interaction strengths among amino acids across different force fields. Moreover, previous studies conducted by our group have already reported a similar asymmetric behaviour in the electrostatic repulsion versus attraction between charged amino acids (see Refs. 10.1016/j.bpj.2020.11.426 and 10.1016/j.cell.2024.10.048).

We have clarified this point throughout the manuscript and expanded the discussion on pages 11, 32, and 33 to provide further detail and context.

We are aware of the disadvantages and limitations intrinsic to the Mpipi-Recharged model. One of these limitations is studying effects in the phase behaviour that are markedly determined by the solvent or ions, such as water entropic effects, the effect of pH, salt gradients, high ionic concentration, presence of multivalent ions, or ion density fluctuations either in the diluted or in the condensed phase. We have included a new section ("Limitations of the Mpipi-Recharged") discussing these different points in detail in pages 25 and 26 of main text.

The authors provide some rationale for the use of Mpipi over all-atom models, but how about **explicit solvent CG approaches such as Martini and SPICA**?

The models mentioned by the reviewer, like all coarse-grained models, have their own strengths and limitations. Due to their approximate nature, they are best suited for the specific questions and systems for which they were designed. Both Martini and SPICA were developed and parameterised to accurately capture the behaviour of lipids and proteins and their interactions, particularly within membrane environments.

While **Martini** has recently been applied to biomolecular condensates made of peptides, its application in this area is still in development, and we look forward to further advancements. However, as expected from its original design and parameterisation—which was optimised to capture specific, long-lived protein–protein binding and oligomerisation interactions rather than dynamic biomolecular condensates—the results so far suggest limitations. Specifically, tests indicate that protein–protein interactions are overestimated, while protein–water interactions are underestimated. This imbalance leads to a severe overestimation of the condensed-phase coexistence density (see Refs. https://pubs.acs.org/doi/abs/10.1021/acs.jctc.2c01273 and https://pubs.acs.org/

doi/full/10.1021/acs.jctc.0c01064). To our knowledge, **SPICA** has not yet been tested on biomolecular condensates.

Furthermore, all models explicitly treating solvent and ions dramatically increase the computational cost. This makes it challenging to simulate condensates beyond those made of small peptides (more than ~20 residues per protein) using the direct coexistence simulation method because the larger the protein, the larger the size of the box, and the more water molecules needed to fill the elongated boxes (3-5 times the length of the condensed phase). As we move into complex coacervates or other multicomponent systems, a larger number of biomolecules is needed (and subsequently larger boxes and even more water) to reduce finite-size effects related to maintaining the system composition. Such limitations become especially problematic when attempting to measure condensate material properties, such as viscosity, which require simulations over longer timescales and larger system sizes; these are already at the limit of models like Mpipi-Recharged.

And how does Mpipi-Recharged compares to other one-bead-per-residue models such as **Calvados and HPS**?

Besides the comparisons with Mpipi and HPS-cation-π discussed above, we have recently benchmarked the new Mpipi-Recharged model against the five most widely used one-bead-per-residue models: HPS, HPS-Urry, HPS-cation-π, CALVADOS2 and Mpipi. These benchmarks can be found here:
doi: https://doi.org/10.1101/2024.08.28.610132 and https://doi.org/10.1080/00268976.2024.2425757. We have added references to these recent works in the main.

In these benchmarks, we observe that most models predict single-molecule radius of gyrations of IDPs in Good agreement with experimental values. Despite this, not all models predict satisfactorily the range of stability (critical solution temperature and saturation concentration) and material properties (viscosity) of their condensates, and the Mpipi-Recharged model outperforms all the models for the proteins tested.

Finally, the claim that asymmetric potentials are required for an accurate description of charged IDPs seems plausible, but actually **no proof is given other than that Mpipi-Recharged (marginally) improves the behavior of Mpipi**. But is this a **direct consequence of the asymmetric potentials**? This is not clear.

We thank the reviewer for helping us highlight the added value of the asymmetric electrostatic Yukawa potential. To clarify this important point, we have carried out

additional simulations of systems that rely on electrostatic interactions to undergo phase-separation but with models that use the symmetric Debye-Hückel potential (Mpipi and the HPS-cation-π model). We have also developed a modified version of the Mpipi-Recharged that replaces the asymmetric Yukawa potential with a symmetric Debye-Hückel potential (herein Mpipi-Recharged-symmetric).

In Fig. 4d we provided the salt-dependent phase diagram of H1+ProTα predicted by Mpipi-Recharged directly compared to experiments, and reporting an excellent quantitative agreement. In our new tests, we observe that the HPS-cation-π model reports no phase separation above 100mM of NaCl in disagreement with the experiments, and significantly overestimating the diluted protein concentration (please see new Fig. S10). Furthermore, when used the **Mpipi-Recharged-symmetric** model to probe the phase behaviour of the H1-ProTα system, we observed no phase separation at all at any of the studied salt concentrations (Fig S6). The details of these simulations are provided in section SIX and discussed in page 19.

We then turned to DDX4. With the Mpipi-Recharged-symmetric model, we computed the phase diagrams of the four different DDX4 variants at two different salt concentrations (Fig. S6). As shown in Fig. S6, using the symmetric Debye-Hückel potential leads to a significant underestimation of the critical solution temperatures with respect to the experimental values (more than 45K difference). Despite this, at 100mM of NaCl, the relative phase separation propensity of the four variants is qualitatively predicted, at 300mM the CS variant presents an equal to higher critical temperature than the WT sequence, which is contrary to what has been experimentally observed. We discussed this point in page 14.

Apart from these additional tests, the simulations discussed above for RNA and provided in the Supporting Material using the original Mpipi and the HPS-cation-π models (Figs. S11 and S12) also support the implementation of an asymmetric electrostatic potential instead of discrete charges. All this substantial body of results comparing the Mpipi-Recharged model against in vitro experiments further confirm the modelling electrostatic repulsion vs. attraction asymmetrically in a residue-resolution coarse-grained model improves the description of charge effects in biomolecular condensates.

HPS-cation-π:

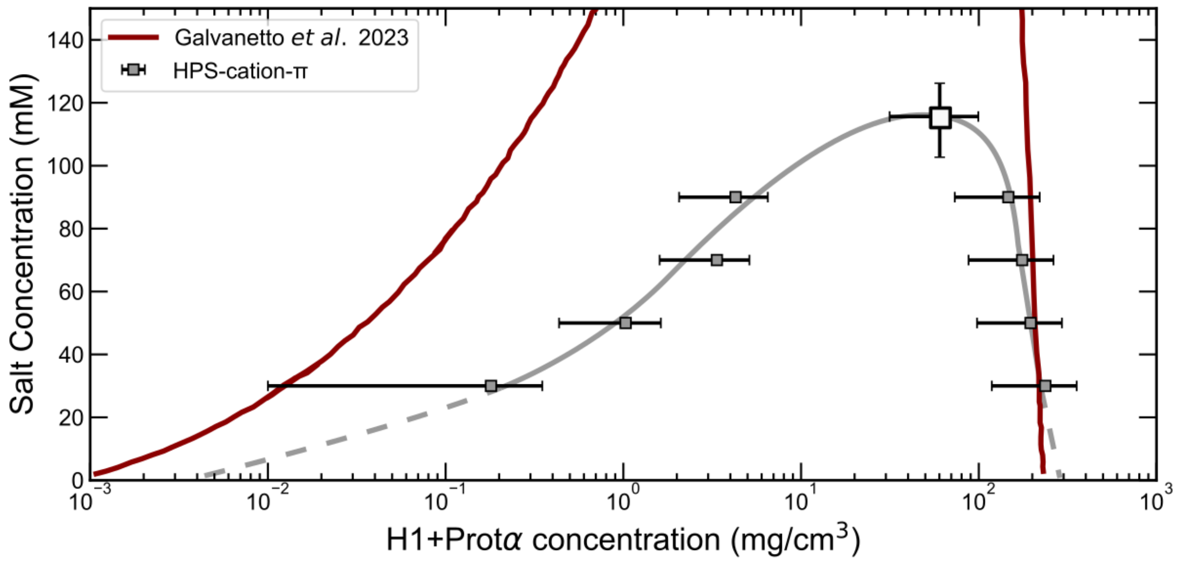

Mpipi-Recharged-symmetric: No phase separation

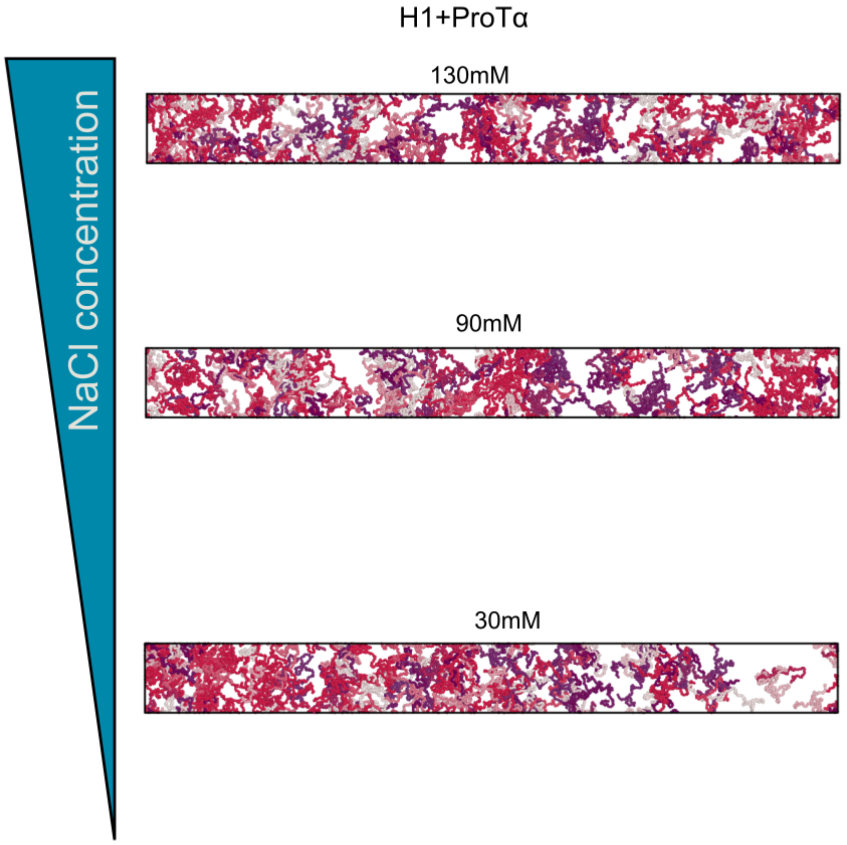

Mpipi-Recharged:

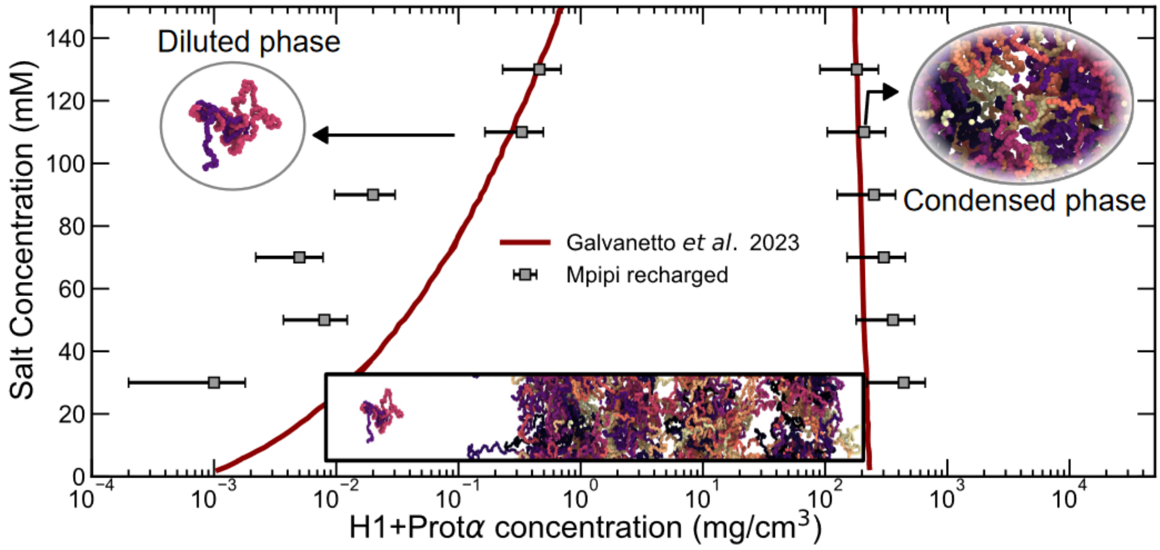

Simulations of DDX4 using the Mpipi-Recharged with the charges of the Mpipi model:

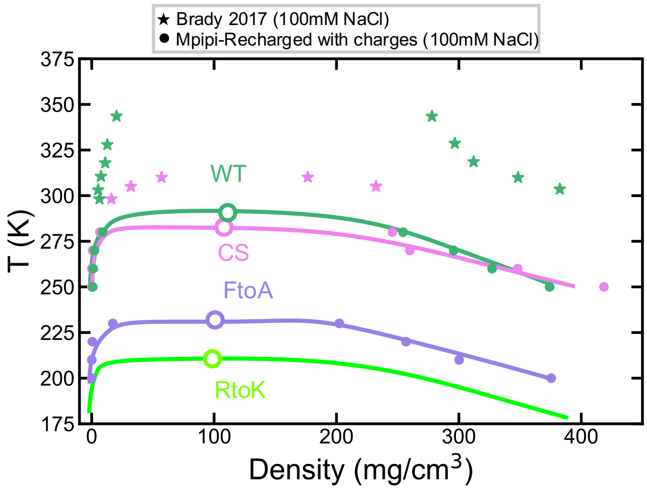

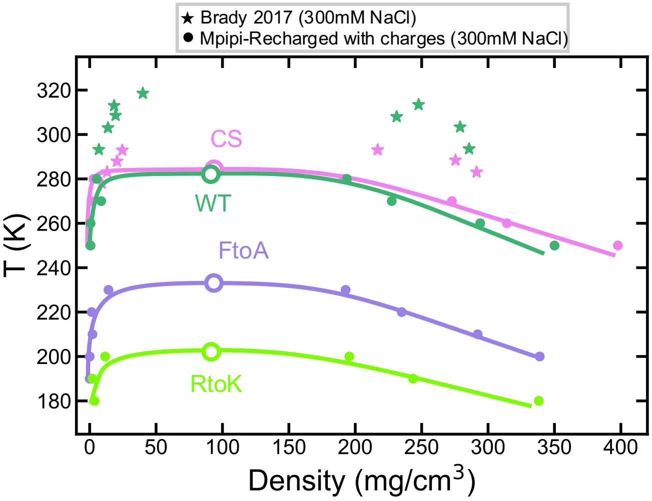

Mpipi-Recharged simulations (Figs. 3a and 3b):

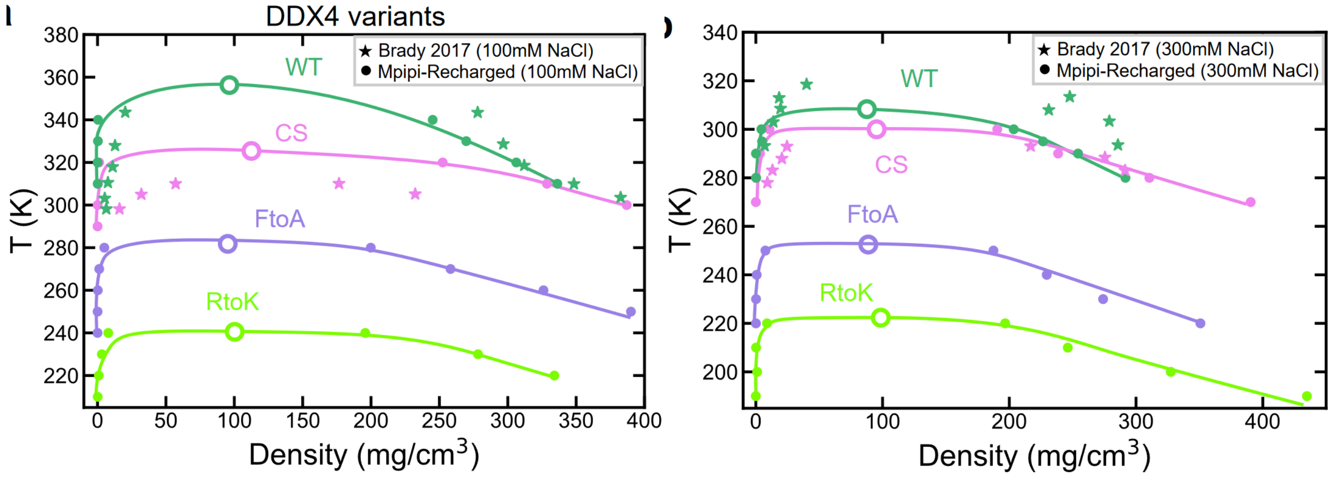

Reviewer 2

In this manuscript, the authors present the Mpipi-Recharged model, an implicit-solvent, residue-resolution coarse-grained (CG) model aiming to resolve challenges in describing charge effects in biomolecular phase behavior present in predecessor models (such as Mpipi). Instead of using a symmetric description of charge–charge interactions, the model adopts an asymmetric, pair-specific Yukawa potential to recapitulate the PMF of atomistic simulations of explicit solvent and ions they've sampled, enabling the accurate capture of asymmetry and phase behavior of highly charged proteins and complex coacervates. The authors validate the model against the same experimental results used to validate the Mpipi model, such as single-molecule radii of gyration of many IDPs, critical solution temperatures, and saturation concentrations. The model also finds agreement with experimental results for descriptions of charge-blockiness, a challenge to many residue- resolution CG models.

Overall, the manuscript demonstrates an improvement from a class of implicit-solvent CG models aimed to investigate phase behaviors in solvated biomolecular systems and are in general congruence with experimental results. However, I provide here several major and minor issues the authors should address:

We thank the reviewer for the positive feedback of our paper. Below, we address point by point the different comments and suggestions raised by the reviewer.

Major comments:

• The authors should report standard errors on all plots of PMFs, such as Figure 1.

We thank the reviewer for this suggestion. We have included error bars in Fig.1, their calculation is described in the corresponding caption and discussed in page 10.

• The authors should discuss the performance of this model with available, machine-learned, implicit-solvent models for similar biomolecular systems of interest, such as the model presented by Airas J, Ding X, and Zhang B, ACS Cent Sci, 2023.

We thank the reviewer for providing this important reference and for suggesting to add further discussions on implicit-solvent models and machine-learning modelling approaches in biomolecular systems. Indeed, the lack of an explicit solvent in the Mpipi-Recharged (and similar residue-resolution models) impedes an accurate description of certain phenomena such as condensate solvation, water entropic effects, or ion density fluctuations inside the condensate and in the diluted phase. However, the computational cost of an explicit-solvent model is too expensive and still, our implicit-solvent approach is able to capture the behaviour of highly charged systems that undergo LLPS driven by electrostatic interactions (e.g. DDX4 variants, A1-LCD charged variants, RNA-protein mixtures). Moreover, phase diagrams as a function of salt concentration are quantitatively recovered (e.g., the H1-ProTα mixture). The reference provided by the reviewer proposes a highly interesting strategy to optimize the interaction potential of implicit-solvent coarse-grained models using graph neural networks which generate solvation free energy estimations more accurately than state-of-the-art implicit solvent models, and describe configurational distributions of explicit solvent simulations. We apologise for having missed this highly relevant reference since it contributes to improving the physicochemical realism of implicit-solvent models without increasing their computational cost. We have cited and discussed this reference among few others in the new Section "Limitations of the Mpipi-Recharged" starting in page 25.

Minor comments:

• When abbreviating terms, the authors should introduce the full term before using its abbreviations, such as "IDRs" ("intrinsically disordered regions") and "IDPs" ("intrinsically disordered proteins").

We thank the reviewer for noticing this problem. We have appropriately introduced both abbreviations pointed out by the reviewer in the introduction in page 3. Furthermore, we have revised the entire manuscript to ensure that all abbreviations are properly defined.

• The authors should abbreviate terms that are repeated many times, such as "molecular dynamics" (MD), "coarse-grained" (CG).

We thank the reviewer for this suggestion that will improve the readability of our manuscript. We have replaced many repeated terms for abbreviations, as well as suggested by the reviewer.

• The authors should not abbreviate terms that are only used one time, such as "Weighted Histogram Analysis Method."

We thank the reviewer for noticing this. As suggested, we have removed this abbreviation and revised the entire manuscript.

• On page 6, the authors misspelled "asymmetric."

We thank the reviewer for noticing this typo. We have corrected it and checked the full text.

• The authors did not provide section numbers after many instances of phrase "see section," such as on pages 8, 12, 18, and 21.

We kindly thank the reviewer for pointing out this error. We have corrected those section references accordingly and make sure that no more section references are missing.

Reviewer 3

The authors present one step further in their efforts to characterize condensates, especially those leading to liquid phases related to IDPs and structure-less nucleic acids. The work is focused on electrostatic model. The authors followed ideas of already existing models (including their Mpipi one) which use a high level of coarse graining, where details of the side chains are ignored. The idea here is to use a slightly different electrostatic description that is parametrized using atomistic simulations as reference and is validate with a large range of macroscopic descriptors of disordered proteins and some phase diagrams. The paper is very interesting, and there is a huge amount of work presented here. I believe that it merits publication, but I believe the authors should take into consideration comments below:

We thank the reviewer for considering our work as an important contribution to the field and for recommending it for publication. Below, we address point by point the different comments and suggestions raised by the reviewer.

There is a huge amount of papers on the **biases of atomistic simulations of charge-charge** interactions. Some authors use specific tuning of van der Waals terms to correct over-stabilization of complexes, others played with more polar solvent models, even an increasing number of authors scale down directly the charges (following a complex reasoning on effective dielectric constants). All this tuning-activity activity reflects the **unpleasant fact that atomistic representations of ion pairs are not necessarily correct**, and the **authors should provide evidence that they are representing well the experimental reality**. For example, lysine acetate or arginine acetate are (I think) soluble in water. Can author's simulations represent this? On the same lines, the minimum of a

We thank the reviewer for raising this relevant comment. As pointed out by the referee, atomistic PMF calculations involving ions are delicate calculations that need some

caution, especially when involving ions at moderately high concentrations. However, the systems we simulate in this work are at low salt concentrations (150mM NaCl concentration) to describe physiological salt conditions. Therefore, the number of ions in our system is well below the solubility of the employed water-NaCl force field, and hence, no need for charge rescaling is required to avoid spontaneous ion precipitation as seen at higher salt concentrations (i.e., 3-6M NaCl concentration, see for instance a further discussion on this topic in Ref. 10.1063/1.5121392.) To discard any possible force field artifact arising from the chosen water-NaCl force field, we have represented the predicted equation of state of the model as a function of salt concentration against the experimental densities in the following figure:

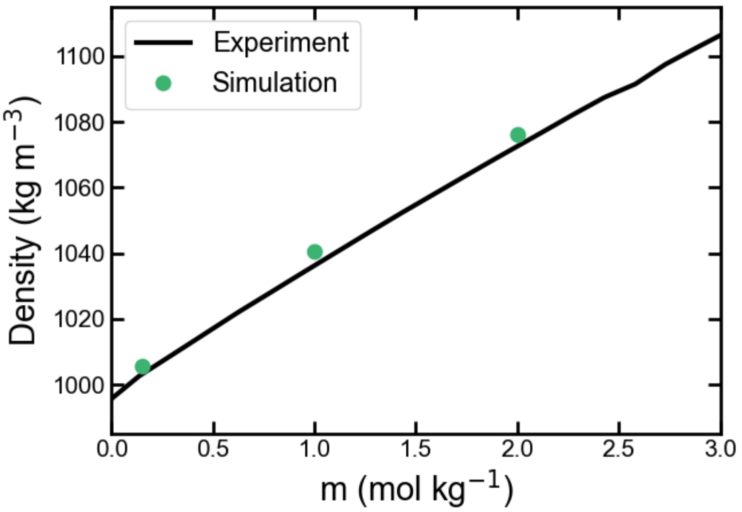

The agreement is highly satisfactory at the concentration of 150 mM of NaCl, and as expected, starts to deviate beyond higher concentrations (i.e., 2M). Nevertheless, we note that the residue-residue intermolecular interaction is the main feature we aim to quantify and understand through our all-atom PMF calculations (as previously performed by us in Refs. Krainer et al., Nat. Communs., 2021; Joseph et al., Nat. Comput. Sci., 2021; and Torrino et al., Cell, 2024). Importantly, we note that the PMFs are not directly converted into the model parameters, since the PMF curves represent the overall interaction free energy for specific configurations, reflecting not just electrostatic contributions but also other forces, such as van der Waals and hydrophobic interactions. Specifically, as we did for parameters of the Wang-Frenkel potential in the original Mpipi model before (Joseph et al Nat Comp Scie 2021), we compute the integrals of the well depths in our atomistic PMFs and normalise them by the value of the strongest interacting pair (in this case R-D). Then we adjust the values of the prefactor A_{ij} by a suitable multiplicative factor so that the normalized PMF integrals are reasonably approximated by the normalized integrals of the Mpipi-Recharged model (considering both the Yukawa and the Wang-Frenkel contribution). This comparison is shown in Fig. S16.

We then validate the model by comparing against single-protein radius of gyration of many IDPs (reported in the Supporting Material), coexistence lines of numerous A1-LCD (charged and non-charged) mutants in Fig. 2, the phase behaviour of DDX4 mutants and charged R12 variants in Fig. 3, or phase diagrams of mixtures of polyUridine and short engineered peptides in Fig. 5. Furthermore, we test against in vitro experiments of the H1-ProTα complex coacervate (Fig. 4), and several RNA-binding proteins in presence of polyuridine (Fig. 5). We have clarified this point regarding the model parametrization strategy of the Mpipi-Recharged in pages 11 and 33.

PMF should not be interpreted as a binding free energy, **the PMF needs to be integrated**, and this is especially important for multiple minima PMFs. Caution is needed as **this seems to be a bottom-up coarse-grained model**. I would recommend some checking of the reference calculations.

We thank the reviewer for highlighting a point that we needed to clarify. We completely agree with the reviewer regarding the integration of the PMFs for calculating the cumulative effective interaction of residue pairs across distance. In fact, as decribed above, this approach is employed since in our previous publication of the Mpipi model (Joseph et al., Nat. Comput. Sci., 2021) to balance the relative interactions among amino acid pairs. We have added in Fig.1d the calculation to quantify the relative interaction strength of residue pairs based on PMF integration. The relative interaction energy obtained from the PMFs minima was moved to Fig. S2. We also compare the Mpipi-Recharged potential to the PMFs (minima and integrals) in Figures S15 and S16. The results in these new figures confirm that both approaches yield consistent outcomes and demonstrate a clear asymmetry between the electrostatic repulsion and attraction of charged residues.

Eq. 2 is extended to all bonds, understanding sequential contacts only, right? no need to add additional terms? Do they reproduce the sampled **"bonded" geometries of IDPs** in atomistic simulations (using a IDP-friendly FF)? Can the authors comment on this? I am curious on the need to **use WF potential for vW interactions**. It is very complex, and probably not the most efficient one from a computational point of view. Many fitted parameters improve flexibility, but also the **risk of overtraining**. Can the authors comment on this?

We thank the reviewer for commenting on this point. The interaction between consecutive amino acids across the sequence (belonging to a disordered region) is given by a harmonic bond potential as noted by the reviewer. Further intramolecular interactions such as bending, dihedral or improper constraints are not included neither in the proteins nor in the RNA molecules. For the globular regions in multi-domain proteins, we use a rigid body integrator to impose the structure of the globular domain (extracted from the PDB database) across the entire simulation. In both RNAs and

proteins, intramolecular interactions between consecutive residues (i.e., 1-2 interactions) are not considered. Regarding the vW interactions, we have used the Wang-Frenkel potential for the the greater flexibility of its functional form in describing protein-protein, protein-RNA, and RNA-RNA interactions depending on the nature of the interacting residues. This potential offers the possibility of describing purely repulsive interactions (which cannot be attained with a standard Lennard-Jones potential), as well as modulating the steepness of the potential minimum, its width and depth, and also the potential range. Such versatility is highly beneficial when describing substantially different interacting modes as those displayed by the different types of amino acids and nucleotides, as discussed in the original Mpipi model (Joseph et al., Nat. Communs., 2021). Moreover, the WF potential possesses a similar computational cost to the standard Lennard-Jones potential. We also note that the Ashbaugh-Hatch potential is highly versatile for describing hydrophobic interactions as employed in other coarse-grained models such as in the CALVADOS, and HPS family models. Nevertheless, beyond the Wang-Frenkel potential, which can offer high variability in the depth, width, shape, and range of the specified interactions, the critical improvement for the model performance is abandoning the well-established Lorentz-Berthelot mixing rules. This enables a much larger range of potential parameters to describe cross-interactions between different types of residues. We have included a discussion regarding these points raised by the reviewer in the Methods Section (pages 30-32).

I must recognize that I did not know Yukawa potential, but looking at eq. 6, it looks like a scaled Debye Huckel, where the term QQ/epsilon is a fitted variable. Could they discuss about what is the effective charge that are implicit to the fitted Aij terms depending on the pairs? This will help to convince on the lack of biases in training. The authors should discuss how much of the fitted parameters are dependent on the vW terms they are using. Playing simultaneously with effective size and the charge improve fitting capabilities, but also overtraining risks. Some calculations on the final model using small systems would help to convince the reader. Format of some references are wrong.

We thank this comment raised by the reviewer. As indicated by the reviewer, our interaction parameter A_{ij} is a fitted variable that can be directly converted to the Coulombic kq_iq_j/ϵ term, giving an effective charge product (q_iq_j) to our electrostatic potential where *k* is the Columbic constant (1/4$\pi\varepsilon_0$) and ϵ refers to the dielectric constant of the medium. We have included a discussion of this point in the Methods section where the model is defined (page 33). In addition, we have incorporated a supporting table in the Supplementary Material where we provide the values of the effective charges (Table S4). The parameter A_{ij}, when considering a Coulombic-like potential (e.g. as the Debye-Hückel potential in the HPS and CALVADOS models), has a value of A_{ij}=4.145 kcal $\mathrm{mol}^{-1}\text{Å}$, assuming charges $q_i = q_j = 1$, for all charged residues (K, R, D, and E) except for histidine. Therefore, the magnitude of the parameters A_{ij} used for the Mpipi-

Recharged are of the same order as in previous models with punctual charges (see Table S3). In Table S4, we show how the values of the product $q_i q_j$ between K, R, D, and E range from +0.963 e^2 (for repulsive electrostatic interactions) to -1.187 e^2 (for attractive ones). That shows how the charge rescaling of the Mpipi-Recharged is quite subtle, and qualitatively follows the relative interaction strengths obtained from all-atom PMF simulations. For interactions between histidines, $q_i q_j$ ranges from +0.25 e^2 to -0.593 e^2. We also note that, as in all the different residue-resolution previous parametrizations, these effective values for the electrostatic interactions between charged residues are modulated by mid-range vW interactions (in this case implemented through the Wang-Frenkel potential). We have included a new Fig. S14 in which we show the relative interaction strength between all the different types of amino acids in the model, including and excluding the Yukawa interactions from the vW contribution. A discussion on this point has been included in section SXII of the Supporting Information, and in page 33 of the Methods Section. The significant improvement of partially rescaling the charges through the Yukawa potential can be noticed in the results displayed in the new Figs. S5, S6, S10, S11, and S12.

In summary, despite my comments above, I am very positive on this paper.

We kindly thank the reviewer for the positive evaluation of our work and the constructive comments to improve the quality of our work.

oc-2024-016178.R2

Name: Peer Review Information for "Chemically-informed coarse-graining of electrostatic forces in charge-rich biomolecular condensates"

Second Round of Reviewer Comments

Reviewer: 1

Comments to the Author

The authors have done a great job in addressing my previous concerns, and I am therefore happy to recommend publication of this important work.

Reviewer: 3

Comments to the Author

They respond well to my comments. Great work, but I would like to see how the method reproduce ammonium acetate solutions (they were a nightmare for atomistic simulations)

Author's Response to Peer Review Comments:

In the revised version we have included a synopsis, a graphical abstract and the supporting information information paragraph as indicated. Furthermore, in the Data avilability section, we have provided a repository with the code necessary to run the simulations presented in our work and an implementation of the Mpipi-Recharged in OpenMM.

We have replied to the Referee 3 query in the attached file.

Reviewer 3

It was just a minor point. I pointed that it will be great to see if the model is shown to be able to reproduce association of an acidid residue with Lys or Arginine. The objective is to determine whether they aggregate in solution or not, that is a risk when A DebyeHuckel like interaction is

used. CG models similar to that developed by the authors tend to consider charged residues very sticky and not sure this is real of an artifact of the fitting.

This is why I made the parallel of NH+ Cl- pr Acetate-Amonia that are a mimick of Asp and Lys. I am not asking to parametrize ammonia or acetate, just see how the residue potentials behave in a case that is characterize: Asp and Lys should not bind in solution and might bind in a desolvated environment.

Hope I explained myself better.

We acknowledge the comment raised by the reviewer regarding the parameterization of the electrostatic interactions. Following the reviewer's advice we have carried out the following tests: 1) We have simulated a direct coexistence slab containing short charged peptides polyK10-polyD10 at 300K and 150mM of NaCl (Fig. S17); and 2) we have calculated the most frequent intermolecular amino acids contacts involved in the H1:ProTα condensate (Fig. S18). The first simulation demonstrates that our model does not yield an effective binding of lysine and aspartic acid in solution, leading to no phase separation. On the other hand, analysing the most frequent amino acids contacts, we obtain that KD and KE notably contribute to sustaining LLPS within a biomolecular condensate, i.e. a desolvated environment. These new figures and calculations have been included in the supplementary information (Figs. S17 and S18) and have been discussed in page 22. We thank the reviewer for this thorough insight regarding our model parameterization.
